# Selective Modification for Red‐Shifted Excitability: A Small Change in Structure, a Huge Change in Photochemistry

**DOI:** 10.1002/chem.202003672

**Published:** 2020-12-23

**Authors:** Yvonne Becker, Sina Roth, Maximilian Scheurer, Andreas Jakob, Daniel A. Gacek, Peter J. Walla, Andreas Dreuw, Josef Wachtveitl, Alexander Heckel

**Affiliations:** ^1^ Institute for Organic Chemistry and Chemical Biology Goethe University Frankfurt Max-von-Laue-Str. 7 60438 Frankfurt am Main Germany; ^2^ Institute for Physical and Theoretical Chemistry Goethe University Frankfurt Max-von-Laue-Str. 7 60438 Frankfurt am Main Germany; ^3^ Interdisciplinary Center for Scientific Computing (IWR) Theoretical and Computational Chemistry Im Neuenheimer Feld 205A 69120 Heidelberg Germany; ^4^ Institute for Physical and Theoretical Chemistry Technical University Braunschweig Gaußstr. 17 38106 Braunschweig Germany

**Keywords:** charge transfer, computational chemistry, fluorescence, photochemistry, photolabile protecting groups

## Abstract

We developed three bathochromic, green‐light activatable, photolabile protecting groups based on a nitrodibenzofuran (NDBF) core with D‐π‐A push–pull structures. Variation of donor substituents (D) at the favored ring position enabled us to observe their impact on the photolysis quantum yields. Comparing our new azetidinyl‐NDBF (Az‐NDBF) photolabile protecting group with our earlier published DMA‐NDBF, we obtained insight into its excitation‐specific photochemistry. While the “two‐photon‐only” cage DMA‐NDBF was inert against one‐photon excitation (1PE) in the visible spectral range, we were able to efficiently release glutamic acid from azetidinyl‐NDBF with irradiation at 420 and 530 nm. Thus, a minimal change (a cyclization adding only one carbon atom) resulted in a drastically changed photochemical behavior, which enables photolysis in the green part of the spectrum.

## Graphical abstract

**What a difference one atom makes**: Azetidine (az) and dimethylamino (DMA) donor moieties differ only by one carbon atom in their molecular formula, but in the present study we observed huge differences in their impact on the photochemistry of D‐π‐A caging groups. Restriction of the degrees of movement freedom—due to the four‐membered ring—leads to a photocage that can be used within the “green gap” for the release of biologically relevant leaving groups (e.g., glutamic acid).

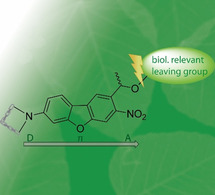

## Introduction

Due to their advantage of spatiotemporal control without the use of additional chemical reagents, photolabile protecting groups (PPGs) or “caging groups” already created an extensive pool of applications in the fields of biochemistry,[^1^, ^2^, ^3^] organic synthesis[^3^, ^4^] and even inorganic materials for coated surfaces^[5]^ or hydrogel formation.^[6]^ Nonetheless, the development and synthesis of PPGs, which can be used by irradiation with visible light—optimally within the “phototherapeutic window” (650–950 nm)^[7]^ and thus in living cells without tissue damage—remains one of the main tasks of modern photochemistry. Alternatively, also PPGs which can be activated with visible light within the “green gap” (low absorption of the light‐harvesting complexes of plants) from 500–600 nm are highly desired for new applications in plants.^[8]^ There are several strategies for achieving the necessary bathochromic absorption shift: π‐system extension of the chromophore (maintaining planarity)^[9]^ as well as an attachment of donor (D) and acceptor (A) structures to create a push–pull character^[10]^ that enhances electron delocalization and therefore decreases the energy required for excitation, are few of them. The π‐system strategy has often to deal with solubility issues in aqueous media.

For biologically suitable caging groups, apart from the absorbance properties, another important aspect is the quantum yield of photorelease φr (in competition to alternative relaxation pathways from the excited state) as the uncaging efficiency is determined by the product ϵ⋅φr. A non‐negligible part of the radiation energy is lost for example, by fluorescence emission or non‐radiative decay channels like rotation around single bonds and intramolecular vibrational energy redistribution (IVR).

**Figure 1:**
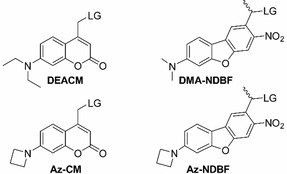
Diethylaminocoumarin (**DEACM**),[^4^, ^24^] azetidinyl‐coumarin (**Az‐CM**),^[11]^ dimethylamino‐nitrodibenzofuran (**DMA‐NDBF**)^[22]^ and the new azetidinyl‐nitrodibenzofuran (**Az‐NDBF**) with LG=leaving group.

Rivera‐Fuentes and co‐workers published a comparison of azetidinyl‐coumarin (Az‐CM) and the widely used blue‐absorbing PPG diethylaminocoumarin (DEACM) regarding their photolysis efficiency (Figure 1).^[11]^ A third derivative carried a julolidine^[12]^ substituent, where rotation around the N‐C (donor) bond was prohibited due to the connection of the 6‐membered alkyl‐rings to the aromatic system. Interestingly, julolidine‐ and azetidinyl‐coumarin showed a highly similar behavior in all experiments. They investigated the photolysis rates of the derivatives depending on solvent polarity and proticity. In water, the derivative with the azetidinyl substituent had a significantly better φr than DEACM because the small heterocyclic ring appears to inhibit photochemically unproductive decay channels, whereas the diethylamino derivative loses the photon energy.

One widespread explanation for this phenomenon of structural influence is intramolecular charge transfer (ICT) state population, another one is the hydrogen bonding (H‐bonding) hypothesis. Both effects strongly depend on the surrounding medium. Charge separation can be stabilized in polar solvents and H‐bonding induces non‐radiative decay in protic solvents. When experiments are performed in water, which is both polar and protic, both may play a role. ICT states in general are a relatively common phenomenon in molecules with a D‐π‐A design. If the intramolecular electron transfer from donor to acceptor leads to a twisting of a single bond, this is referred to as T‐ICT. If the resulting conformational change is not a rotation but rather a planarization—for example of a previously pyramidal amine—this is called P‐ICT.[^13^, ^14^] A population distribution between locally exited (LE) state and a stabilized charge transfer (CT) state may be detectable by a dual fluorescence.[^15^, ^16^] The CT state opens up new relaxation pathways. Knowledge of these pathways and influence of different substituents with torsional angle and bond length restriction possibilities can be exploited for novel PPG design.[^17^, ^18^]

Apart from the photorelease studies of Az‐CM, azetidinyl substituents in general have been known in the literature for their positive effects on the photochemistry of rhodamines for a long time.^[19]^

## Results and Discussion

The nitrodibenzofuran (**NDBF**) core had originally been introduced as a PPG by Ellis‐Davies.^[20]^ In a previous publication guided by theoretical predictions^[21]^ we presented the improved dimethylamino‐NDBF (**DMA‐NDBF**) group,^[22]^ which showed a surprising excitation‐specific behavior: A one‐photon (1P) irradiation into the red‐shifted main absorbance band around 420 nm did not afford any photolysis any more (φ_420_ <0.05 %) in contrast to **NDBF** (φ_420_=13.6 %), while two‐photon (2P) irradiation at 840 nm was very effective (17 times better than **NDBF**). Our present study contributes to the understanding of the reason for this unusual behavior which might not be a rare case but rather a rarely recognized one.^[23]^ Preferably, we wanted to maintain all the positive properties like the red‐shift and high *ϵ*, to obtain a desirable example of a green‐light activatable PPG which can be cleaved by 1PE. Azetidinyl N(CH_2_)_3_ should act as a donor substituent at ring position 7 (**Az‐NDBF**), replacing NMe_2_ in **DMA‐NDBF** ^[22]^ (Figure 1).

**Figure 2:**
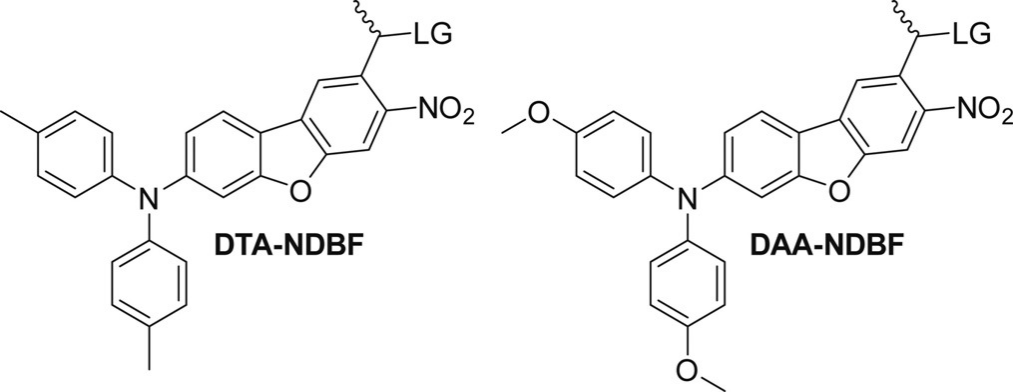
Di‐(*p*‐tolyl)amino‐NDBF (**DTA‐NDBF**) and di‐(*p*‐anisyl)amino‐NDBF (**DAA‐NDBF**) with LG=leaving group.

Additionally, we included two aryl‐NDBF derivatives into this investigation to study the influence of a) stronger electron donors^[25]^ and b) spatially demanding substituents on the photochemistry. Based on a previous theoretical study for the optimal substitution pattern of NDBF for new PPGs with bathochromic shift,^[21]^ we chose the tolyl‐ and anisylamino derivatives shown in Figure 2. This previous study concluded that only donor‐attachment at the 7‐position led to improved performance and that additional substitutions had no substantial further effect.

Also for this study, we started out with time‐dependent density functional (TDDFT) calculations for initial characterization. Ground state equilibrium structures, vertical excitation energies ω_ex_, oscillator strengths f_osc_ as well as 2P absorption probabilities, which are directly convertible to the absorption cross‐section δ_a_ in GM were obtained at the CAM‐B3LYP/def2‐svp level of theory.

Table 1 summarizes the results for the transition from S_0_ to the three energetically lowest singlet excited states (S_1_–S_3_) of **DMA‐** and **Az‐NDBF‐OH** and the two phenyl‐candidates **DTA‐** and **DAA‐NDBF‐OH**. While **DMA‐** and **Az‐NDBF‐OH** show only slight differences in our theoretical calculations, except for their oscillator strength f_osc_ to the S_1_ state, the phenyl moieties of compounds **DTA‐** and **DAA‐NDBF‐OH** have a pronounced effect. They lead to remarkably high predicted absorption cross‐sections of 259 and 296 GM, respectively. In comparison, **NDBF‐OH**, which has already been used in living cells, has a calculated value of 0.14 GM (630 nm) with our method and—coupled to EGTA as Ca^2+^ releasing agent—an experimentally measured value of 0.6 GM (710 nm).^[20]^ Going from **NDBF** to **DMA‐NDBF** we had experimentally observed a red‐shift of >100 nm.^[22]^ Here, a further aryl substitution to compounds **DTA** and **DAA‐NDBF** was calculated to afford only a small additional red‐shift of 18–23 nm.

**Table 1:** Calculated values for the vertical excitation energies ω_ex_, the one‐photon oscillator strengths f_osc_ and the two‐photon absorption cross‐section δ_a_ at the given wavelength for the transition to the three energetically lowest excited states S_1_–S_3_ of **DMA‐**, **Az‐**, **DTA‐** and **DAA‐NDBF** (LG=OH).

Transition	S_0_→S_1_	S_0_→S_2_	S_0_→S_3_
**DMA‐NDBF‐OH**			
ω_ex_ [eV]	3.74	4.14	4.29
f_osc_ [a.u.]	0.57	0.12	0.03
δ_a_ [GM] (λ [nm])	118 (663)	41.7 (599)	3.69 (578)
**Az‐NDBF‐OH**			
ω_ex_ [eV]	3.72	4.14	4.30
f_osc_ [a.u.]	0.62	0.12	0.03
δ_a_ [GM] (λ [nm])	132 (667)	42.1 (600)	2.99 (577)
**DTA‐NDBF‐OH**			
ω_ex_ [eV]	3.55	4.07	4.24
f_osc_ [a.u.]	0.79	0.02	0.02
δ_a_ [GM] (λ [nm])	259 (699)	20.3 (610)	0.54 (584)
**DAA‐NDBF‐OH**			
ω_ex_ [eV]	3.50	4.06	4.21
f_osc_ [a.u.]	0.78	0.02	0.04
δ_a_ [GM] (λ [nm])	296 (708)	23.2 (610)	3.87 (589)

**Scheme 1:**
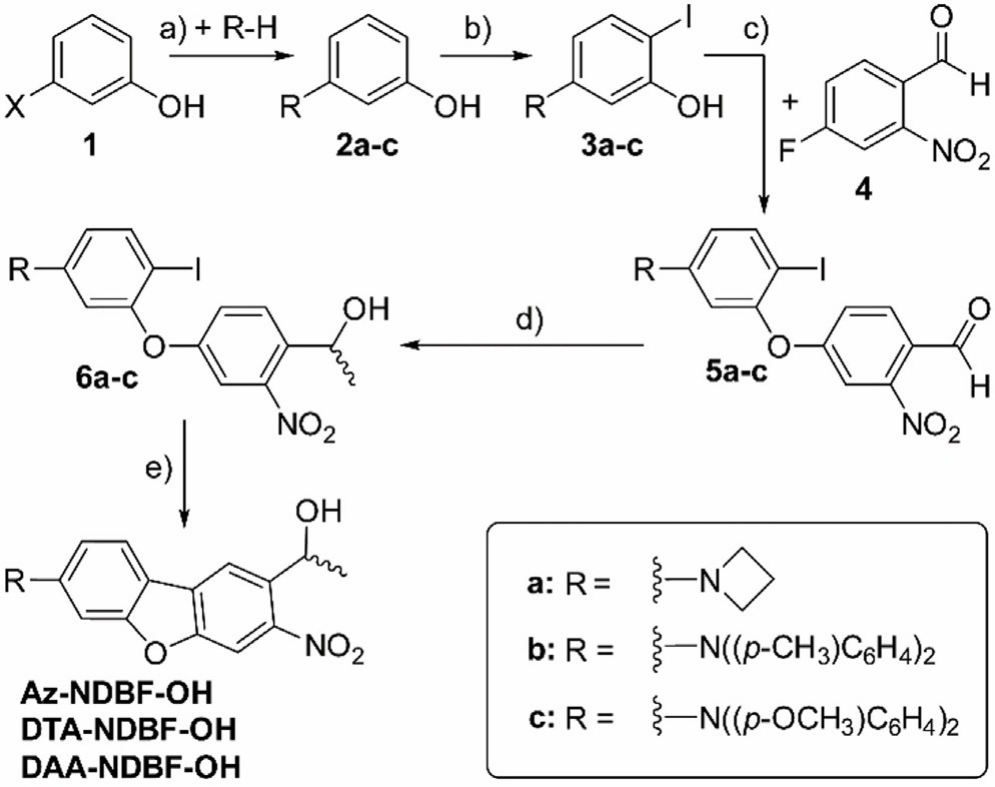
Synthesis of the new *ortho*‐nitrobenzyl photocages **Az‐**, **DTA‐** and **DAA‐NDBF‐OH**: a) LiHMDS, Verkade base, Pd(OAc)_2_ or NaO*t*Bu, P(*t*Bu)_3_, Pd(OAc)_2_ in heated toluene; b) NIS, MeCN, −10 °C→RT, overnight; c) KO*t*Bu, DMSO, RT, 1–2 d; d) Al(CH_3_)_3_, CH_2_Cl_2_, 30 min, 0 °C; e) Cs_2_CO_3_, Pd(OAc)_2_, H_2_O, DMAc, 2–5 d, 80 °C. See Supporting Information for purification, yields, and analytical data. X=I or Br.

An overview of the synthesis routes is given in Scheme 1. As first synthesis step, all three **NDBF** derivatives have a Buchwald–Hartwig cross coupling between *m*‐halogenated phenol (**1,** X=iodide or bromide) and a secondary amine (**a**: azetidine, **b**: di‐*p*‐tolylamine or **c**: di‐*p*‐anisylamine) in common. This derivatization was followed by iodination in *para*‐position to the corresponding amino function (*ortho* to the phenolic OH) with NIS. The next synthesis steps were largely similar to the synthesis of **DMA‐NDBF** as published earlier.^[22]^ Coupling to 4‐fluoro‐2‐nitrobenzaldehyde (**4**) led to the unsymmetrical aryl ethers **5**, which were subsequently reduced and methylated with trimethylaluminum and afterward hydrolyzed to obtain alcohols **6**. The closed‐ring form is the product of a palladium‐catalyzed intramolecular Heck‐like reaction. The leaving group ‐OH can be varied in further steps.

**Figure 3:**
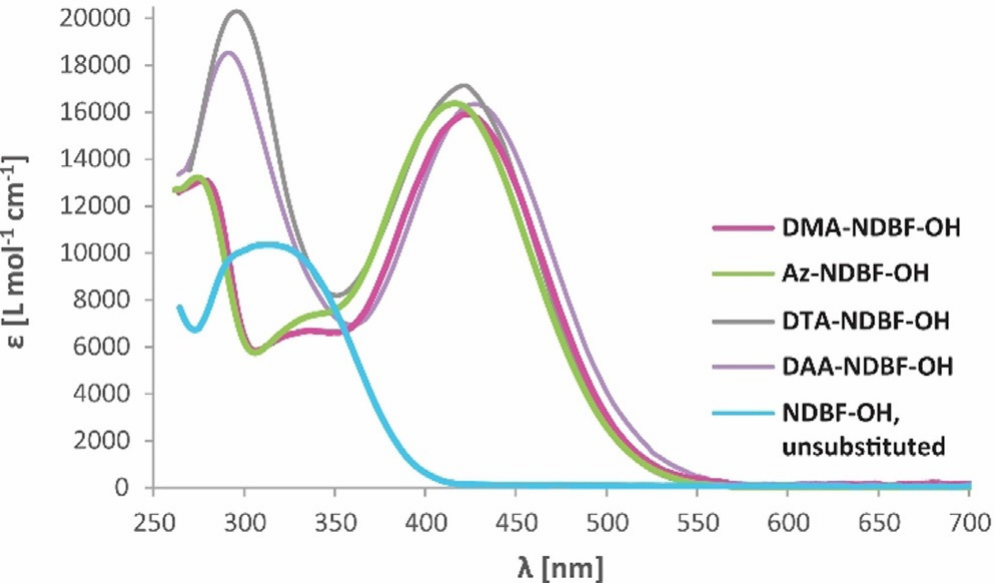
Molar extinction coefficients (*ϵ*) of the alcohols **DMA‐**, **Az‐**, **DTA‐**, **DAA‐NDBF‐OH** in comparison with unsubstituted **NDBF‐OH** in DMSO.

1P‐Absorption spectra of the alcohols were recorded in different solvents (Supporting Information, Figures S4–S6). Figure 3 shows the spectra in DMSO—along with the one of the unsubstituted **NDBF‐OH** (R=H) for comparison. The long‐wavelength absorption maxima are red‐shifted from 312 nm (**NDBF‐OH**) to 422 nm (**Az‐** and **DTA‐NDBF‐OH**) and 426 nm (**DAA‐NDBF‐OH**). As theoretically predicted, the donor variation does not strongly affect the red‐shift, suggesting that we have currently found an optimum of our D‐π‐A system. Thus, also **Az‐NDBF‐OH** showed the expected similar absorption behavior to the one of **DMA‐NDBF‐OH** (*ϵ*_max_=424 nm). Not only the wavelength of the maxima turned out to be similar but also the respective molar absorbance coefficient *ϵ*. Within error limits **DTA‐NDBF‐OH** showed the highest molar absorbance with *ϵ*_422_=17196 L mol^−1^ cm^−1^ in its (second) maximum which is 7 % higher than the one of **DMA‐NDBF‐OH** (*ϵ*_424_=15947 L mol^−1^ cm^−1^).

**Figure 4:**
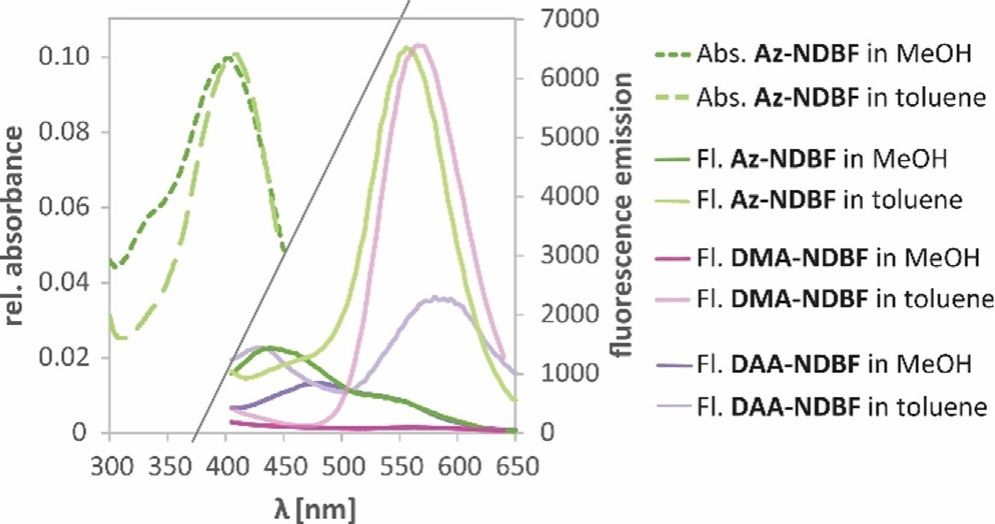
Steady‐state fluorescence spectra of **DMA‐**, **Az‐**, and **DAA‐NDBF‐OH** in aprotic/apolar toluene (light solid lines) and protic/polar MeOH (dark solid lines) with excitation at 340 nm. For comparison, the absorption of the azetidinyl derivative in both solvents is shown (dashed lines left).

We also recorded steady‐state fluorescence emission spectra (Figure 4) to investigate the occurrence of dual fluorescence to probe for the ICT/‐H‐bonding hypothesis. We would expect fluorescence from the LE and CT states.^[26]^ Experimentally, the following was observed: all derivatives showed weak fluorescence signals in general. This corresponds to our TDDFT calculations, which predicted low‐energy transitions to the first excited states. Therefore, non‐radiative decay is very likely.^[27]^ Appropriately, the signals are stronger in less polar solvents such as toluene and weaker in methanol, which stabilizes intramolecular charges. The strongest electron donor, and thereby CT‐supporting derivative **DAA‐NDBF‐OH** has the weakest toluene fluorescence (light purple vs. light pink and green), whereas the other derivatives are similarly bright in toluene. In MeOH **DMA‐NDBF** is completely dark (dark pink line). This means non‐radiative or ultrafast decays dominate, in clear difference to **Az‐NDBF‐OH** (dark green line). This finding is highly interesting as we see photochemical differences in the fluorescence but hardly in the absorption.

**Figure 5:**
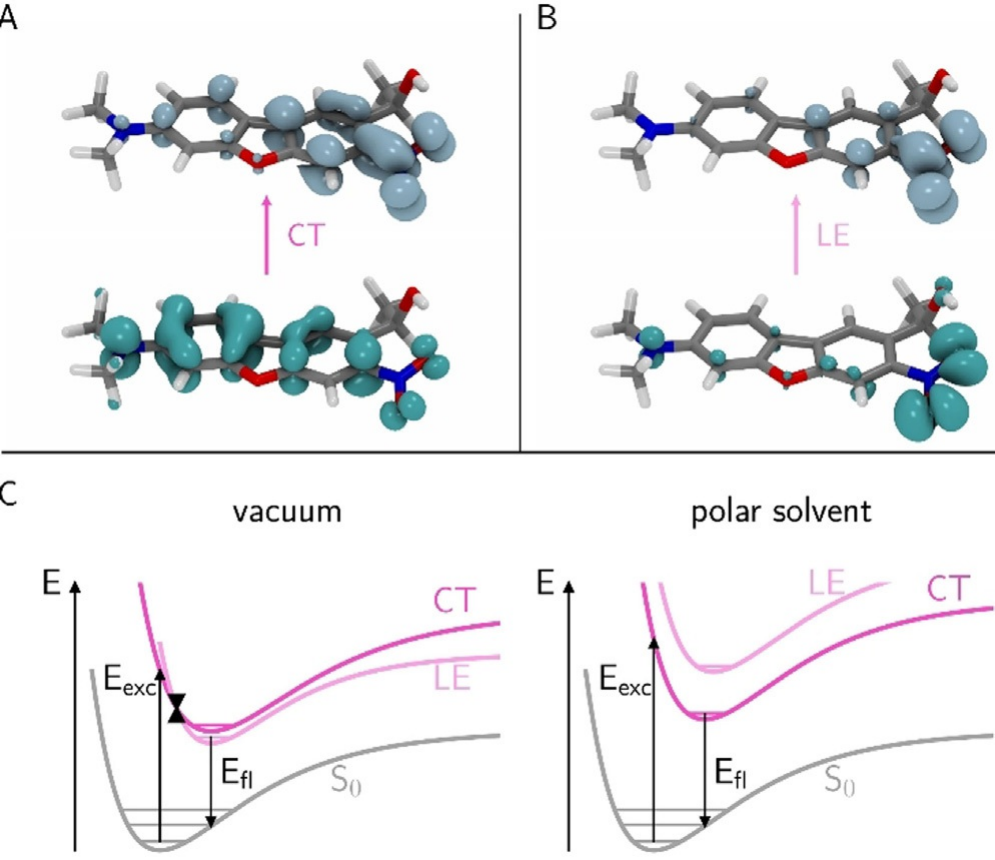
A, B) Electron detachment (lower) and attachment (upper) densities for the energetically lowest excited state transitions of **DMA‐NDBF‐OH**. For the CT state (π→π*) the density shifts from the amino to the nitro group, for the LE state (n→π*) it is localized at the nitro group. C) Franck–Condon diagrams for the S_0_→S_1_ excitation (E_exc_) and the expected relaxation pathway via a conical intersection (CI, black triangles) and the resulting (radiative) decay E_fl_ in vacuum or apolar environment, respectively (left), or in polar solvents (right). For computational methods and data see the Experimental Section and Supporting Information (section 6).

This solvent sensitivity of the two electronically low‐lying excited states was further investigated by calculations and is illustrated in Figure 5. The state characters were assigned by means of detachment and attachment densities (Figure 5 A and B exemplarily for **DMA‐NDBF‐OH**), which demonstrate a shift of electron density from the amino to the nitro group for the CT state, whereas the LE state is localized on the nitro group. Dipole moments of the excited states further corroborate the respective character (Supporting Information). In vacuum (**C** left) and non‐polar environment such as for example, cyclohexane or toluene, the energetically lowest excited state (S_1_), accessible from the ground state geometry, is a CT state. Due to relaxation to the lowest electronically excited state (Kasha's rule) and a conical intersection (black triangles) the NDBF derivatives fluoresce eventually from the long‐living LE state (bright fluorescence) at around 550 nm. The more polar the environment becomes (e.g., MeOH or water), the more the CT state is stabilized and energetically lowered (**C** right), whereas the LE state is higher in energy. The conical intersection disappears and the fluorescence arises from the CT state, which is too fast for steady‐state fluorescence methods.

A publication by the Sølling group,^[28]^ who examined the dual fluorescence of a similar core structure, 2‐diethylamino‐7‐nitrofluorene, can be used for comparison. They computationally identified several excited state minima with CT character, including a P‐ICT state, as well as rotation around the amino group (T‐ICT) and also the nitro group, which accounts for the majority of the non‐radiative decay. They observed complex solvent dependence and ultrafast ISC in some cases (e.g., apolar cyclohexane), which results then in one single fluorescent transition. They stated that the solvent determines not just the fluorescence lifetime, it shapes the potential energy landscape and thereby all relaxation pathways.

Although uncaging mechanisms cannot be predicted by TD‐DFT, one can surely assume that the ultrafast decay pathways prevent the photolysis. In our case, this means that uncaging is only possible from the LE state, which depends on the solvent and donor properties. Hence, the solvent polarity decides if the NDBF derivative will photolyze and if we assume a planar CT state (PICT hypothesis), in the same solvent the planarization of pyramidal N(CH_3_)_2_ should be easier (=lowered CT) than the one of the small heterocyclic azetidine ring due to required bond length changes and ring strain (=high CT, lower LE).

**Scheme 2:**
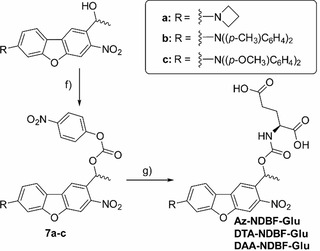
Installation of glutamic acid as leaving group: f) DIPEA, 4‐nitrophenyl chloroformate, CH_2_Cl_2_, overnight or MW; g) l‐glutamic acid monosodium salt monohydrate, DMSO/aq. buffer (pH 8.4) overnight or MeCN/aq. buffer (pH 8.4), MW. Detailed characterization data are provided in the Supporting Information.

For further investigations, whether **Az‐NDBF** is able to suppress the decay channels competing with uncaging in aqueous medium, we attached a water‐soluble leaving group. l‐glutamic acid (**Glu**) was chosen as a polar and biologically relevant leaving group (Scheme 2). Also, for the phenyl derivatives, respective test compounds were synthesized. Therefore, the alcohols **Az‐**, **DTA‐** and **DAA‐NDBF‐OH** were activated with 4‐nitrophenyl chloroformate to afford the active esters **7 a**–**c**. l‐Glutamic acid then replaced the nitrophenyl moiety, resulting in **Az‐**, **DTA‐** and **DAA‐NDBF‐Glu**.

After successful attachment of the amino acid, 1P‐photolysis tests were performed in aqueous buffer (1× PBS, pH 7). For reasons of comparability, the quantum yields for all derivatives were determined at 420 nm.

**Figure 6:**
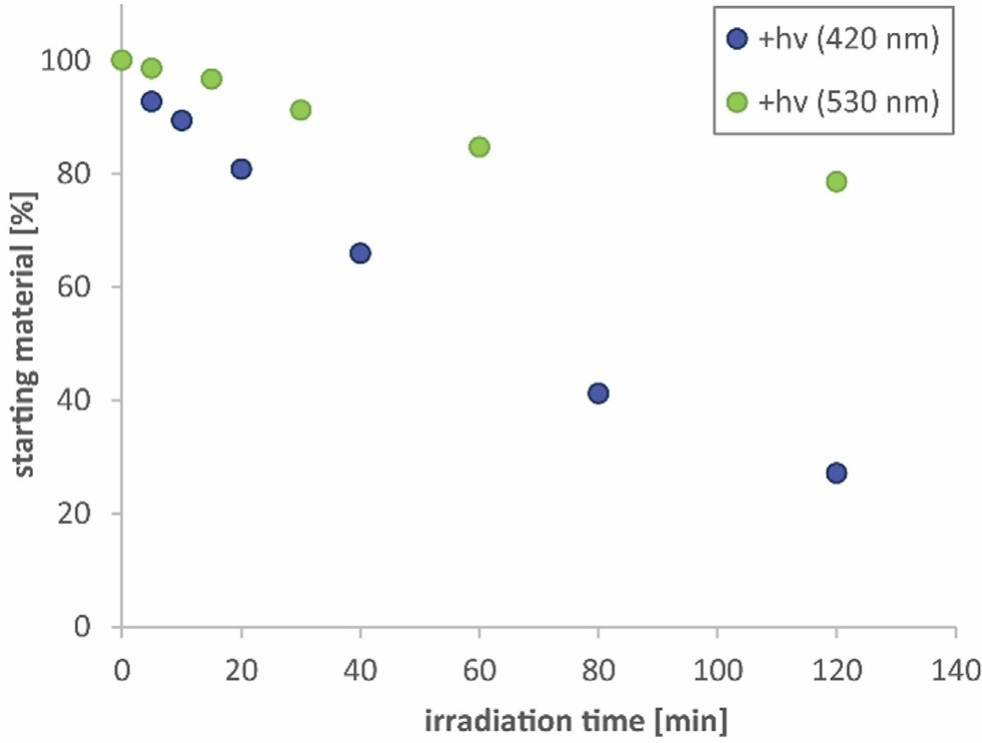
1P‐photolysis of **Az‐NDBF‐Glu** in 1× PBS (pH 7) at 25 °C, 290 μL volume (7.25 nmol, OD_420_=0.21 and 37.3 nmol, OD_530_=0.09), quartz cuvette with 1 cm path length. The mixture included an internal standard (uridine) for HPLC analysis of the amount of starting material. Irradiation was performed with mounted Thorlabs LEDs. The photon flux was determined with a fulgide photoswitch^[29]^ (26.8 nmol s^−1^ at 420 nm, 93.0 nmol s^−1^ at 530 nm).

As an example, a photolysis curve of **Az‐NDBF‐Glu** is shown in Figure 6. After 2 h, only 27 % of the caged compound remained (blue dots). We proved that photolysis of the caged glutamic acid is also possible at higher wavelengths (530 nm, green dots). The quantum yields of our “two‐photon‐only” PPG **DMA‐NDBF‐LG** (LG=Glu or DNA) with excitation light above 455 nm are 0 %. The Φ_420_ values for the phenyl derivatives were determined to be 0.3 and 0.4 %. An explanation can be hindrance of planarization caused by the triphenyl structure if the CT state is of the P‐ICT type.

The decrease of starting material was monitored by HPLC with the use of an internal standard. To obtain the photon flux we used two different methods: ferrioxalate actinometry and our recently published fulgide photoswitch actinometer.^[29]^ The quantum yields of our new PPGs are summarized in Table 2.

**Table 2:** 1P‐photochemical data of the NDBF derivatives.

	*ϵ*(420) [L (mol cm)^−1^]	*ϵ*(530) [L (mol cm)^−1^]	Φ_420_ [%]	Φ_530_ [%]
**DMA‐NDBF**	15 872	843	0.09^[a]^/0.05^[b]^	0^[b]^
**Az‐NDBF**	16 314	718	1.2^[a]^	0.3^[a]^
**DTA‐NDBF**	17 116	565	0.3^[a]^	n.d.
**DAA‐NDBF**	16 220	1104	0.4^[a]^	n.d.

[a] LG=glutamic acid. [b] LG=DNA (dA). n.d.: not determined.

A carbamate linkage has been used before for in vivo 2P‐uncaging^[30]^—its release is slower than carbonates, but it is significantly more stable toward hydrolysis at physiological pH.^[31]^ For a hydrolysis test of **Az‐NDBF‐Glu** see the Supporting Information (Figure S3). After 24 h at 37 °C only 7 % decrease of starting material concentration was observed. Another advantage is the possibility of (spectroscopic) CO_2_ release detection. Ellis‐Davies et al. also studied the release of glutamate which was attached via the carboxylic acid.^[32]^

**Figure 7:**
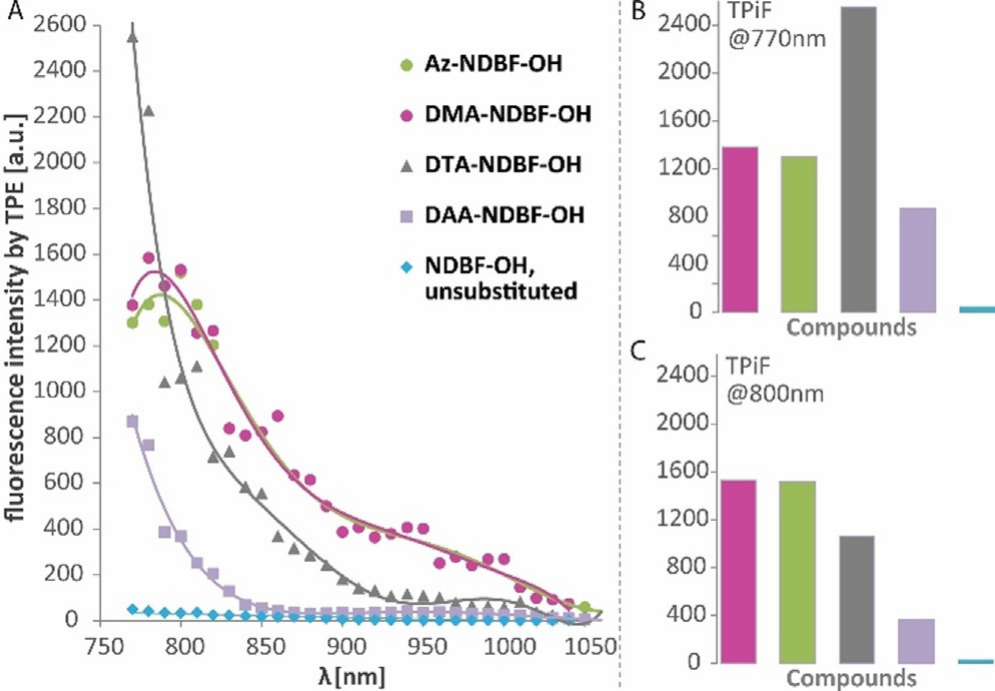
Two‐photon‐induced‐fluorescence (TPiF) spectrum of **Az‐**, **DTA‐** and **DAA‐NDBF‐OH** in comparison with the earlier published **DMA‐NDBF‐OH** and unsubstituted **NDBF‐OH** ^[22]^ in DMSO. A) Resulting fluorescence intensities by excitation between 770 and 1050 nm; B) comparison of the intensities of the various compounds at 770 nm and C) at 800 nm.

For complete characterization, the 2P photochemistry of our derivatives was examined spectroscopically. Two‐photon‐induced‐fluorescence (TPiF) spectra of **Az‐**, **DTA‐** and **DAA‐NDBF‐OH** were recorded in DMSO, which is often chosen in the literature as a viscous polar but non‐protic and thus fluorescence‐promoting solvent,^[33]^ (Figure 7) and compared with **DMA‐NDBF‐OH** ^[22]^ and unsubstituted **NDBF‐OH**.^[22]^

The fluorescence intensity in a.u. is related to the ability to absorb two photons.^[33]^ As we know, a high fluorescence signal is an indicator for few ultrafast decay pathways, which are the main photolysis competitors. In the experiment, all derivatives showed high‐intensity signals within the phototherapeutic window, which make them possible candidates for 2P in vivo applications. In comparison with **NDBF‐OH**, which has already been used for 800 nm 2P photolysis in living cells,^[34]^ the values of the derivatives are 47 (**DMA‐** and **Az‐**), 32 (**DTA**) and 11 (**DAA**) times higher at 800 nm. Up to 800 nm, the stronger donor **DTA**, in comparison with **DMA‐NDBF**, showed the expected higher fluorescence signal. In turn, we have previously shown that TPiF of **DMA‐NDBF** correlates well with its 2P photolysis rate in our strand displacement assay.^[22]^ **Az‐** and **DMA‐NDBF** show again a very similar behavior, so there is no reason to assume that the 2P photolysis for **Az‐** should not be as good as for **DMA‐NDBF**.

## Conclusion

In conclusion, we designed, synthesized and characterized three new representatives for *ortho*‐nitrobenzyl photocages in the present study. Their absorption profiles are bathochromically shifted and within the visible and less tissue‐harmful spectral range maintaining the positive red‐shift‐effect of alkylamino donors. We want to point out, that the spectral shift is still one of the major goals of photochemists for biological applications. However, in strong distinction to our earlier published **DMA‐NDBF**, which is interestingly inert against visible light excitation (“two‐photon‐only” behavior), the best derivative **azetidinyl‐NDBF** is green (1PE) and NIR light (2PE) activatable. Technically, the azetidine and dimethylamino derivatives differ only by one carbon atom in the molecular formula, but we successfully tested the release of a biologically relevant leaving group (glutamic acid) with 420 and 530 nm irradiation in physiological buffer. The two phenyl derivatives showed an intermediate photolysis behavior. Compared with **DMA‐NDBF** they have a higher 2P absorbance cross section. Most importantly, all these findings allowed us to learn more about excitation‐specific photochemistry and optimal PPG design.

## Experimental Section

### Synthesis

In general, all reactions were performed under argon atmosphere and in dry solvents unless otherwise specified. Solvents and reagents were purchased from commercial sources. 3‐(Azetidine‐1‐yl)phenol (**2 a**) was synthesized according to Ref. [19]. **DMA‐NDBF‐OH** was synthesized as earlier published.^[22]^ Preparation of new and unpublished compounds and their characterization are provided in the Supporting Information. Microwave reactions were performed in a Biotage Initiator microwave system with matching Biotage vials. Reaction progresses were monitored by TLC analyses (silica gel 60‐coated aluminum sheets, UV254 marker, Macherey–Nagel). Reaction product purifications were performed via column chromatography with silica gel 60 by Macherey–Nagel or automated flash chromatography with a puriFlash® XS 420 ULTRA system and associated prep. silica gel columns (15 μm or 30 μm) by Interchim. Highly polar compounds were purified by RP‐HPLC (MultoKrom columns by CS Chromatographie). NMR spectra of new compounds were recorded on (250 MHz, 400 MHz, 500 MHz, or 600 MHz) Bruker instruments. ESI mass spectra were obtained with a ThermoFisher Surveyor MSQ and high resolution mass spectra (HRMS) were obtained with a MALDI LTQ Orbitrap XL instrument (ThermoScientific).

### Photochemical measurements

All absorption and fluorescence emission measurements were performed in standard quartz cuvettes (1.00 cm optical pathlength, Hellma‐Analytics) with various maximum volumes. UV/Vis absorption was recorded using a commercially available Evolution 300 (ThermoScientific) or our custom‐made set up equipped with an Ocean Optics DH‐mini light source and USB4000 detector, a thermostatic cuvette holder (Thorlabs), all controlled by our in‐house programmed PHITS (Photoswitch Irradiator Test Suite) software, which was written in LabVIEW. For more details see Reinfelds et al.^[29]^ This setup and software were also used for our chemical actinometry. Reference compound was an indolylfulgide photoswitch. A concentrated solution of the fulgide (500–1000 μm) was irradiated with the respective light source (Thorlabs mounted LED, λ_max_=420 nm or 530 nm) to convert the photoswitch from its **1Z** form to **1C** or the other way round. Afterward, the caged glutamic acid of interest could be irradiated with known photon flux. Steady‐state fluorescence emission was recorded using a Hitachi F‐4500 spectrophotometer. The optical density (OD) was set lower than or equal to 0.1 for fluorescence spectra, otherwise checked for consistency. Details of the set ups for two‐photon induced spectroscopy have been described previously.[^22^, ^35^, ^36^] See the Supporting Information for additional data.

### Computational methods

The molecular geometries of **DMA‐**, **Az‐**, **DTA‐** and **DAA‐NDBF‐OH** were optimized with CAM‐B3LYP/def2‐svp as implemented in the Q‐Chem program package, version 5.2.[^37^, ^38^, ^39^] Frequency analyses confirmed that correct minimum geometries were found. The three energetically lowest singlet excitation energies were obtained with time‐dependent (TD‐)CAM‐B3LYP/def2‐svp using Q‐Chem. Two‐photon transition strengths δ_2P‐ts_ were computed using quadratic‐response DFT employing CAM‐B3LYP/def2‐svp as implemented in DALTON 2018.^[40]^ This values were used to calculate the two‐photon absorption cross‐sections (δ_a_) according to Equation 1:^[41]^

(1) $$\delta {}_{a}=\;\frac{\sqrt{ln2}\cdot N\pi^{\left(\frac{5}{2}\right)}\alpha a_{0}^{5}\omega^{2}}{c\Gamma }\;•\;\left\langle \delta_{2P-ts}\right\rangle$$

in which α is the fine structure constant, a_0_ is the Bohr radius, ω is the excitation energy and c the speed of light in vacuum. Further, the parameters *N*=4 and *Γ*=0.05 eV were used. The results for these computations are shown in Table 1. The excited state potential energy surfaces of **DMA‐** and **Az‐NDBF‐OH** were analyzed in vacuum and solution. Vertical excitation energies, excitonic properties and the relaxed dipole moments of the three energetically lowest singlet excited states were computed using TD‐CAM‐B3LYP/def2‐svp employing the Tamm–Dancoff approximation (TDA). The same procedure was also carried out using a polarizable continuum model (PCM) for the example solvents toluene and methanol. Because we are using Gaussian 16 for a downstream task due to its advanced PCM capabilities, we carried out the optimizations and TDA computations also in this program. The computational results and further details are shown in Tables S5–S8 in the Supporting Information. The detachment and attachment densities for the energetically lowest singlet excited states (CT and LE) of **DMA‐NDBF‐OH** were also obtained using the vacuum minimum geometry and TDA‐CAM‐B3LYP/def2‐svp.

## Conflict of interest

The authors declare no conflict of interest.

## Supporting information

As a service to our authors and readers, this journal provides supporting information supplied by the authors. Such materials are peer reviewed and may be re‐organized for online delivery, but are not copy‐edited or typeset. Technical support issues arising from supporting information (other than missing files) should be addressed to the authors.

Supplementary
