# Supplementary material for: Selective Modification for Red‐Shifted Excitability: A Small Change in Structure, a Huge Change in Photochemistry
Source: Chemistry. 2020 Dec 23;27(6):2212–8. doi: 10.1002/chem.202003672 (PMC7898321; doi:10.1002/chem.202003672)

Chemistry–A European Journal

Supporting Information

Selective Modification for Red-Shifted Excitability: A Small Change in Structure, a Huge Change in Photochemistry

Yvonne Becker,[a] Sina Roth,[b] Maximilian Scheurer,[c] Andreas Jakob,[a] Daniel A. Gacek,[d] Peter J. Walla,[d] Andreas Dreuw,*[c] Josef Wachtveitl,*[b] and Alexander Heckel*[a]

Supporting Information

Table of contents:

1. Synthesis………..1
2. Buffer list..10
3. RP-HPLC gradients and chromatograms...10
4. Absorption/emission additional data...13
5. Two-photon-induced-fluorescence (TPiF) setups and data..15
6. Computational methods and data..16
7. References.. 18
8. NMR spectra of small molecules...19
9. Mass spectra of the target compounds (**x-NDBF-Glu**)...45
10. Overview of spectroscopic properties…………………………………………………………………………………….47

1. Synthesis

An overview of the synthesis route from *m*-halogenated phenols to four different NDBF-caged glutamic acids is given in Scheme S1. As written in the Experimental Section in the main text, 3-(azetidine-1-yl)phenol (**2a**) was synthesized according to literature[1] and **DMA-NDBF-OH** as earlier published.[2] Synthesis of the new and unpublished compounds are described in the following section.

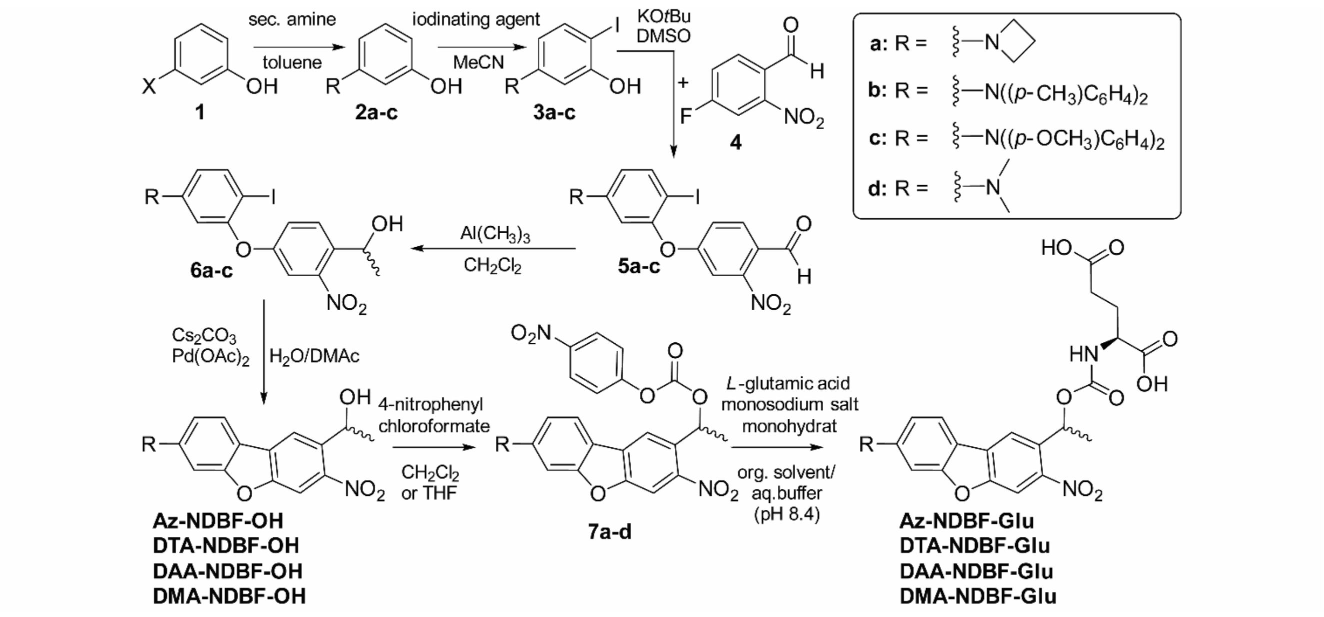

Scheme S1: Syntheses of caged glutamate with different aryl-/alkylamino-NDBF derivatives.

Azetidinyl-NDBF

5-(azetidin-1-yl)-2-iodophenol (3a)

857 mg (3.81 mmol, 1.1 eq) of *N*-iodosuccinimide were added in small portions over 35 min to a suspension of **2a** (514 mg, 3.45 mmol, 1.0 eq) in cooled acetonitrile (MeCN, 12 ml, -10 °C). The black reaction mixture was then stirred for further 16 h at room temperature. After quenching with saturated aq. $Na_2S_2O_3$-solution the aqueous layer was extracted with EtOAc. The combined organic layers were washed with water and brine and dried over Na_2SO_4. The solvent was removed under reduced pressure and the crude product was purified via column chromatography (cyclohexane:EtOAc = 10:1 → 5:1). **3a** was obtained as a white solid.

Yield: 602 mg (64%)

TLC (cyclohexane:EtOAc 5:1): R_f = 0.14

^{1}H-NMR (500 MHz, $CDCl_3$): δ = 7.37 (d, *J* = 8.6 Hz, 1H, H_{ar}), 6.10 (d, *J* = 2.6 Hz, 1H, H_{ar}), 5.85 (dd, *J* = 8.6 Hz, 2.6 Hz, 1H, H_{ar}), 5.16 (s, 1H, –O*H*), 3.85 (t, *J* = 7.3 Hz, 4H, azetidinyl-*H*), 2,35 (p, *J* = 7.3 Hz, 2H, azetidinyl-*H*) ppm.

^{13}C{^{1}H}-NMR (125.8 MHz, $CDCl_3$): δ = 155.3, 154.2, 137.9, 106.8, 98.1, 70.3, 52.4, 16.8 ppm.

ESI-MS: *m/z* calcd. for $C_9H_{11}INO$ $[M+H]^+$ 275.99, found 275.97.

4-(5-(azetidin-1-yl)-2-iodophenoxy)-2-nitrobenzaldehyde (5a)

189 mg KO*t*Bu (1.68 mmol, 1.05 eq) were added to a solution of 439 mg **3a** (1.60 mmol, 1.0 eq) in 30 ml DMSO and stirred for 1 h at room temperature. Afterwards a solution of 296 mg (1.76 mmol, 1.1 eq) 4-fluoro-2-nitrobenzaldehyde in 10 ml DMSO was added dropwise over 10 min. The mixture was stirred further 17 h and then concentrated under reduced pressure. The residue was dissolved in EtOAc and washed with water and brine. The aqueous layer was extracted with EtOAc again and the combined organic layers were dried over Na_2SO_4. The crude product was purified via column chromatography (cyclo-hexane:EtOAc = 10:1) to give a red oil and after coevaporation from CH_2Cl_2 a red solid.

Yield: 580 mg (86%)

TLC (cyclohexane:EtOAc 5:1): R_f = 0.42

^{1}H-NMR (500 MHz, $CDCl_3$): δ = 10.32 (d, *J* = 0.6 Hz, 1H, –C*H*O), 7.97 (d, *J* = 8.6 Hz, 1H, H_{ar}), 7.61 (d, *J* = 8.4 Hz, 1H, H_{ar}), 7.48 (d, *J* = 2.5 Hz, 1H, H_{ar}), 7.20 (ddd, *J* = 8.7 Hz, 2.5 Hz, 0.6 Hz, 1H, H_{ar}), 6.21 – 6.10 (m, 2H, H_{ar}), 3.88 (t, *J* = 7.3 Hz, 4H, azetidinyl-*H*), 2.40 (p, *J* = 7.3 Hz, 2H, azetidinyl-*H*) ppm.

^{13}C{^{1}H}-NMR (125.8 MHz, $CDCl_3$): δ = 187.1 (–*C*HO), 162.0, 154.1, 153.9, 151.7, 140.2, 131.8, 124.8, 121.1, 112.2, 111.6, 104.6, 72.4, 52.3, 16.8 ppm.

MALDI-HRMS: *m/z* calcd. for $C_{16}H_{14}IN_2O_4$ $[M+H]^+$ 424.99928, found 424.99801 (Δm = 0.00127, error 3.0 ppm).

1-(4-(5-(azetidin-1-yl)-2-iodophenoxy)-2-nitrophenyl)ethan-1-ol (6a)

448 mg (1.06 mmol, 1.0 eq.) of **5a** were dissolved in 12 ml CH_2Cl_2 and cooled down to 0 °C before 1.1 ml (2.2 mmol, 2.1 eq, 2 M in hexane) $AlMe_3$ were added dropwise to the yellow solution over 10 min. The (now orange) mixture was stirred 10 min at 0 °C and then allowed to heat up to room temperature. The reaction was quenched by adding 20 ml 1 M NaOH and the organic layer was washed with water and brine. The aqueous layer was extracted with CH_2Cl_2 and the combined organic layers concentrated under vacuum. After being combined with another crude reaction mixture (110 mg **5a**, 0.26 ml $AlMe_3$), the product was purified via column chromatography (cyclohexane:EtOAc = 3:1) and obtained as yellow solid.

Yield: 465 mg (80% overall)

TLC (cyclohexane:EtOAc 5:1): R_f = 0.28

^{1}H-NMR (500 MHz, DMSO-d_6): δ = 7.77 (d, *J* = 8.7 Hz, 1H, H_{ar}), 7.63 (d, *J* = 8.5 Hz, 1H, H_{ar}), 7.28 (d, *J* = 2.6 Hz, 1H, H_{ar}), 7.20 (dd, *J* = 8.7 Hz, 2.6 Hz, 1H, H_{ar}), 6.24 – 6.15 (m, 2H, H_{ar}), 5.47 (d, *J* = 4.3 Hz, 1H, –O*H*), 5.05 (qd, *J* = 6.3 Hz, 4.2 Hz, 1H, COH-C*H*-CH_3), 3.79 (t, *J* = 7.3 Hz, 4H, azetidinyl-*H*), 2.28 (p, *J* = 7.1 Hz, 2H, azetidinyl-*H*), 1.36 (d, *J* = 6.3 Hz, 3H, CH_3) ppm.

^{13}C{^{1}H}-NMR (125.8 MHz, DMSO-d_6): δ = 156.0, 154.3, 153.7, 148.0, 139.4, 135.6, 129.5, 121.1, 111.0, 110.8, 104.4, 73.1, 63.6, 51.9, 25.1, 16.1 ppm.

MALDI-HRMS: *m/z* calcd. for $C_{17}H_{17}IN_2O_4$ $[M\cdot]^+$ 440.02275, found 440.02224 (Δm = 0.00051, error 1.2 ppm). *m/z* calcd. for $C_{17}H_{17}IN_2O_4Na$ $[M+Na]^+$ 463.01252, found 463.01359 (Δm = 0.00107, error 2.3 ppm).

1-(7-(azetidin-1-yl)-3-nitrodibenzo[*b*,*d*]furan-2-yl)ethan-1-ol (Az-NDBF-OH)

29 mg (0.13 mmol, 0.20 eq) $Pd(OAc)_2$ and 417 mg (1.28 mmol, 2.0 eq) Cs_2CO_3 were added to a solution of 282 mg (0.64 mmol, 1.0 eq) **6a** dissolved in 25 ml degassed *N*-dimethylacetamide (DMAc) plus 0.3 ml degassed H_2O. The reaction mixture was stirred for 64 h at 80 °C. Then the mixture was filtered over celite, washed with EtOAc and dried under reduced pressure. The dark-red solid residue was recrystallised from cyclohexane to obtain the closed ring-form **Az-NDBF-OH** as a red solid.

Yield: 56 mg (28%)

TLC (cyclohexane:EtOAc 5:1): R_f = 0.28, (cyclohexane:CH_2Cl_2 1:2): R_f = 0.40

^{1}H-NMR (500 MHz, DMSO-d$_6$): δ = 8.31 (s, 1H, H_{ar}), 8.18 (s, 1H, H_{ar}), 8.04 (d, *J* = 8.5 Hz, 1H, H_{ar}), 6.64 (d, *J* = 1.9 Hz, 1H, H_{ar}), 6.52 (dd, *J* = 8.5 Hz, 2.0 Hz, 1H, H_{ar}), 5.55 (d, *J* = 4.1 Hz, 1H, –O*H*), 5.30 (qd, *J* = 6.2 Hz, 4.2 Hz, 1H, COH-C*H*-CH_3), 3.96 (t, *J* = 7.3 Hz, 4H, azetidinyl-*H*), 2.37 (p, *J* = 7.5 Hz, 2H, azetidinyl-*H*), 1.45 (d, *J* = 6.2 Hz, 3H, CH_3) ppm.

^{13}C{^{1}H}-NMR (125.8 MHz, DMSO-d$_6$): δ = 160.0, 153.5, 152.4, 143.6, 138.5, 129.6, 122.8, 117.3, 111.3, 108.6, 107.0, 92.5, 64.2, 51.9, 25.5, 16.1 ppm.

MALDI-HRMS: *m/z* calcd. for $C_{17}H_{16}N_2O_4$ $[M\cdot]^+$ 312.11046, found 312.10980 (Δm = 0.00066, error 2.1 ppm).

1-(7-(azetidin-1-yl)-3-nitrodibenzo[*b*,*d*]furan-2-yl)ethyl (4-nitrophenyl) carbonate (7a)

58 mg (0.19 mmol, 1.0 eq) **Az-NDBF-OH** were dissolved in 6 ml THF in a microwave reaction vial. 45 mg (0.22 mmol, 1.2 eq) 4-nitrophenyl chloroformate and 34 mg (0.28 mmol, 1.5 eq) 4-dimethylaminopyridine (DMAP) were added and the vial closed. The reaction mixture was stirred for 25 min at 50 °C in the microwave system and then diluted with EtOAc and brine. After extraction with EtOAc the combined organic layer was dried over Na_2SO_4 and concentrated under reduced pressure to give a dark-red solid, which was purified via preparative and automated flash chromatography (cyclohexane/EtOAc gradient).

Yield: 60 mg (66%)

TLC (cyclohexane:EtOAc 5:1): R_f = 0.32

^{1}H-NMR (250 MHz, DMSO-d$_6$): δ = 8.59 (s, 1H, H_{ar}), 8.40 (d, *J* = 8.3 Hz, 1H, H_{ar}), 8.33(s, 1H, H_{ar}), 8.27 (d, *J* = 9.0 Hz, 2H, H_{ar}), 8.08 (s, 1H, H_{ar}), 7.66 (dd, *J* = 8.2 Hz, 1.0 Hz, 1H, H_{ar}), 7.49 (d, *J* = 8.8 Hz, 2H, H_{ar}), 5.33-5.20 (m (q), 1H, C*H*), 4.08-3.95 (m, 2H, azetidinyl-*H*), 3.75 (t, *J* = 6.4 Hz, 2H, azetidinyl-*H*), 2.06 (quint, *J* = 6.6 Hz, 2H, azetidinyl-*H*), 1.48 (d, *J* = 6.2 Hz, 3H, CH_3) ppm.

((1-(7-(azetidin-1-yl)-3-nitrodibenzo[*b*,*d*]furan-2-yl)ethoxy)carbonyl)-*L*-glutamic acid (Az-NDBF-Glu)

To a solution of crude **7a** (22 mg, 46.1 µmol, 1.0 eq) in 4 ml 3:1 (v/v) acetonitrile and THF in a microwave reaction vial were added 11.2 mg (59.9 µmol, 1.3 eq) *L*-glutamic acid monosodium salt monohydrate pre-dissolved in 800 µL aqueous buffer A (see section 2; pH 8.5). The microwave reaction was perfomed at 50 °C for 7 h. The solvent was removed under reduced pressure. The solid residue was washed with acetone (to remove the leftovers from reaction **7a**). The unsoluble solid is a mixture of glutamate and product **Az-NDBF-Glu**, due to NMR analytic and ninhydrin staining on TLC plates. The crude product was purified via preparative RP-HPLC (see section 3, gradient A).

Yield: 3.86 mg (17%)

TLC (cyclohexane:EtOAc 5:1): R_f = 0

RP TLC (MeCN:H_2O+0.1%TFA 1:1): R_f = 0.49

^{1}H-NMR (600 MHz, D_2O/CD_3CN): δ = 8.63 (d, *J* = 9.6 Hz, 1H, H_{ar}), 8.56 (s, 1H, H_{ar}), 8.42 (s, 1H, H_{ar}), 7.39 (d, *J* = 6.8 Hz, 1H, N*H*), 7.27 (t, *J* = 7.4 Hz, 1H, H_{ar}), 6.67 (dd, J = 10.5 Hz, 5.5 Hz, 1H, H_{ar}), 4.59 (t, *J* = 8.9 Hz, 4H, azetidinyl-*H*), 4.55-4.49 (m (q), 1H, C*H*), 2.93-2.83 (m (dt), 2H, azetidinyl-*H*), 2.72 (t, *J* = 7.7 Hz, 1H Glu-C*H*), 2.59-2.46 (m, 1H, Glu-CH_2), 2.37-2.27 (m, 1H, Glu-CH_2), 2.08 (t, *J* = 7.0 Hz, 3H, CH_3) ppm.

^{13}C{^{1}H}-NMR (125.8 MHz, DMSO-d$_6$): δ = 173.6, 173.4, 160.3, 158.2, 155.4, 153.6, 152.7, 143.6, 134.0, 129.9, 126.2, 122.7, 115.8, 111.0, 108.7, 107.5, 92.4, 67.9, 52.4, 30.0, 26.1, 22.3, 16.1 ppm.

MALDI-HRMS: *m/z* calcd. for $C_{23}H_{23}N_3O_9$ $[M\cdot]^+$ 485.14288, found 485.14229 (Δm = 0.00059, error 1.2 ppm). *m/z* calcd. for $C_{23}H_{23}N_3O_9Na$ $[M+Na]^+$ 508.13265, found 508.13174 (Δm = 0.00091, error 1.8 ppm).

Aryl-NDBFs (DTA & DAA-NDBF)

3-(di-*p*-tolylamino)phenol (2b)

8.10 ml (76.03 mmol, 1.0 eq) 3-bromophenol, 15.0 g (76.03 mmol, 1.0 eq) di-*p*-tolylamine, 14.61 g (152.07 mmol, 2.0 eq) sodium *tert*-butoxide, 1.68 ml (3.82 mmol, 0.4 eq) tri-*tert*-butylphosphine and 0.50 g (2.23 mmol, 0.05 eq) palladium(II) acetate ($Pd(OAc)_2$) were dissolved in 50 ml degassed toluene and stirred and heated at 130 °C for 24 h. Afterwards, the reaction mixture was diluted with EtOAc and washed with water. The combined organic layers were dried over $MgSO_4$ and concentrated under vacuum. The crude product was purified via column chromatography (cyclohexane:EtOAc = 6:1) to give a brown oil.

Yield: 7.0 g (32%)

^{1}H-NMR (600 MHz, DMSO-d_6): δ = 9.21 (s, 1H, O*H*), 7.08 (d, *J* = 8.3 Hz, 4H, H_{ar}), 7.02–6.96 (m, 1H, H_{ar}), 6.89 (d, *J* = 8.4 Hz, 4H, H_{ar}), 6.37-6.29 (m, 3H, H_{ar}), 2.25 (s, 6H, CH_3) ppm.

^{13}C{^{1}H}-NMR (151 MHz, DMSO-d_6): δ = 158.1, 148.9, 144.9, 132.0, 129.9, 129.8, 124.3, 112.9, 109.1, 109.0, 20.3 ppm.

MALDI-HRMS: *m/z* calcd. for $C_{20}H_{19}NO$ $[M\cdot]^+$ 289.14612, found 289.14608 (Δm = 0.00004, error 0.14 ppm).

3-(bis(4-methoxyphenyl)amino)phenol (2c)

2.65 ml (19.63 mmol, 1.0 eq) 3-iodophenol, 4.50 g (19.63 mmol, 1.0 eq) bis(4-methoxyphenyl)amine, 5.66 g (58.88 mmol, 3.0 eq) sodium *tert*-butoxide, 0.95 ml (2.16 mmol, 0.11 eq) tri-*tert*-butylphosphine and 0.22 g (0.98 mmol, 0.05 eq) palladium(II) acetate ($Pd(OAc)_2$) were dissolved in 50 ml degassed toluene and stirred and heated at 130 °C for 4 days. Afterwards, the reaction mixture was diluted with EtOAc and washed with water. The combined organic layers were dried over $MgSO_4$ and concentrated under vacuum. The crude product was purified via column chromatography (cyclohexane:EtOAc = 5:1) to give a brown oil.

Yield: 5.1 g (80%)

^{1}H-NMR (500 MHz, DMSO-d_6): δ = 9.13 (s, 1H, O*H*), 7.00–6.97 (m, 4H, H_{ar}), 6.93 (t, *J* = 8.1 Hz, 1H, H_{ar}), 6.90–6.87 (m, 4H, H_{ar}), 6.25–6.23 (m, 1H, H_{ar}), 6.20–6.18 (m, 2H, H_{ar}), 3.72 (s, 6H, O-CH_3) ppm.

^{13}C{^{1}H}-NMR (125.8 MHz, DMSO-d_6): δ = 158.0, 155.6, 149.7, 140.4, 129.6, 126.7, 114.8, 110.6, 107.5, 106.6, 55.2 ppm.

MALDI-HRMS: *m/z* calcd. for $C_{20}H_{19}NO_3$ $[M\cdot]^+$ 321.13594, found 321.13680 (Δm = 0.00086, error 2.7 ppm).

General procedure for 5-(di-*p*-tolylamino)-2-iodophenol (3b)
& **5-(bis(4-methoxyphenyl)amino)-2-iodophenol (3c)**

2.0 g (6.92 mmol, 1.0 eq) 3-(di-*p*-tolylamino)phenol (**2b**) were dissolved in 40 ml MeCN and cooled to -15 °C before 1.87 g (8.30 mmol, 1.2 eq) *N*-iodosuccinimide were added in small portions. The reaction mixture was then stirred overnight at room temperature. After quenching with saturated aq. $Na_2S_2O_3$-solution the aqueous layer was extracted with EtOAc. The combined organic layers were washed with water and the solvent removed under reduced pressure. To obtain **3b** as a solid, the crude product was purified via column chromatography (cyclohexane:EtOAc = 5:1).

3c was prepared in the same way using 2.4 g (7.4 mmol, 1.0 eq) 3-(bis(4-methoxyphenyl)amino)phenol (**2c**).

3b:

Yield: 1.8 g (63%)

^{1}H-NMR (500 MHz, DMSO-d_6): δ = 10.02 (s, 1H, O*H*), 7.42 (d, *J* = 8.6 Hz, 1H, H_{ar}), 7.11 (d, J = 8.2 Hz, 4H, H_{ar}), 6.92 (d, *J* = 8.3 Hz, 4H, H_{ar}), 6.47 (d, *J* = 2.6 Hz, 1H, H_{ar}), 6.14 (dd, *J* = 8.6 Hz, 2.6 Hz, 1H, H_{ar}), 2.26 (s, 6H, CH_3) ppm.

^{13}C{^{1}H}-NMR (125.8 MHz, DMSO-d_6): δ = 157.0, 149.3, 144.4, 138.6, 132.7, 130.1, 124.9, 114.4, 107.8, 74.1, 20.4 ppm.

MALDI-HRMS: *m/z* calcd. for $C_{20}H_{18}INO$ [M·]$^+$ 415.04276, found 415.04229 (Δm = 0.00047, error 1.1 ppm).

3c:

Yield: 2.7 g (81%)

^{1}H-NMR (500 MHz, DMSO-d$_6$): δ = 9.93 (s, 1H, O*H*), 7.35 (d, *J* = 8.7 Hz, 1H, H_{ar}), 7.04–7.01 (m, 4H, H_{ar}), 6.92–6.89 (m, 4H, H_{ar}), 6.36 (d, *J* = 2.6 Hz, 1H, H_{ar}), 6.02 (dd, *J* = 8.6 Hz, 2.6 Hz, 1H, H_{ar}), 3.73 (s, 6H, O-CH_3) ppm.

^{13}C{^{1}H}-NMR (125.8 MHz, DMSO-d$_6$): δ = 156.9, 156.0, 150.1, 139.7, 138.4, 127.2, 114.9, 112.3, 105.6, 72.1, 55.3 ppm.

MALDI-HRMS: *m/z* calcd. for $C_{20}H_{18}INO_3$ [M·]$^+$ 447.03259, found 447.03247 (Δm = 0.00012, error 0.27 ppm).

General procedure for 4-(5-(di-*p*-tolylamino)-2-iodophenoxy)-2-nitrobenzaldehyde (5b) & 4-(5-(bis(4-methoxyphenyl)amino)-2-iodophenoxy)-2-nitrobenzaldehyde (5c)

0.87 g (2.09 mmol, 1.0 eq) 5-(di-*p*-tolylamino)-2-iodophenol (**3b**) and 0.25 g (2.20 mmol, 1.05 eq) potassium *tert*-butoxide were dissolved in 25 ml DMSO and stirred at room temperature for 25 min. Then 0.39 g (2.30 mmol, 1.10 eq) 4-fluoro-2-nitrobenzaldehyde, predissolved in 5 ml DMSO, were added dropwise. The reaction mixture was stirred for further 24 h. DMSO was removed under vacuum. The residue was dissolved in EtOAc, washed with water and the combined organic layers were dried over $MgSO_4$. The crude product was purified via column chromatography (cyclohexane:EtOAc = 10:1) to give **5b** as an orange-colored solid.

5c was prepared in the same way using 0.40 g (0.89 mmol, 1.0 eq) 5-(bis(4-methoxyphenyl)amino)-2-iodophenol (**3c**).

5b:

Yield: 0.94 g (80%)

^{1}H-NMR (500 MHz, DMSO-d$_6$): δ = 10.07 (s, 1H, C*H*O), 7.96 (d, *J* = 8.6 Hz, 1H, H_{ar}), 7.73 (d, *J* = 8.8 Hz, 1H, H_{ar}), 7.58 (s, 1H, H_{ar}), 7.31 (dd, *J* = 8.6, 2.3 Hz, 1H, H_{ar}), 7.13 (d, *J* = 8.2 Hz, 4H, H_{ar}), 7.01 (d, *J* = 8.3 Hz, 4H, H_{ar}), 6.65 (d, *J* = 2.5 Hz, 1H, H_{ar}), 6.58 (dd, *J* = 8.7, 2.6 Hz, 1H, H_{ar}), 2.25 (s, 6H, CH_3) ppm.

^{13}C{^{1}H}-NMR (125.8 MHz, DMSO-d$_6$): δ = 188.3, 160.8, 153.5, 150.9, 150.1, 143.6, 140.1, 133.7, 132.9, 130.3, 125.2, 123.8, 120.0, 119.8, 113.2, 111.8, 78.1, 20.4 ppm.

MALDI-HRMS: *m/z* calcd. for $C_{27}H_{21}IN_2O_4$ [M·]$^+$ 564.05405, found 564.05264 (Δm = 0.00141, error 2.5 ppm).

5c:

Yield: 0.48 g (90%)

^{1}H-NMR (600 MHz, DMSO-d$_6$): δ = 10.07 (s, 1H, C*H*O), 7.96 (d, *J* = 8.6 Hz, 1H, H_{ar}), 7.68 (d, *J* = 8.8 Hz, 1H, H_{ar}), 7.57 (d, *J* = 2.4 Hz, 1H, H_{ar}), 7.29 (dd, *J* = 8.6 Hz, 2.4 Hz, 1H, H_{ar}), 7.15–7.08 (m, 4H, H_{ar}), 6.93–6.88 (m, 4H, H_{ar}), 6.51 (d, *J* = 2.7 Hz, 1H, H_{ar}), 6.47 (dd, *J* = 8.8 Hz, 2.7 Hz, 1H, H_{ar}), 3.72 (s, 6H, O-CH_3) ppm.

^{13}C{^{1}H}-NMR (151 MHz, DMSO-d$_6$): δ = 188.2, 160.8, 156.5, 153.4, 150.8, 139.9, 138.8, 132.8, 127.4, 123.8, 120.0, 115.1, 111.7, 110.8, 75.9, 55.2 ppm.

MALDI-HRMS: *m/z* calcd. for $C_{27}H_{21}IN_2O_6$ 596.04388, found 596.04391 (Δm = 0.00003, error 0.05 ppm).

General procedure for 1-(4-(5-(di-*p*-tolylamino)-2-iodophenoxy)-2-nitrophenyl)ethan-1-ol (6b) & 1-(4-(5-(bis(4-methoxyphenyl)amino)-2-iodophenoxy)-2-nitrophenyl)ethan-1-ol (6c)

3.0 g (5.32 mmol, 1.0 eq) of **5b** were dissolved in 50 ml CH_2Cl_2 and cooled down to 0 °C, before 4.0 ml (7.97 mmol, 1.5 eq, 2 M in hexane) $AlMe_3$ were added dropwise. The ice bath was removed and stirring continued for further 20 min at room temperature. The reaction was stopped by the addition of 10 ml 2 M NaOH and the organic layer was washed with water. The aqueous layer was extracted with EtOAc, the combined organic layers dried over $MgSO_4$ and then concentrated under vacuum. To obtain **6b** as a light yellow solid, the crude product was purified via column chromatography (cyclohexane:EtOAc = 10:1).

6c was prepared in the same way using 0.60 g (1.01 mmol, 1.0 eq) 4-(5-(bis(4-methoxyphenyl)amino)-2-iodophenoxy)-2-nitrobenzaldehyde (**5c**). The crude product was purified via column chromatography (cyclohexane:EtOAc = 8:1) to obtain **6c** as a light yellow solid.

6b:

Yield: 2.60 g (84%)

^{1}H-NMR (500 MHz, DMSO-d$_6$): δ = 7.75 (d, *J* = 8.7 Hz, 1H, H_{ar}), 7.69 (d, *J* = 8.6 Hz, 1H, H_{ar}), 7.34 (d, *J* = 2.5 Hz, 1H, H_{ar}), 7.27 (dd, *J* = 8.7, 2.5 Hz, 1H, H_{ar}), 7.10 (d, *J* = 8.1 Hz, 4H, H_{ar}), 6.97 (d, *J* = 8.2 Hz, 4H, H_{ar}), 6.56–6.46 (m, 2H, H_{ar}), 5.47 (d, *J* = 4.2 Hz, 1H, O*H*), 5.08–4.99 (m, 1H, C*H*), 2.24 (s, 6H, CH_3), 1.34 (d, *J* = 6.3 Hz, 3H, CH_3) ppm.

^{13}C{^{1}H}-NMR (125.8 MHz, DMSO-d$_6$): δ = 155.4, 154.7, 149.8, 147.9, 143.6, 139.9, 136.0, 133.6, 130.2, 129.5, 125.1, 121.7, 119.0, 112.3, 111.4, 77.9, 63.5, 25.0, 20.4 ppm.

MALDI-HRMS: *m/z* calcd. for $C_{28}H_{25}IN_2O_4$ [M·]$^+$ 580.08535, found 580.08467 (Δm = 0.00068, error 1.2 ppm).

6c:

Yield: 0.37 g (60%)

^{1}H-NMR (500 MHz, DMSO-d$_6$): δ = δ 7.75 (d, *J* = 8.7 Hz, 1H, H_{ar}), 7.64 (d, *J* = 8.7 Hz, 1H, H_{ar}), 7.34 (d, *J* = 2.6 Hz, 1H, H_{ar}), 7.25 (dd, *J* = 8.7, 2.6 Hz, 1H, H_{ar}), 7.10–7.06 (m, 4H, H_{ar}), 6.90–6.87 (m, 4H, H_{ar}), 6.41–6.38 (dd, *J* = 8.7, 2.7 Hz, 1H, H_{ar}), 6.36 (d, *J* = 2.6 Hz, 1H, H_{ar}), 5.46 (d, *J* = 4.3 Hz, 1H, O*H*), 5.06–5.01 (m, 1H, C*H*), 3.72 (s, 6H, O-CH_3), 1.34 (d, *J* = 6.3 Hz, 3H, CH_3) ppm.

^{13}C{^{1}H}-NMR (125.8 MHz, DMSO-d$_6$): δ = 156.4, 155.4, 154.5, 150.6, 147.9, 139.7, 138.9, 135.9, 129.5, 127.3, 121.6, 116.9, 115.1, 111.3, 110.1, 75.9, 63.5, 55.2, 25.0 ppm.

MALDI-HRMS: *m/z* calcd. for $C_{28}H_{25}IN_2O_6$ [M·]$^+$ 612.07518, found 612.07468 (Δm = 0.00050, error 0.82 ppm).

General procedure for 1-(7-(di-*p*-tolylamino)-3-nitrodibenzo[*b*,*d*]furan-2-yl)ethan-1-ol (DTA-NDBF-OH) & 1-(7-(bis(4-methoxyphenyl)amino)-3-nitrodibenzo[*b*,*d*]furan-2-yl)ethan-1-ol (DAA-NDBF-OH)

190 mg (0.85 mmol, 0.02 eq) Pd(OAc)$_2$ and 2.81 g (8.61 mmol, 2.0 eq) Cs_2CO_3 were added to a solution of 2.50 g (4.31 mmol, 1.0 eq) **6b** dissolved in 50 ml degassed *N*-dimethylacetamide (DMAc). The reaction mixture was stirred for 48 h at 80 °C. Then water and EtOAc were added. After extraction, the combined organic layer was dried over $MgSO_4$ and dried under vacuum. To obtain the closed-ring form **DTA-NDBF-OH** as a copper-colored solid, the crude product was purified via column chromatography (cyclohexane:EtOAc = 6:1).

DAA-NDBF-OH was prepared in the same way using 1.50 g (2.45 mmol, 1.0 eq) 1-(4-(5-(bis(4-methoxyphenyl)amino)-2-iodophenoxy)-2-nitrophenyl)ethan-1-ol (**6c**).

DTA-NDBF-OH:

Yield: 1.50 g (76%)

^{1}H-NMR (500 MHz, DMSO-d$_6$): δ = 8.38 (s, 1H, H_{ar}), 8.18 (s, 1H, H_{ar}), 8.07 (d, *J* = 8.6 Hz, 1H, H_{ar}), 7.18 (d, *J* = 8.2 Hz, 4H, H_{ar}), 7.04 (d, *J* = 8.3 Hz, 4H, H_{ar}), 6.99 (d, *J* = 1.9 Hz, 1H, H_{ar}), 6.89 (dd, *J* = 8.6, 2.0 Hz, 1H, H_{ar}), 5.59 (d, *J* = 4.2 Hz, 1H, O*H*), 5.30–5.26 (m, 1H, C*H*), 2.29 (s, 6H, CH_3), 1.45 (d, *J* = 6.3 Hz, 3H, CH_3) ppm.

^{13}C{^{1}H}-NMR (125.8 MHz, DMSO-d$_6$): δ = 159.1, 153.0, 149.9, 144.7, 144.0, 138.2, 133.8, 130.4, 128.6, 125.5, 122.8, 118.3, 116.9, 114.9, 107.3, 102.0, 64.1, 25.5, 20.5 ppm.

MALDI-HRMS: *m/z* calcd. for $C_{28}H_{24}N_2O_4$ [M·]$^+$ 452.17306, found 452.17192 (Δm = 0.00140, error 2.5 ppm).

DAA-NDBF-OH:

Yield: 1.13 g (95%)

^{1}H-NMR (500 MHz, DMSO-d_6): δ = 8.34 (s, 1H, H_{ar}), 8.16 (s, 1H, H_{ar}), 8.02 (d, J = 8.6 Hz, 1H, H_{ar}), 7.16 (d, J = 8.8 Hz, 4H, H_{ar}), 6.98 (d, J = 8.9 Hz, 4H, H_{ar}), 6.81 (d, J = 1.7 Hz, 1H, H_{ar}), 6.78 (dd, J = 8.7, 1.8 Hz, 1H, H_{ar}), 5.58 (d, J = 4.1 Hz, 1H, O*H*), 5.31–5.26 (m, 1H, C*H*), 3.77 (s, 6H, O-CH_3), 1.44 (d, J = 6.2 Hz, 3H, CH_3) ppm.

$^{13}C\{^{1}H\}$-NMR (125.8 MHz, DMSO-d_6): δ = 159.3, 156.7, 152.9, 150.8, 144.3, 139.1, 138.3, 128.9, 127.8, 122.7, 117.9, 115.2, 114.8, 113.6, 107.2, 99.3, 64.1, 55.3, 25.5 ppm.

MALDI-HRMS: *m/z* calcd. for $C_{28}H_{24}N_2O_6$ [M·]$^+$ 484.16289, found 484.16114 (Δm = 0.00175, error 3.6 ppm).

General procedure for 1-(7-(di-*p*-tolylamino)-3-nitrodibenzo[*b*,*d*]furan-2-yl)ethyl (4-nitrophenyl) carbonate (7b) & 1-(7-(bis(4-methoxyphenyl)amino)-3-nitrodibenzo[*b*,*d*]furan-2-yl)ethyl (4-nitrophenyl) carbonate (7c)

280 mg (0.62 mmol, 1.0 eq) **DTA-NDBF-OH** were dissolved in 20 ml CH_2Cl_2 and 250 mg (1.24 mmol, 2.0 eq) 4-nitrophenyl chloroformate and 0.43 ml (2.48 mmol, 4.0 eq) DIPEA were added. The reaction mixture was stirred for 48 h at room temperature, after half of the time further 2.5 eq DIPEA were added. After the solution was diluted with EtOAc and washed with water, the combined organic layers were dried over $MgSO_4$ and concentrated under vacuum. The residue was purified via column chromatography (cyclohexane:EtOAc = 10:1) to obtain **7b** as a red solid.

7c was prepared in the same way using 200 mg (0.41 mmol, 1.0 eq) 1-(7-(bis(4-methoxyphenyl)amino)-3-nitrodibenzo[*b*,*d*]furan-2-yl)ethan-1-ol (**DAA-NDBF-OH**).

7b:

Yield: 170 mg (37%)

^{1}H-NMR (600 MHz, DMSO-d_6): δ = 8.45 (s, 1H, H_{ar}), 8.31 (s, 1H, H_{ar}), 8.27 (d, J = 9.0 Hz, 2H, H_{ar}), 8.15 (d, J = 8.6 Hz, 1H, H_{ar}), 7.50 (d, J = 9.0 Hz, 2H, H_{ar}), 7.20 (d, J = 8.0 Hz, 4H, H_{ar}), 7.06 (d, J = 8.1 Hz, 4H, H_{ar}), 7.00 (brs, 1H, H_{ar}), 6.94 (d, J = 8.5 Hz, 1H, H_{ar}), 6.32 (q, J = 6.3 Hz, 1H, C*H*), 2.30 (s, 6H, O-CH_3), 1.82 (d, J = 6.4 Hz, 3H, CH_3) ppm.

$^{13}C\{^{1}H\}$-NMR (125.8 MHz, DMSO-d_6): δ = 159.3, 155.1, 153.6, 151.3, 150.3, 145.2, 144.9, 143.9, 134.0, 131.4, 130.4, 129.3, 125.6, 125.4, 123.3, 122.5, 118.6, 117.0, 114.5, 108.0, 101.7, 73.3, 21.7, 20.5 ppm.

MALDI-HRMS: *m/z* calcd. for $C_{35}H_{27}N_3O_8$ [M·]$^+$ 617.17927, gefunden 617.17846 (Δm = 0.00081, error 1.31 ppm).

7c:

Yield: 0.08 g (31%)

^{1}H-NMR (400 MHz, DMSO-d_6): δ = 8.40 (s, 1H, H_{ar}), 8.29 (s, 1H, H_{ar}), 8.27 (d, J = 9.2 Hz, 2H, H_{ar}), 8.10 (d, J = 9.3 Hz, 1H, H_{ar}), 7.50 (d, J = 9.1 Hz, 2H, H_{ar}), 7.19 (d, J = 8.9 Hz, 4H, H_{ar}), 6.99 (d, J = 8.9 Hz, 4H, H_{ar}), 6.82 (dd, J = 6.8, 2.0 Hz, 2H, H_{ar}), 6.33 (q, J = 6.3 Hz, 1H, C*H*), 3.77 (s, 6H, O-CH_3), 1.81 (d, J = 6.4 Hz, 3H, CH_3) ppm.

(After re-crystallization) ^{1}H-NMR (600 MHz, C_6D_6): δ = 7.97 (s, 1H, H_{ar}), 7.81 (s, 1H, H_{ar}), 7.58 (d, J = 9.0 Hz, 2H, H_{ar}), 7.31 (d, J = 8.6 Hz, 1H, H_{ar}), 7.19 (s, 1H, H_{ar}), 7.07–7.03 (m, 5H, H_{ar}), 6.79–6.70 (m, 5H, H_{ar}) 6.68 (d, J = 9.0 Hz, 2H, H_{ar} & C*H*), 3.30 (s, 6H, O-CH_3), 1.64 (d, J = 6.4 Hz, 3H, CH_3) ppm.

$^{13}C\{^{1}H\}$-NMR (151 MHz, C_6D_6): δ = 160.8, 157.6, 155.3, 154.7, 151.9, 151.8, 145.6, 145.4, 140.3, 128.4, 125.2, 122.1, 121.3, 117.0, 116.5, 115.5, 114.6, 108.6, 101.7, 74.2, 55.1, 22.3 ppm.

MALDI-HRMS: *m/z* calcd. for $C_{35}H_{28}N_3O_{10}$ [M+H]$^+$ 650.17638, found 650.17223 (Δm = 0.00415, error 6.4 ppm).

General procedure for ((1-(7-(di-*p*-tolylamino)-3-nitrodibenzo[*b*,*d*]furan-2-yl)ethoxy)carbonyl)-*L*-glutamic acid (DTA-NDBF-Glu) & ((1-(7-(bis(4-methoxyphenyl)amino)-3-nitrodibenzo[*b*,*d*]furan-2-yl)ethoxy)carbonyl)-*L*-glutamic acid (DAA-NDBF-Glu)

In an eppendorf tube 7 mg (11.4 µmol, 1.0 eq) of **7b** were dissolved in 1 ml DMF. 2.75 mg (14.7 µmol, 1.3 eq) *L*-glutamic acid monosodium salt monohydrate, pre-dissolved in 150 µL aqueous buffer B (see section 2; pH 8.4), were added and the reaction mixture was shaked for 2 days at room temperature on a thermoshaker. Afterwards, the solvent was removed under reduced

pressure and the residue redissolved in water/acetonitril 3:1 (v/v). The crude product was purified via preparative RP-HPLC (see section 3, gradient A).

DAA-NDBF-Glu was prepared in the same way using 7 mg (10.8 µmol, 1.0 eq) 1-(7-(bis(4-methoxyphenyl)amino)-3-nitrodibenzo[*b*,*d*]furan-2-yl)ethyl (4-nitrophenyl) carbonate (**7c**).

DTA-NDBF-Glu:

Yield: 5.5 mg (77%)

^{1}H-NMR (600 MHz, D_2O/CD_3CN): δ = 8.59 (d, *J* = 18.2 Hz, 1H, H_{ar}), 8.36 (d, *J* = 7.2 Hz, 1H, H_{ar}), 8.29–8.17 (m, 1H, H_{ar}), 7.51 (t, *J* = 6.5 Hz, 4H, tolyl-H_{ar}), 7.37 (t, *J* = 7.9 Hz, 4H, tolyl-H_{ar}), 7.30–7.26 (m, 2H, H_{ar}), 6.74–6.62 (m, 1H, C*H*), 4.33 (d, *J* = 22.1 Hz, 1H, Glu-C*H*), 2.85-2-74 (m, 1H, Glu-CH_2), 2.69 (brs, 6H, CH_3), 2.64 (t, 1H, Glu-CH_2), 2.39-2.18 (m, 2H, Glu-CH_2), 2.05 (brs, 3H, CH_3) ppm. (N*H* only observed in DMSO-d_6, COO*H* was not detectable whether in D_2O, MeOD nor DMSO-d_6).

^{1}H-NMR (500 MHz, DMSO-d_6): δ = 8.37-8.16 (m, 2H, H_{ar}), 8.12-7.96 (m, 1H, H_{ar}), 7.27-7.13 (m, 4H, tolyl-H_{ar}), 7.12-7.01 (m, 4H, tolyl-H_{ar}), 7.01- 6.80 (m 3H, H_{ar}) & N*H*), 6.11 (s, 1H, C*H*), 3.76 (s, 1H (in H_2O-signal), Glu-C*H*), 2.30 (brs, 6H+2H, CH3 & Glu-CH_2), 1.92-1.67 (m, 2H, Glu-CH_2), 1.60 (brs, 3H, CH_3).

^{13}C{^{1}H}-NMR (125.8 MHz, DMSO-d_6): δ = 174.4, 173.1, 159.2, 154.5, 153.2, 150.1, 144.6, 144.0, 133.9, 130.4, 129.1, 125.6, 123.0, 118.2, 117.0, 114.7, 107.7, 101.8, 67.4, 53.8, 32.3, 28.1, 22.4, 20.5 ppm.

MALDI-HRMS: *m/z* calcd. for $C_{34}H_{31}N_3O_9$ $[M\cdot]^+$ 625.20548, found 625.20511 (Δm = 0.00037, error 0.59 ppm).

DAA-NDBF-Glu:

Yield: 3.0 mg (42%)

^{1}H-NMR (400 MHz, DMSO-d_6): δ = 12.48 (brs, COO*H*), 8.23 (d, J = 19.5 Hz, 1H, H_{ar}), 7.98-7.92 (m, 1H, H_{ar}), 7.68-7.60 (m, 1H, H_{ar}), 7.19 (d, J =7.7 Hz, 4H, anisyl-H_{ar}), 6.99 (d, J = 8.3 Hz, 4H, anisyl-H_{ar}), 6.88-6.69 (m, 2H+1H, H_{ar} & N*H*), 6.14 (q, *J* = 6.0 Hz, 1H, C*H*), 3.77 (s, 6H, CH_3), 2.23-2.03 (m, 2H, Glu-CH_2), 2.00-1.88 (m, 1H, Glu-CH_2), 1.80-1.67 (m, 1H, Glu-CH_2), 1.67-1.53 (m, 3H, CH_3) ppm.

^{13}C{^{1}H}-NMR (151 MHz, CD_3CN): δ = 175.9, 174.7, 158.0, 156.9, 154.7, 145.4, 140.8, 135.0, 130.9, 128.8, 123.2, 116.1, 108.7, 101.0, 70.0, 56.3, 54.1, 42.8, 40.3, 30.7, 27.5, 22.7, 18.7, 17.3 ppm.

MALDI-HRMS: *m/z* calcd. for $C_{34}H_{31}N_3O_{11}$ $[M\cdot]^+$ 657.19531, found 657.19517 (Δm = 0.00014, error 0.21 ppm).

DMA-NDBF

1-(7-(dimethylamino)-3-nitrodibenzo[*b*,*d*]furan-2-yl)ethyl (4-nitrophenyl) carbonate (7d)

300 mg (1.0 mmol, 1.0 eq) **DMA-NDBF-OH**, synthesized according to earlier published procedures[2], were dissolved in 50 ml CH_2Cl_2 and 400 mg (2.0 mmol, 2.0 eq) 4-nitrophenyl chloroformate and 0.9 ml (5.0 mmol, 5.0 eq.) diisopropylethylamine (DIPEA) were added. The reaction mixture was stirred for 48 h, after half of the time further 2.5 eq DIPEA were added. After the solution was diluted with EtOAc and washed with water, the combined organic layers were dried over $MgSO_4$ and concentrated under vacuum. The residue was purified via column chromatography (cyclohexane:EtOAc = 5:1) to obtain **7d** as a red solid.

Yield: 170 mg (37%)

^{1}H-NMR (600 MHz, DMSO-d_6): δ = 8.35 (s, 1H, H_{ar}), 8.28 (dd, *J* = 6.5 Hz, 2.7 Hz, 3H, H_{ar}), 8.12 (d, *J* = 8.5 Hz, 1H, H_{ar}), 7.51 (d, *J* = 9.2 Hz, 2H, H_{ar}), 6.98 – 6.90 (m, 2H, H_{ar}), 6.36 (q, *J* = 6.3 Hz, 1H, C*H*), 3.07 (s, 6H, CH_3), 1.82 (d, *J* = 6.4 Hz, 3H, CH_3) ppm.

^{13}C{^{1}H}-NMR (151 MHz, DMSO-d_6): δ = 160.6, 155.1, 153.0, 152.5, 151.3, 145.2, 143.7, 131.4, 130.1, 125.3, 123.0, 122.5, 117.2, 110.1, 110.0, 107.6, 93.3, 73.3, 40.2, 21.7 ppm.

MALDI-HRMS: *m/z* calcd. for $C_{23}H_{20}N_3O_8$ $[M+H]^+$ 466.12395, found 466.12341 (Δm = 0.00054, error 1.2 ppm).

((1-(7-(dimethylamino)-3-nitrodibenzo[*b*,*d*]furan-2-yl)ethoxy)carbonyl)-*L*-glutamic acid (DMA-NDBF-Glu)

In an eppendorf tube 7 mg (15.5 µmol, 1.0 eq) of **7d** were dissolved in 1 ml DMSO. 3.7 mg (19.8 µmol, 1.3 eq) *L*-glutamic acid monosodium salt monohydrate, pre-dissolved in 150 µL aqueous buffer B (see section 2; pH 8.4), were added and the reaction mixture was shaked for 2 days at room temperature on a thermoshaker. Afterwards, the solvent was removed under reduced pressure and the residue redissolved in water/acetonitril 3:1 (v/v). The crude product was purified via preparative RP-HPLC (see section 3, gradient A).

Yield: 5.5 mg (75%)

^{1}H-NMR (600 MHz, D_2O/CD_3CN): δ = 9.08 (d, *J* = 10.2 Hz, 1H, N*H*), 8.95 (s, 1H, H_{ar}), 8.91 (dd, *J* = 8.5 Hz, 4.8 Hz, 1H, H_{ar}), 8.27 (d, *J* = 2.6 Hz, 1H, H_{ar}), 8.11 (dd, *J* = 11.0 Hz, 4.2 Hz, 1H, H_{ar}), 6.99 (p, *J* = 6.5 Hz, 1H, H_{ar}), 4.85 (ddd, *J* = 17.8 Hz, 9.1 Hz, 4.9 Hz, 1H, C*H*), 3.97 (s, 6H, CH_3), 3.18 (t, *J* = 7.5 Hz, 1H, NH-C*H*-COOH), 3.05 (t, *J* = 7.5 Hz, 1H, Glu-CH_2), 2.95 – 2.76 (m, 1H, Glu-CH_2), 2.76 – 2.74 (m, 3H, CH_3), 2.64 (ddt, *J* = 22.1 Hz, 15.3 Hz, 7.6 Hz, 1H, Glu-CH_2), 2.43 (under CD_3CN signal, 1H, Glu-CH_2) ppm.

^{13}C{^{1}H}-NMR (151 MHz, D_2O/CD_3CN): δ = 175.5, 174.1, 160.6, 159.3, 156.0, 154.2, 145.6, 134.1, 129.0, 123.5, 118.9, 117.3, 115.5, 113.8, 108.1, 69.0, 53.1, 43.8, 29.7, 26.5, 21.7 ppm.

MALDI-HRMS: *m/z* calcd. for $C_{22}H_{23}N_3O_9$ $[M\cdot]^+$ 473.14288, found 473.14193 (Δm = 0.00095, error 2.0 ppm).

NDBF

For comparability reasons, a glutamate-NDBF without amino-moiety (R = H) was also synthesized. **NDBF-OH** synthesis was based on a previous publication by Deiters *et al.* "Improved synthesis of the two-photon caging group 3-nitro-2-ethyldibenzofuran and its application to a caged thymidine phosphoramidite".[3]

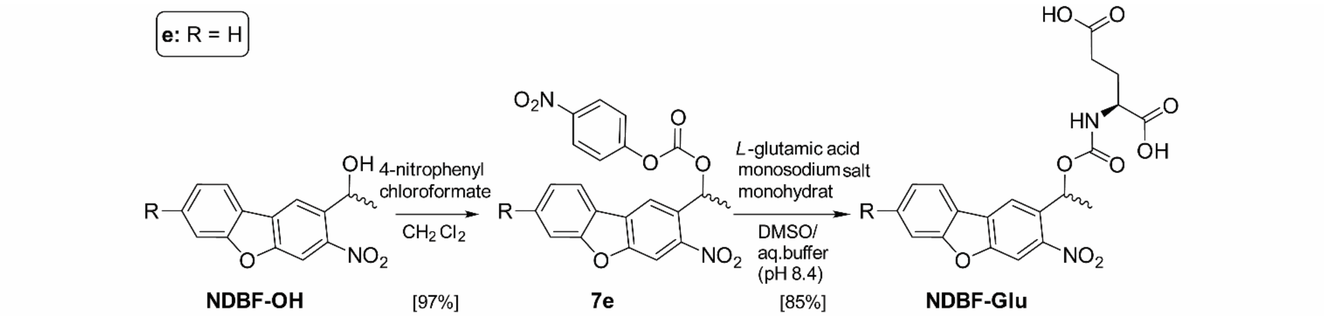

Scheme S2: Synthesis of caged glutamate with NDBF.

1-(3-nitrodibenzo[*b*,*d*]furan-2-yl)ethyl (4-nitrophenyl) carbonate (7e)

300 mg (1.17 mmol, 1.0 eq) **NDBF-OH** were dissolved in 50 ml CH_2Cl_2 and 470 mg (2.33 mmol, 2.0 eq) 4-nitrophenyl chloroformate and 0.75 ml (4.41 mmol, 3.8 eq) DIPEA were added. The reaction mixture was stirred overnight at room temperature. After the solution was diluted with EtOAc and washed with water, the combined organic layers were dried over $MgSO_4$ and concentrated under vacuum. The residue was purified via column chromatography (cyclohexane:EtOAc = 10:1) to obtain **7e** as a white solid.

Yield: 480 mg (97%)

^{1}H-NMR (600 MHz, DMSO-d$_6$): δ = 8.68 (s, 1H, H_{ar}), 8.48 – 8.45 (m, 1H, H_{ar}), 8.42 (d, *J* = 7.8 Hz, 1H, H_{ar}), 8.27 (d, *J* = 9.1 Hz, 2H, H_{ar}), 7.83 (d, *J* = 8.2 Hz, 1H, H_{ar}), 7.69 (t, *J* = 7.8 Hz, 1H, H_{ar}), 7.57 – 7.46 (m, 3H, H_{ar}), 6.30 (q, *J* = 6.4 Hz, 1H, C*H*), 1.85 (d, *J* = 6.5 Hz, 3H, CH_3) ppm.

$^{13}C\{^1H\}$-NMR (151 MHz, DMSO-d_6): δ = 157.6, 155.0, 153.6, 151.3, 146.6, 145.2, 130.7, 130.0, 128.6, 125.3, 124.1, 122.8, 122.5, 121.9, 120.3, 112.1, 108.4, 73.1, 21.6 ppm.

((1-(3-nitrodibenzo[*b*,*d*]furan-2-yl)ethoxy)carbonyl)-*L*-glutamic acid (NDBF-Glu)

In an eppendorf tube 7 mg (16.5 µmol, 1.0 eq) of **7e** were dissolved in 1 ml DMSO. 4 mg (21.5 µmol, 1.3 eq) *L*-glutamic acid monosodium salt monohydrate, pre-dissolved in 150 µL aqueous buffer B (see section 2; pH 8.4), were added and the reaction mixture was shaked for 2 days at room temperature on a thermoshaker. Afterwards, the solvent was removed under reduced pressure and the residue redissolved in water/acetonitril 3:1 (v/v). The crude product was purified via preparative RP-HPLC (see section 3, gradient A).

Yield: 6 mg (85%)

^1H-NMR (600 MHz, D_2O/CD_3CN): δ = 9.48 (d, *J* = 8.2 Hz, 1H, H_{ar}), 9.29 (s, 1H, H_{ar}), 9.25 (d, *J* = 7.7 Hz, 1H, H_{ar}), 8.77 (d, *J* = 8.3 Hz, 1H, H_{ar}), 8.73 (t, *J* = 7.7 Hz, 1H, H_{ar}), 8.58 – 8.56 (m, 1H, H_{ar}), 7.35 – 7.30 (m, 1H, H_{ar}), 5.19 (ddd, *J* = 18.1 Hz, 8.9 Hz, 4.9 Hz, 1H, C*H*), 3.50 (t, *J* = 7.5 Hz, 1H, CH_2), 3.38 (t, *J* = 7.5 Hz, 1H, CH_2), 3.23 – 3.11 (m, 1H, CH_2), 3.02 – 2.92 (m, 1H, CH_2), 2.77 (t, *J* = 7.4 Hz, 3H, CH_3) ppm.

$^{13}C\{^1H\}$-NMR (151 MHz, D_2O/CD_3CN): δ = 176.0, 174.8, 159.2, 156.9, 154.8, 147.3, 134.4, 130.8, 130.1, 125.0, 123.3, 120.6, 120.5, 113.0, 109.2, 69.9, 54.1, 30.7, 27.4, 22.7 ppm.

MALDI-HRMS: *m/z* calcd. for $C_{20}H_{18}N_2O_9K$ $[M+K]^+$ 469.06439, found 469.06351 (Δm = 0.00088, error 1.9 ppm).

2. Buffer list

A: Borax buffer for synthesis of caged glutamates

100 mM $Na_2B_4O_7$ in 1 l distilled H_2O.

Final pH (8.5) adjusted with boric acid (H_3BO_3) and/or NaOH.

B: Carbonate buffer for synthesis of caged glutamates

10 ml 1x PBS + 0.75 ml 0.2 M $NaHCO_3$ solution (pH 9.0).

Final pH (8.4) adjusted with HCl.

C: 10x Phosphate buffered saline (PBS)

1.37 M NaCl + 27 mM KCl + 14 mM KH_2PO_4 + 100 mM Na_2HPO_4 in 1 l distilled H_2O.

Final pH (7.4) adjusted with HCl.

3. RP-HPLC chromatograms

Table S2: Gradient A for prep. reversed phase flash chromatography (puriFlash® XS 420 ULTRA system) with a "Chromabond® Flash" column (RS15 C_{18} ec, 15-40 µm, volume: 29 ml) from *Macherey-Nagel.*

min.	MeCN [%]	H_2O + 0.1% TFA [%]	Flow [ml/min]
0	5	95	20
3.22	5	95	20
28.34	100	0	20
31.56	100	0	20
35.17	5	95	20
36.58	5	95	20

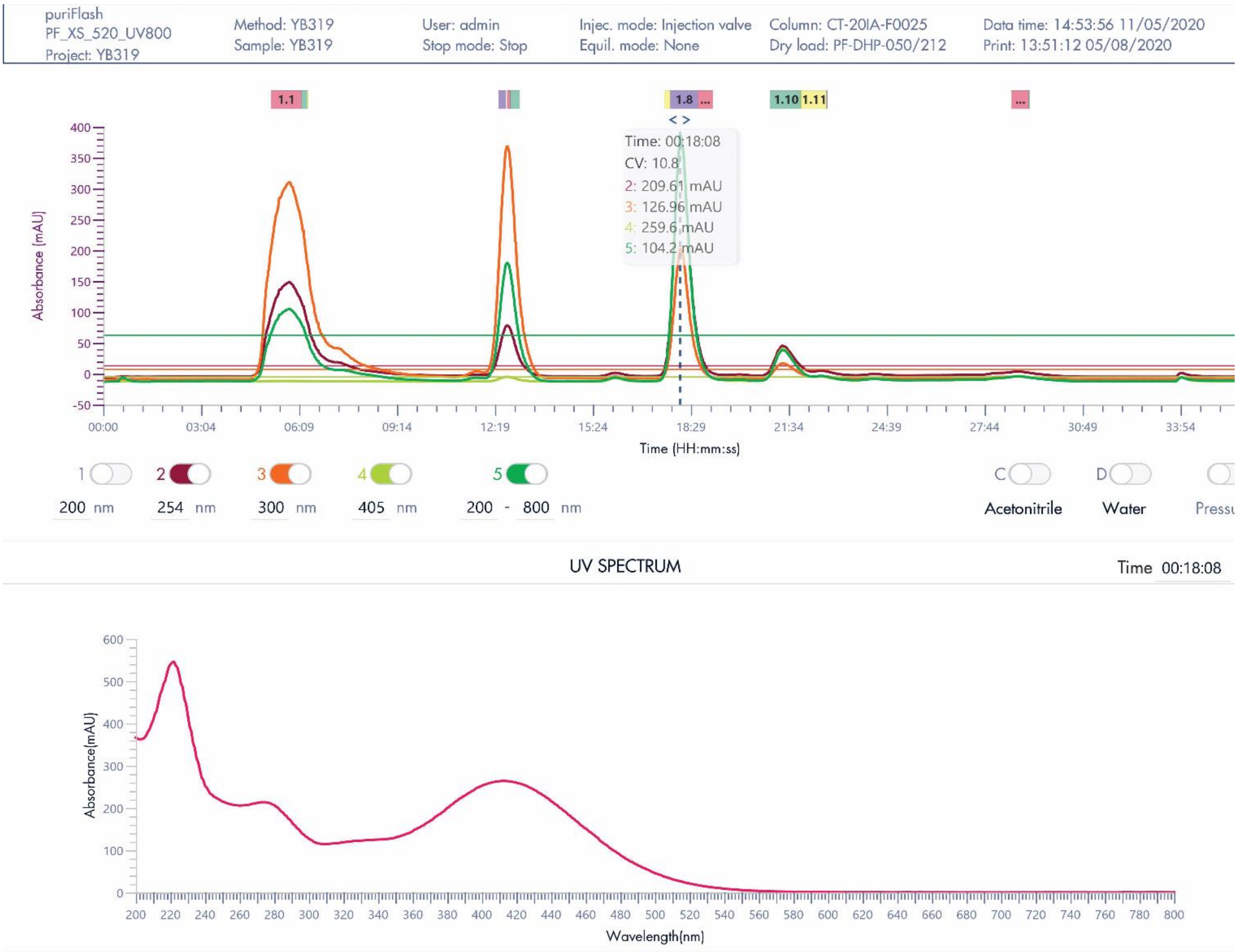

Figure S1: Screen view (*Interchim* software) of an examplary prep. flash purification of **Az-NDBF-Glu**. For fraction collection, the absorbance between 200-800 nm was monitored (dark green line). Fraction **1.8** (retention time Rt = 18.08 min.) contained the purified product (see UV spectrum).

Table S3 & S4: Gradient B (left) for analytical HPLC runs of the caged glutamates (see Figure S2) and **C** (right) for analytical HPLC runs of photolysis/hydrolysis (see Figure S3). Columns: MultoKrom® 100-5 C_{18} (*CS Chromatographie*) and Jupiter® Proteo 90-4 (*Phenomenex*), 4.6 x 250 mm.

min	MeCN [%]	H_2O + 0.1% TFA [%]	Flow [ml/min]
0	5	95	1
1	5	95	1
60	100	0	1
65	100	0	1

min	MeCN [%]	H_2O + 0.1% TFA [%]	Flow [ml/min]
0	5	95	1
2	5	95	1
28	100	0	1
35	100	0	1

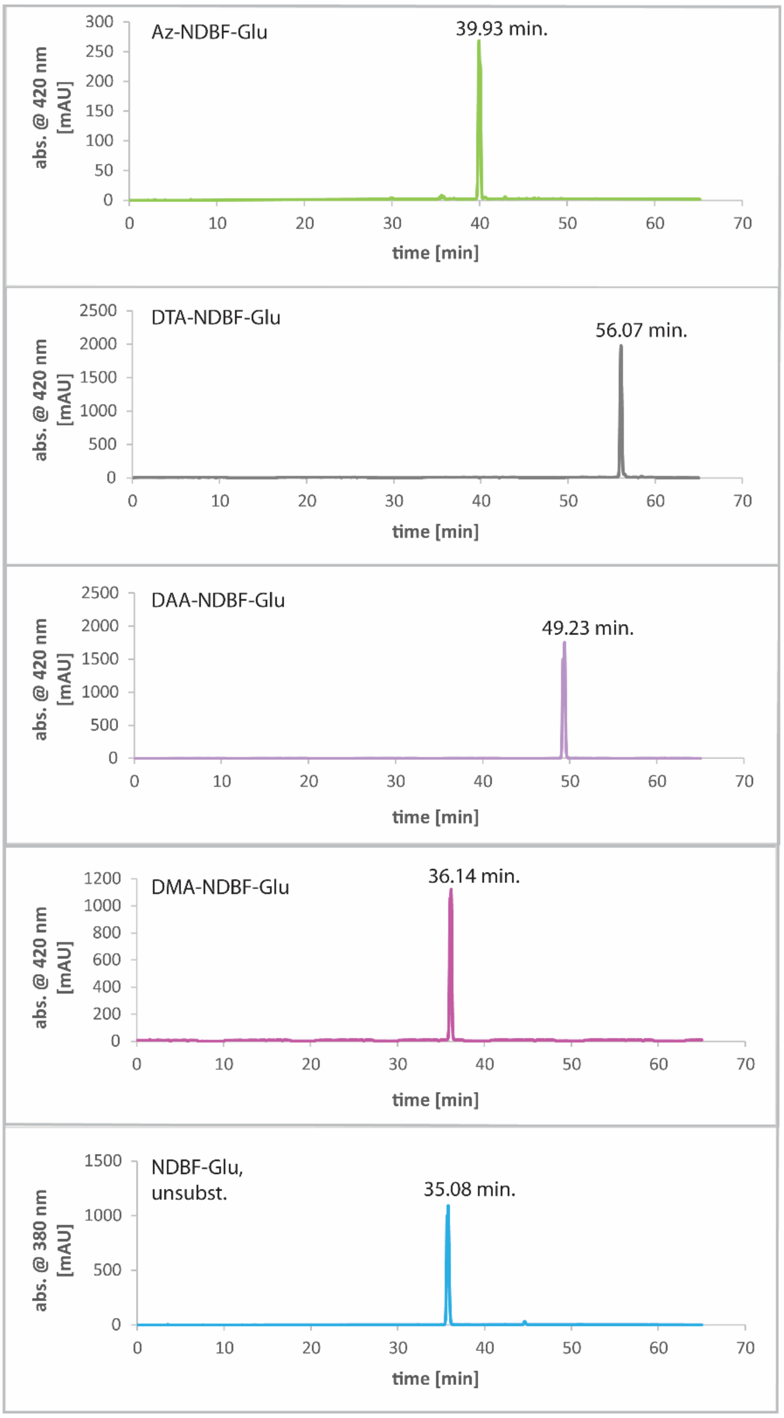

Figure S2: Analytical RP-HPLC chromatograms of **Az-**, **DTA-**, **DAA-** and **DMA-NDBF-Glu** in comparison with unsubstituted **NDBF-Glu (**top to bottom). 320, 380 and 420 nm were chosen as monitoring wavelengths.

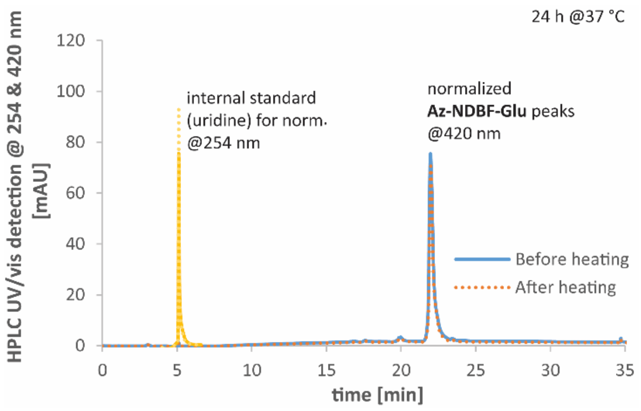

Figure S3 The hydrolysis stability of our green-PPG **Az-NDBF-Glu** was tested in aqueous solution (1x PBS, see section 2) for 24 h at 37 °C. RP-HPLC chromatography with uridine as internal standard for quantification (detected @254 nm, yellow signal) was performed before and after heating. The compound signal at Rt = 21.9 min (@ 420 nm) decreased by 7%.

4. Absorption/emission additional data

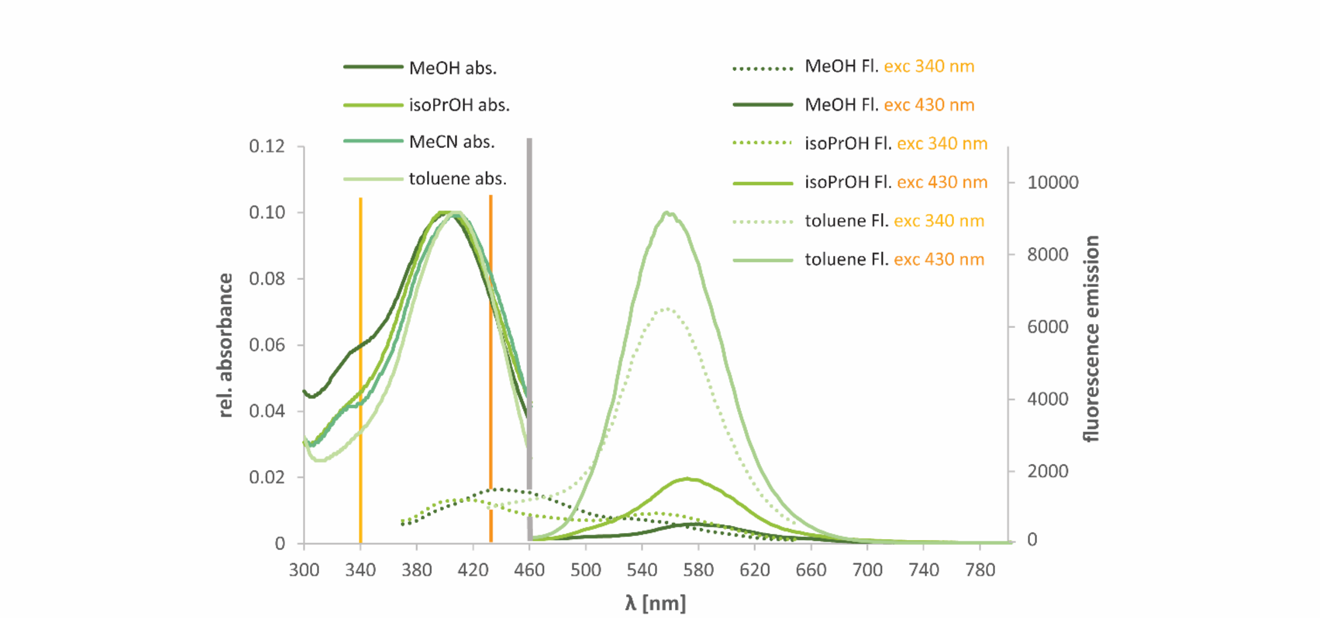

Figure S4 Absorbance and fluorescence emission spectra of **Az-NDBF-OH** in various solvents (MeOH = methanol, isoPrOH = isopropanol, MeCN = acetonitrile and toluene). The values of λ(abs.max.) are between 402 (MeOH) and 407 nm (toluene).The fluorescence emission was detected between 370-650 nm or 460-800 nm, depending on the excitation wavelength (340 or 430 nm, yellow strokes). The highest λ(em.max.) can be seen in toluene at 558 nm.

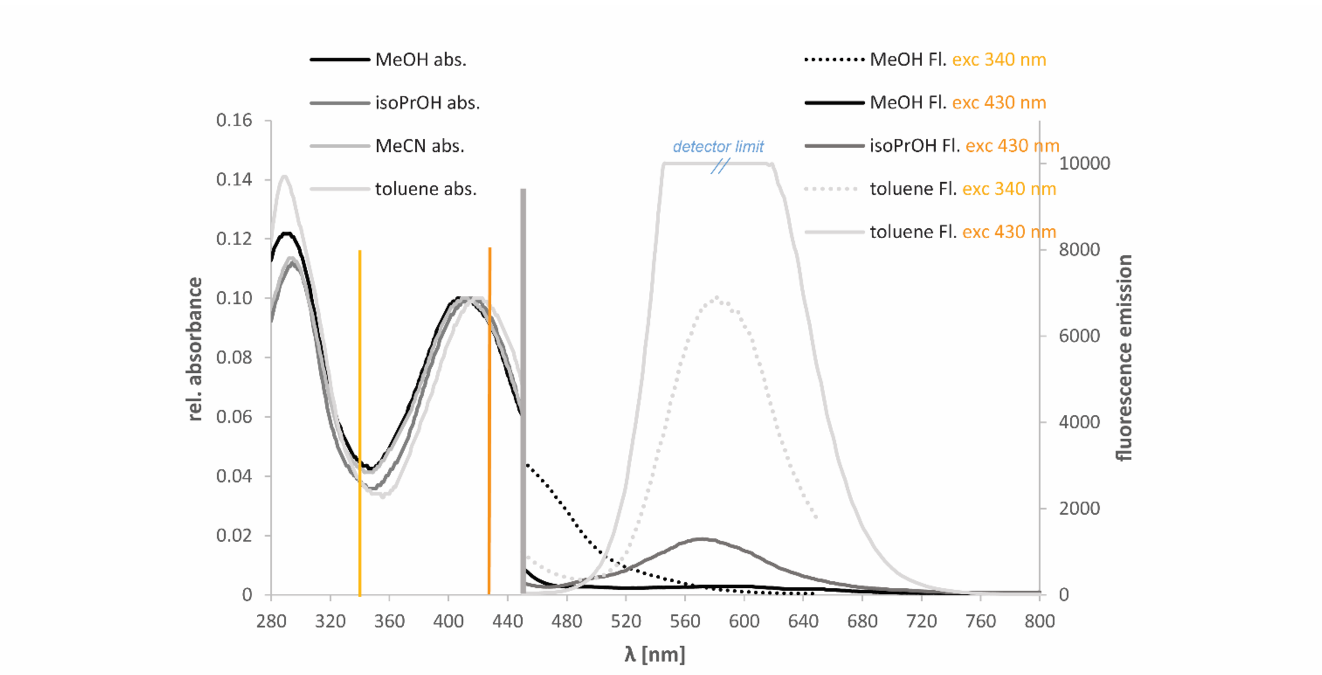

Figure S5 Absorbance and fluorescence emission spectra of **DTA-NDBF-OH** in various solvents (MeOH = methanol, isoPrOH = isopropanol, MeCN = acetonitrile and toluene). The values of λ(abs.max.) are between 410 (MeOH) and 422 nm (toluene).The fluorescence emission was detected between 370-650 nm (shown: 450-650) or 460-800 nm, depending on the excitation wavelength (340 or 430 nm, yellow strokes). The λ(em.max.) in toluene is 581 nm. With λexc. = 430 nm (and constant detection-conditions as for all derivatives) the detector limit was reached.

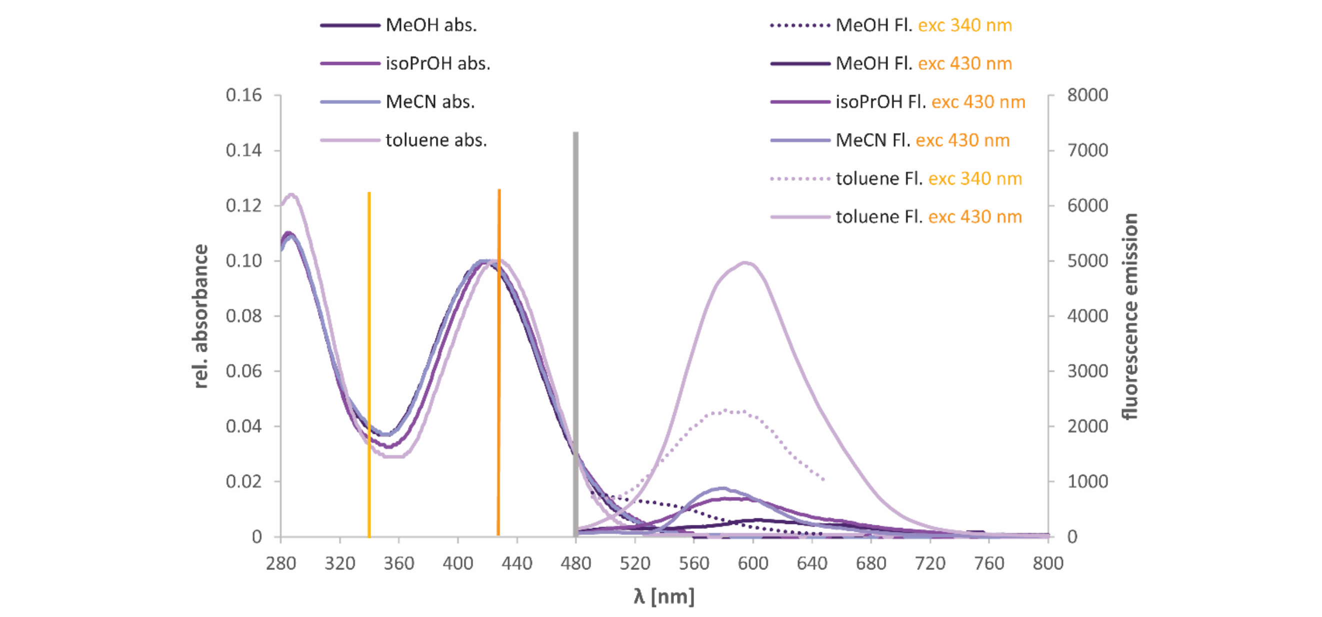

Figure S6 Absorbance and fluorescence emission spectra of **DAA-NDBF-OH** in various solvents (MeOH = methanol, isoPrOH = isopropanol, MeCN = acetonitrile and toluene). The values of λ(abs.max.) are between 418 (MeOH) and 427 nm (toluene).The fluorescence emission was detected between 370-650 nm (shown: 480-650) or 460-800 nm, depending on the excitation wavelength (340 or 430 nm, yellow strokes). The λ(em.max.) in toluene is 588 nm.

5. Two-photon-induced-fluorescence (TPiF) setups and data

“Setup A” for **DMA-**, **DTA-**, **DAA-** and **unsubstituted NDBF**: A Chameleon Ultra II, 80 MHz laser system (*APE Berlin* and *Coherent Inc*.) was used to generate pulsed laser excitation of wavelengths in the range from 700-1060 nm. Using a 1000 nm reflection/705 nm transition dichroic mirror (AHF T700spxr-1500) the expanded laser beam was reflected into the back-aperture of a water immersion IR microscope objective (UPlanApo/IR 60×1.20 W) in a confocal microscope setup (microscope body IX71, *Olympus*). To ensure a constant two-photon excitation power throughout the entire spectral range, a calibrated power meter head (coherent LM-2 VIS) was installed at a fixed point of 5 cm above the microscope objective. A linear variable neutral density filter (NDL-10C-2, *Thorlabs*) was used before the microscope body to keep the two-photon excitation power at 1 mW at the power meter head. To effectively suppress any IR excitation light in the detection path two IR-block filters (AHF HC770/SP) were used. The fluorescence after two-photon excitation was detected by an electron multiplying charge coupled device (EMCCD) camera (iXonEM + 897 back-illuminated, *Andor Technology*). The emission spots recorded with the camera were integrated for each excitation wavelength and corrected for background noise.

“Setup B” for **DMA-NDBF-OH** and **Az-NDBF-OH**: The TPiF experiments at “setup B” were carried out using a self-built system containing a wavelength-tunable titanium-doped sapphire (Ti:Sa) laser (Tsunami laser, *Spectra-Physics*, Darmstadt, Germany), which is pumped by a Millennia (*Spectra-Physics*) continuous wave (cw) laser with a neodymium-doped yttrium vanadate ($Nd:YVO_4$) medium. The wavelength variation of the Tsunami laser (rep. rate 80 MHz) can be obtained by choosing different slit-width between the inner prisms (theoretical accessible wavelength range: 730-930 nm, used: 760-840 nm). The output enters directly a prism compressor to compensate the later introduced chirp. After that, the laser beam can be adjusted in its intensity due to a polarizer. A fiber spectrometer is added to monitor the spectral pulse shape. The laser pulse is then focused in the 1x10 mm cuvette, which contains the sample by using a microscope objective (Plan N, 20/0.40, *Olympus*, Tokyo, Japan). If the sample is able to emit fluorescence upon two-photon excitation, the signal is collected with a second objective (UPlanFL N, 20x/0.50, *Olympus*). Both objectives can be moved in each dimension due to home-built translation stages. The excitation pulse is excluded from the fluorescence signal by the use of two filters, namely a short-pass dichroic mirror (FF670-SDi01, *Semrock*, Rochester, USA) and a band-pass filter (BG38, *Schott*, Jena, Germany). Due to that the fluorescence signal can be collected via two mirrors to the spectrograph (SpectraPro 300i, *Acton Research*, Munich, Germany) which is equipped with a CCD-camera (charge-coupled device, 400 Pixel x 1340 Pixel, *Roper Scientific*, Munich, Germany). The WinSpec program (*Roper Scientific*) on the computer shows the spectra obtained during the measurements. The concentrations of the photocage samples were adjusted to approximately 100 µM, the integration time was set to 200 ms. The pulse power was set to 400 mW in toluene and 600 mW in DMSO.

The quadratic power dependency was tested with both setups, which is shown in Figures S7 & S8.

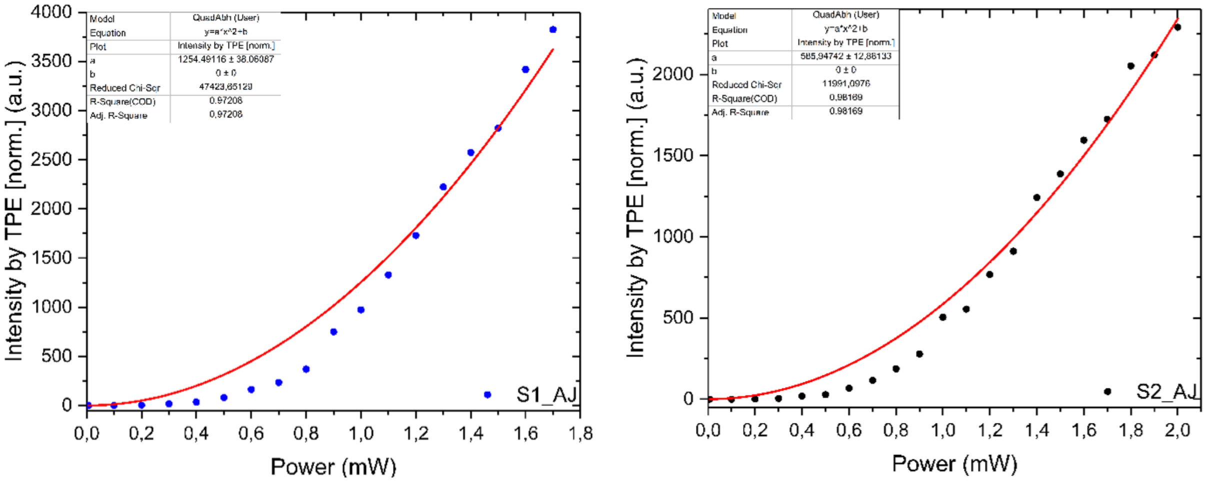

Figure S7 Quadratic dependency of the two-photon-induced-fluorescence (TPiF). The emission intensity of **DTA-** (left) and **DAA-NDBF-OH** after two-photon excitation is plotted against the used excitation beam power (mW). The used fit ($y = ax^2+b$) is shown in red.

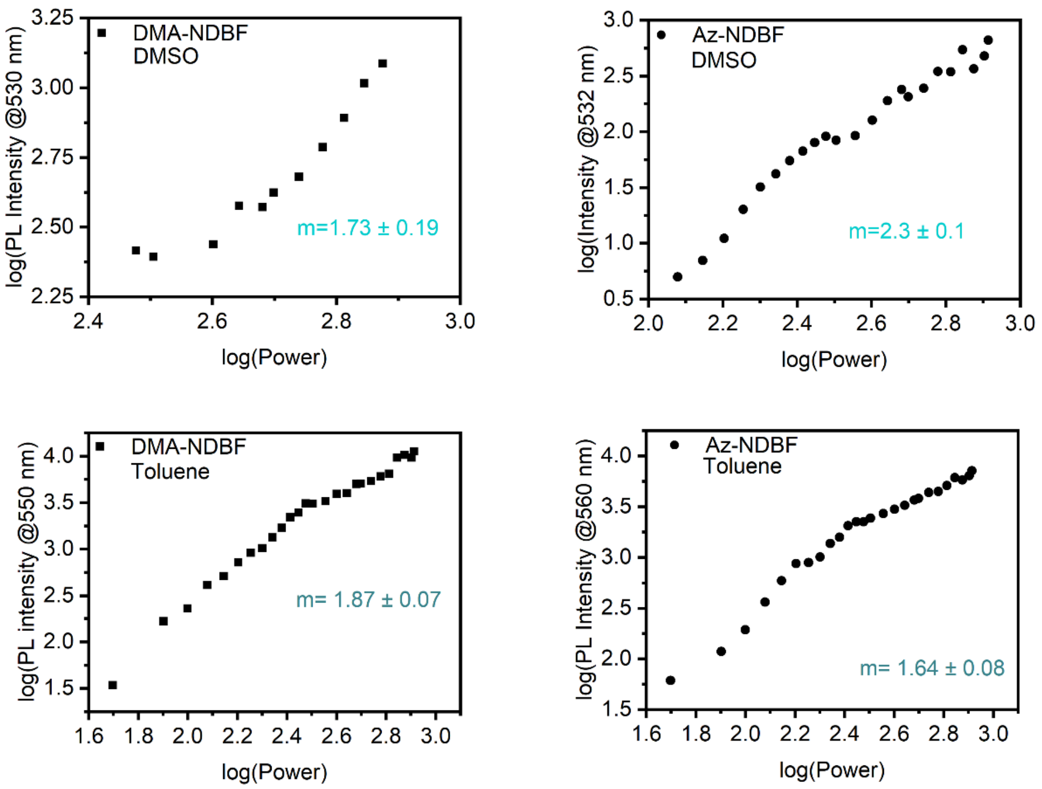

Figure S8 The emission intensity of **DMA-** (left) and **Az-NDBF-OH** in DMSO and toluene after two-photon excitation (λexc. = 550 & 560 nm) is plotted logarithmically against the used excitation beam power (mW).The slope *m* (~2) is given in each diagram.

6. Computational methods and data

For the excited state analysis in different solvents we used a polarizable continuum model (PCM) for toluene (ϵ = 2.379, n^2 = 2.232) and methanol (ϵ = 32.63, n^2 = 1.758). Since we are using Gaussian 16 for a downstream task due to its advanced PCM capabilities, we carried out the optimizations and Tamm-Dancoff approximation (TDA) computations also in this program. The computational results are shown in Tables S5 and S6 for **DMA-NDBF-OH**, and Tables S7 and S8 for **Az-NDBF-OH**.

Table S5: Excited state summary of **DMA-NDBF-OH** with Q-Chem.

	vacuum				toluene				methanol			
State	E_{exc} [eV]	f	$\Delta\mu$ [D]	d_{eh} [Å]	E_{exc} [eV]	f	$\Delta\mu$ [D]	d_{eh} [Å]	E_{exc} [eV]	f	$\Delta\mu$ [D]	d_{eh} [Å]
S_1	3.84	0.63	7.88	2.62	3.60	0.91	12.59	3.42	3.50	0.89	16.11	3.84
S_2	4.19	0.20	1.71	0.72	4.16	0.08	-0.04	0.32	4.16	0.05	0.12	0.34
S_3	4.37	0.03	4.35	1.47	4.29	0.05	5.65	1.64	4.24	0.04	6.43	1.70

Table S6: Excited state summary of **DMA-NDBF-OH** with Gaussian.

	vacuum		toluene		methanol	
State	E_{exc} [eV]	f	E_{exc} [eV]	f	E_{exc} [eV]	f
S_1	3.84	0.64	3.63	0.89	3.54	0.88
S_2	4.19	0.20	4.15	0.08	4.15	0.05
S_3	4.37	0.03	4.30	0.04	4.25	0.04

Table S7: Excited state summary of **Az-NDBF-OH** with Q-Chem.

State	vacuum				toluene				methanol			
	E_{exc} [eV]	f	$\Delta\mu$ [D]	d_{eh} [Å]	E_{exc} [eV]	f	$\Delta\mu$ [D]	d_{eh} [Å]	E_{exc} [eV]	f	$\Delta\mu$ [D]	d_{eh} [Å]
S_1	3.82	0.68	8.26	2.71	3.59	0.95	12.74	3.43	3.49	0.93	16.20	3.83
S_2	4.18	0.19	1.36	0.61	4.16	0.08	-0.17	0.31	4.16	0.05	-0.15	0.32
S_3	4.38	0.03	4.25	1.44	4.30	0.04	5.52	1.61	4.25	0.04	6.44	1.70

Table S8: Excited state summary of **Az-NDBF-OH** with Gaussian.

State	vacuum		toluene		methanol	
	E_{exc} [eV]	f	E_{exc} [eV]	f	E_{exc} [eV]	f
S_1	3.83	0.68	3.60	0.94	3.50	0.93
S_2	4.18	0.19	4.15	0.08	4.15	0.05
S_3	4.38	0.03	4.30	0.04	4.25	0.04

The deviations for vacuum geometries between the two employed programs is negligible for both compounds and is only due to slightly different implementations of the exchange correlation functional. Since the PCM implementations in Q-Chem and Gaussian are not identical, these results differ slightly more, but the differences are still minor. Attachment and detachment densities for the S_1 and S_2 states of **DMA-NDBF-OH** were visualized using VMD[4], shown in the main text, Figure 5.

The lowest excited states of both compounds correspond to a charge-transfer (CT) excitation, primarily indicated by the increase in dipole moment $\Delta\mu$. The electron-hole distances d_{eh} (the larger the value, the larger is the CT character) corroborate this finding. The transition to the S_2 corresponds to a local excitation, also visible from the excited state dipole moments and electron-hole distances. The CT state is slightly red-shifted due to the stabilizing effect of the polarizable solvent, whereas the excitation energy of the LE state remains almost unchanged. In contrast to the experimental data, a red-shift is observed in the computational results when comparing toluene and methanol as solvents. This is most likely to the lack of explicit hydrogen bonds in the employed solvent model to the amino group, which would actually counteract the stabilizing effect of the polar solvent. To obtain the fluorescence energy of both compounds in vacuum, the ground state geometry was first optimized using Gaussian 16[5] with CAM-B3LYP/def2-svp. Afterwards, excitation energies were computed at the same level of theory employing TDA. The geometry of the energetically lowest singlet excited state was then optimized, and a true minimum was confirmed through frequency analysis. For the fluorescence energy in solution with a PCM, the following protocol was employed (adapted from https://gaussian.com/scrf/):

1. Ground state optimization (equilibrium solvation)
2. Vertical excitation energies (non-equilibrium solvation)
3. Excited state optimization of S_1 (equilibrium solvation)
4. State-specific emission, *i.e.*, vertical excitation energies of the S_1 geometry (equilibrium solvation)
5. Ground state energy with equilibrium solvation of S_1 excited state

Step (2) yields vertical excitation energies of the PCM-solvated compound using a linear response formalism. The fluorescence energy is computed as the difference between the total energy of S_1 in step (4) and the ground state energy in step (5). Predefined PCM parameters for toluene and methanol were used as implemented in Gaussian 16. From our calculations, we conclude that the photochemistry, including shapes and ordering of the PES, of NDBF is highly solvent-sensitive. In our calculations, already the apolar solvent environment of toluene seems to give rise to a change in state ordering at the S_1 minimum geometry. Experimentally, we observed a clear trend from toluene to isopropanol to methanol to water.

7. References

[1] J. B. Grimm, B. P. English, J. Chen, J. P. Slaughter, Z. Zhang, A. Revyakin, R. Patel, J. J. Macklin, D. Normanno, R. H. Singer, T. Lionnet, L. D. Lavis, *Nat. Methods* **2015**, *12*, 244–250.

[2] Y. Becker, E. Unger, M. A. H. Fichte, D. A. Gacek, A. Dreuw, J. Wachtveitl, P. J. Walla, A. Heckel, *Chem. Sci*. **2018**, *9*, 2797-2802.

[3] H. Lusic, R. Uprety, A. Deiters, *Org. Lett.* **2010**, *12*, 916-919.

[4] W. Humphrey, A. Dalke, K. Schulten, *J. Mol. Graph*. **1996**, *14*, 33-38.

[5] M. J. Frisch, G. W. Trucks, H. B. Schlegel, G. E. Scuseria, M. A. Robb, J. R. Cheeseman, G. Scalmani, V. Barone, G. A. Petersson, H. Nakatsuji, X. Li, M. Caricato, A. V. Marenich, J. Bloino, B. G. Janesko, R. Gomperts, B. Mennucci, H. P. Hratchian, J. V. Ortiz, A. F. Izmaylov, J. L. Sonnenberg, D. Williams-Young, F. Ding, F. Lipparini, F. Egidi, J. Goings, B. Peng, A. Petrone, T. Henderson, D. Ranasinghe, V. G. Zakrzewski, J. Gao, N. Rega, G. Zheng, W. Liang, M. Hada, M. Ehara, K. Toyota, R. Fukuda, J. Hasegawa, M. Ishida, T. Nakajima, Y. Honda, O. Kitao, H. Nakai, T. Vreven, K. Throssell, J. A. Montgomery, Jr., J. E. Peralta, F. Ogliaro, M. J. Bearpark, J. J. Heyd, E. N. Brothers, K. N. Kudin, V. N. Staroverov, T. A. Keith, R. Kobayashi, J. Normand, K. Raghavachari, A. P. Rendell, J. C. Burant, S. S. Iyengar, J. Tomasi, M. Cossi, J. M. Millam, M. Klene, C. Adamo, R. Cammi, J. W. Ochterski, R. L. Martin, K. Morokuma, O. Farkas, J. B. Foresman and D. J. Fox*, Gaussian˜16 Revision C.01* **2016**, Gaussian Inc. Wallingford CT.

8. NMR spectra of small molecules

5-(azetidin-1-yl)-2-iodophenol (3a) <u>Derivative a , R = azetidinyl</u>

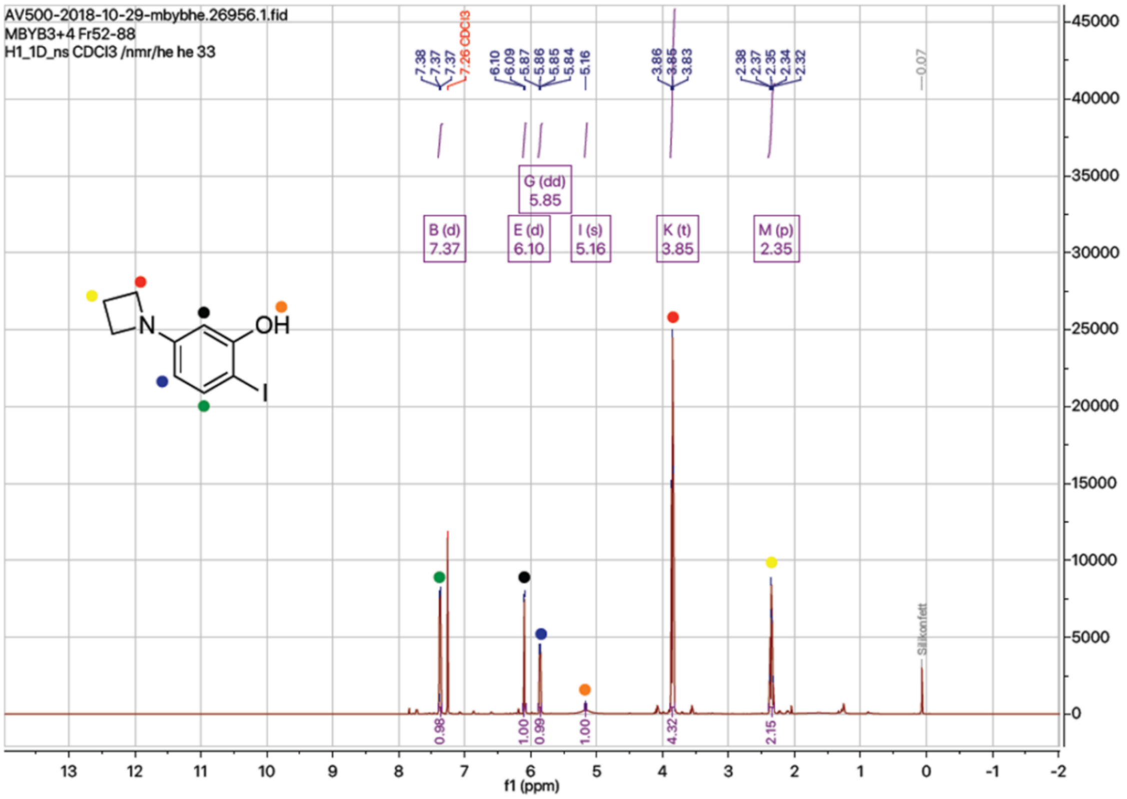

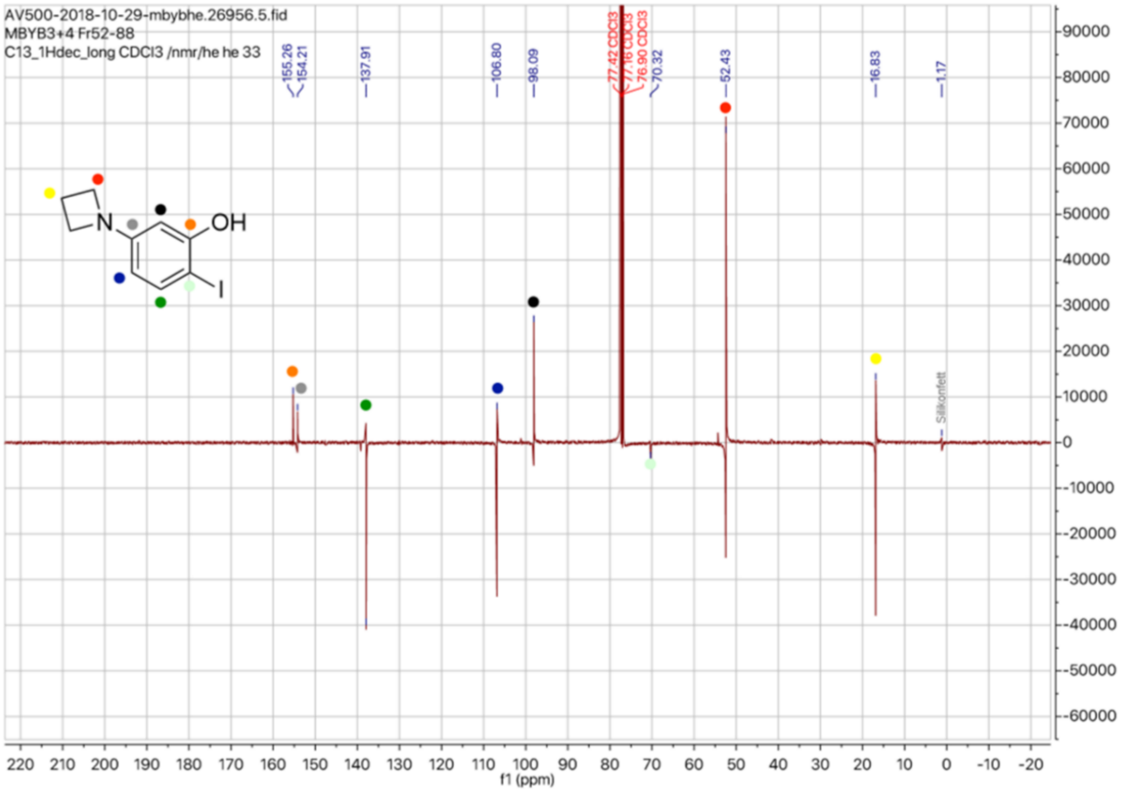

4-(5-(azetidin-1-yl)-2-iodophenoxy)-2-nitrobenzaldehyde (5a) Derivative a , R = azetidinyl

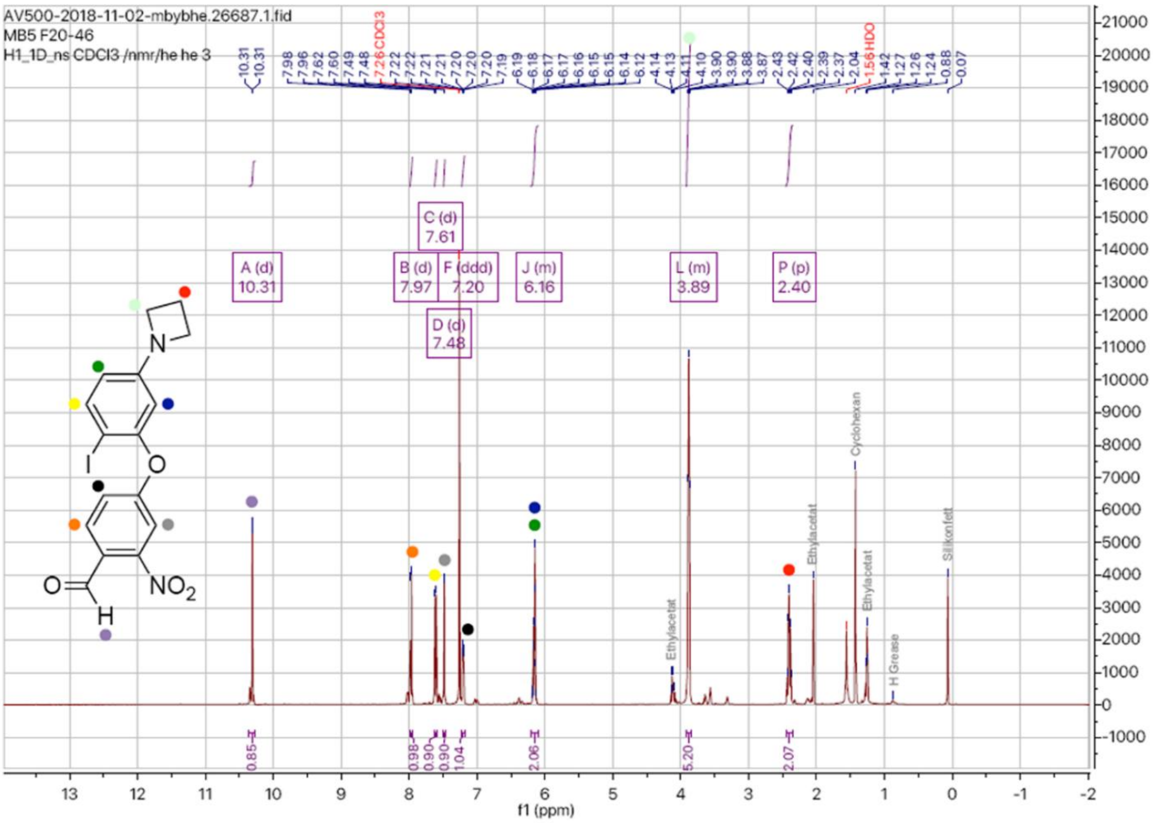

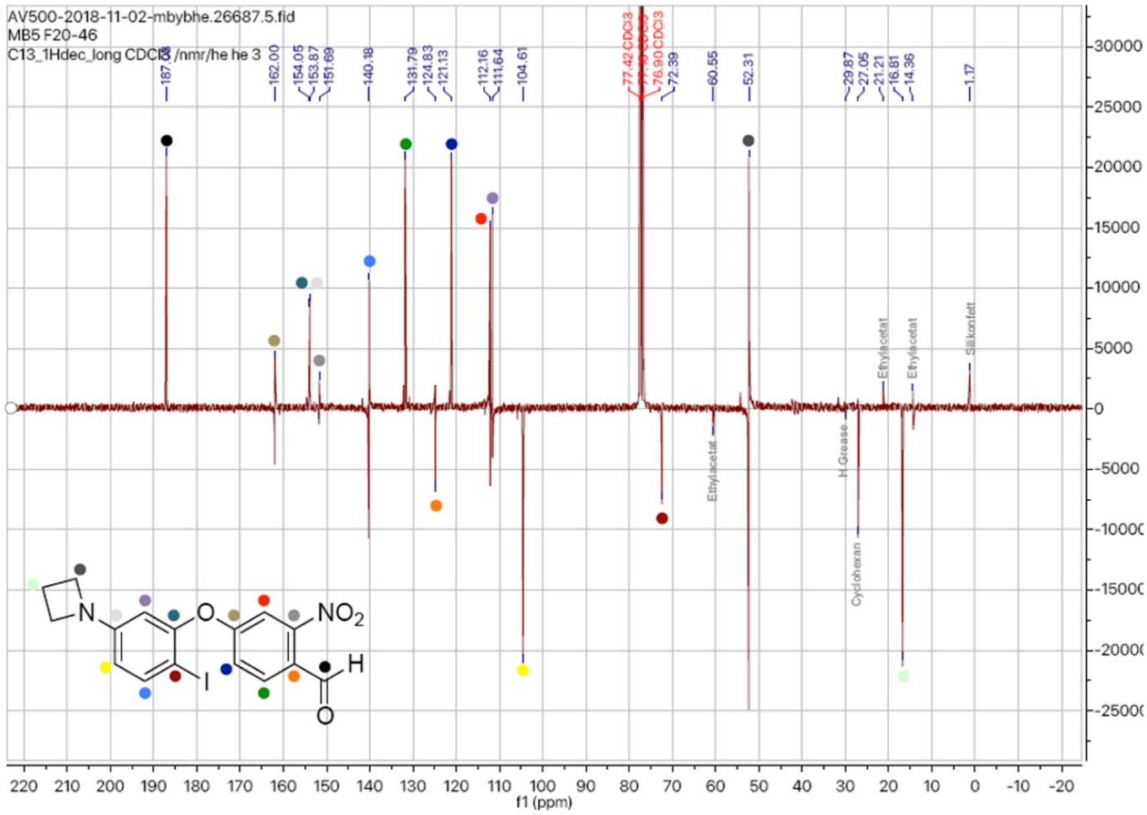

1-(4-(5-(azetidin-1-yl)-2-iodophenoxy)-2-nitrophenyl)ethan-1-ol (6a)

<u>Derivative a , R = azetidinyl</u>

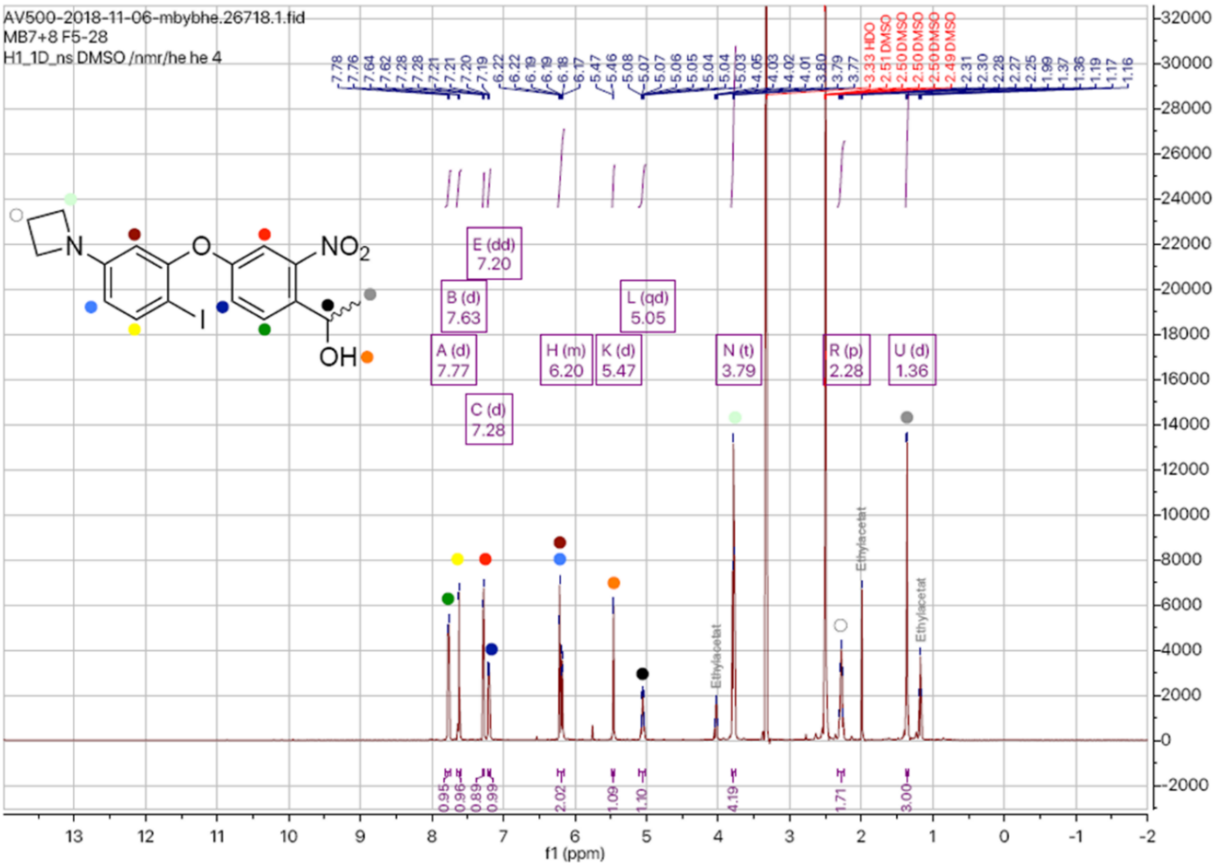

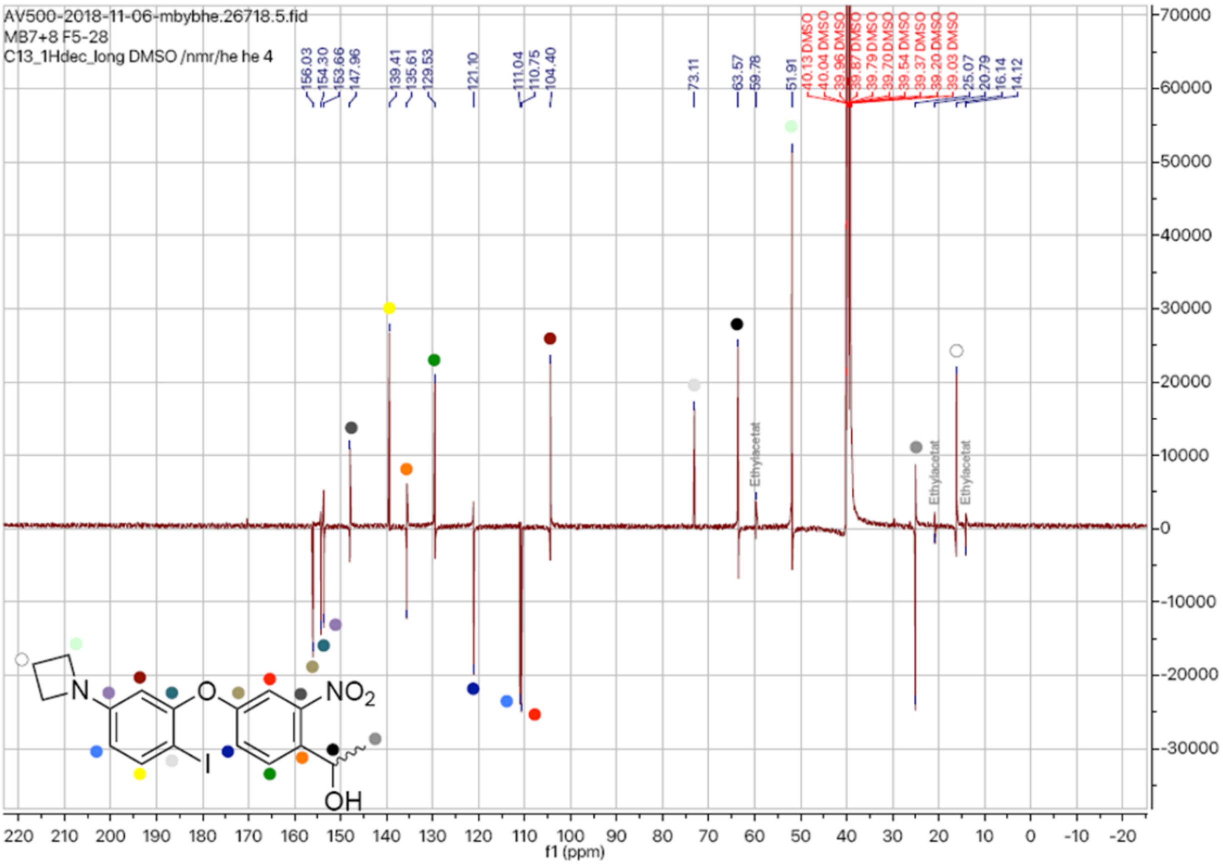

1-(7-(azetidin-1-yl)-3-nitrodibenzo[*b*,*d*]furan-2-yl)ethan-1-ol (Az-NDBF-OH) **Derivative a , R = azetidinyl**

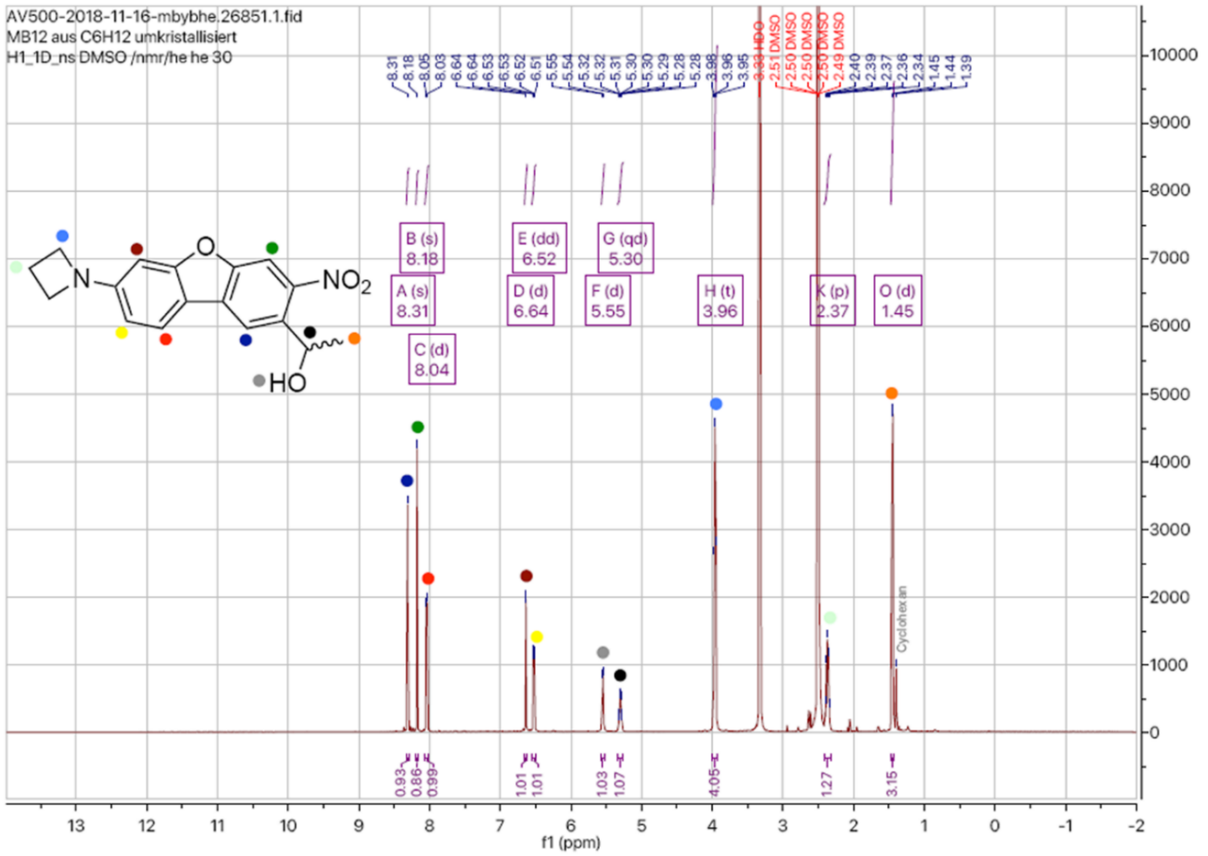

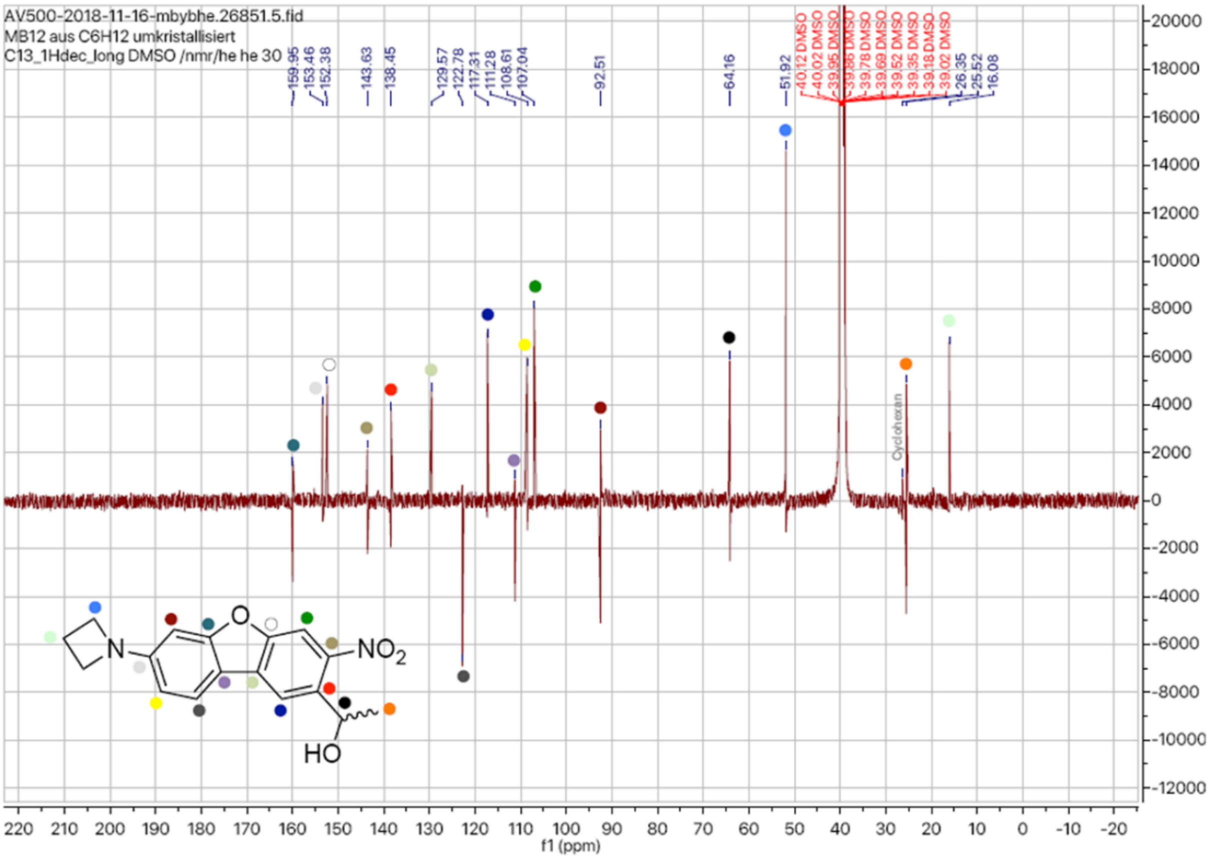

1-(7-(azetidin-1-yl)-3-nitrodibenzo[*b*,*d*]furan-2-yl)ethyl (4-nitrophenyl) carbonate (7a) Derivative a , R = azetidinyl

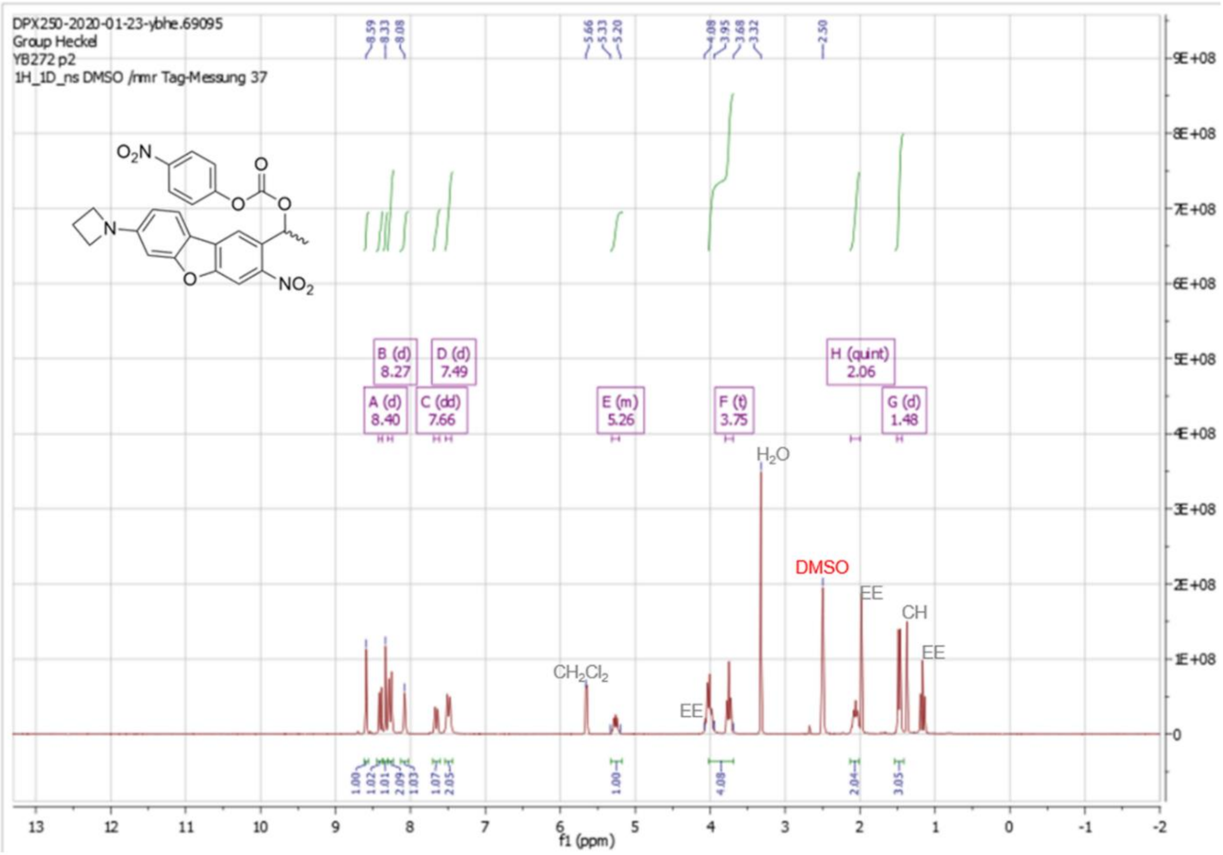

((1-(7-(azetidin-1-yl)-3-nitrodibenzo[*b*,*d*]furan-2-yl)ethoxy)carbonyl)-*L*-glutamic acid (Az-NDBF-Glu)

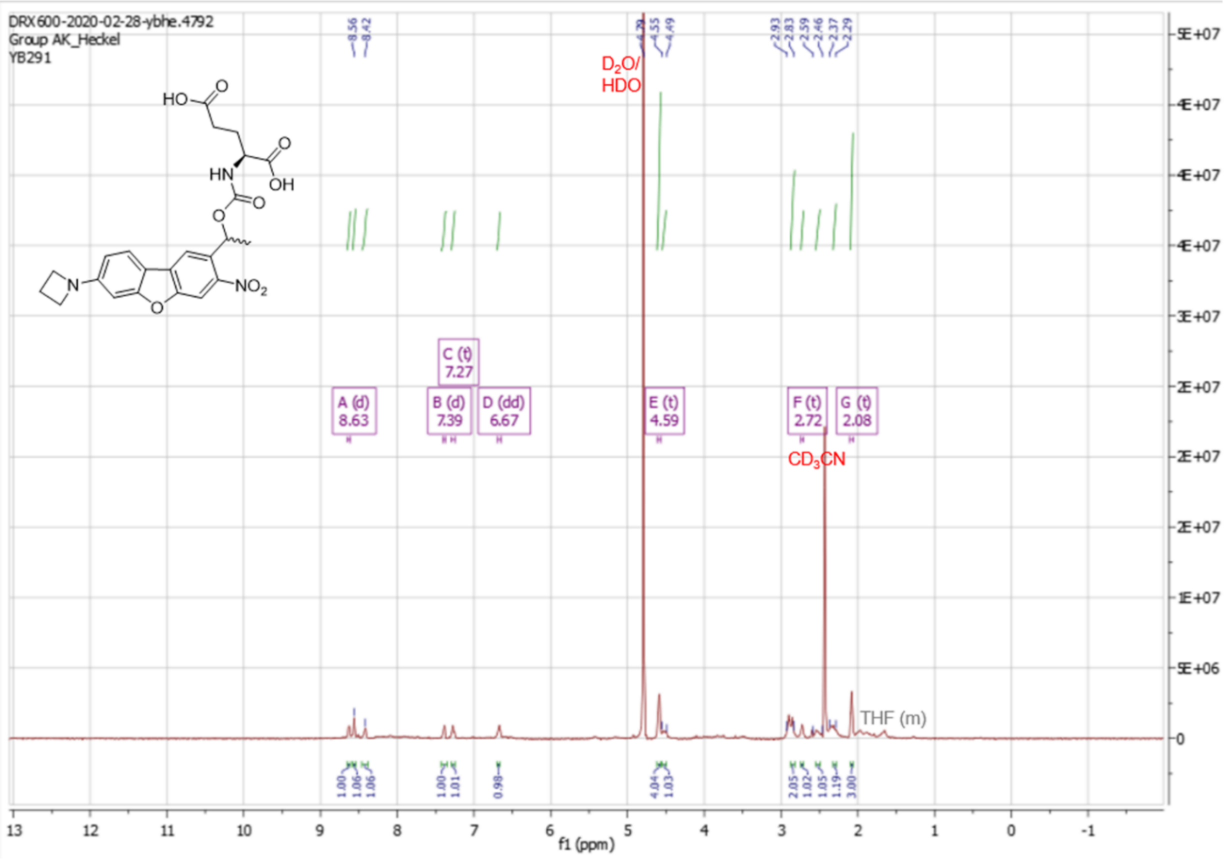

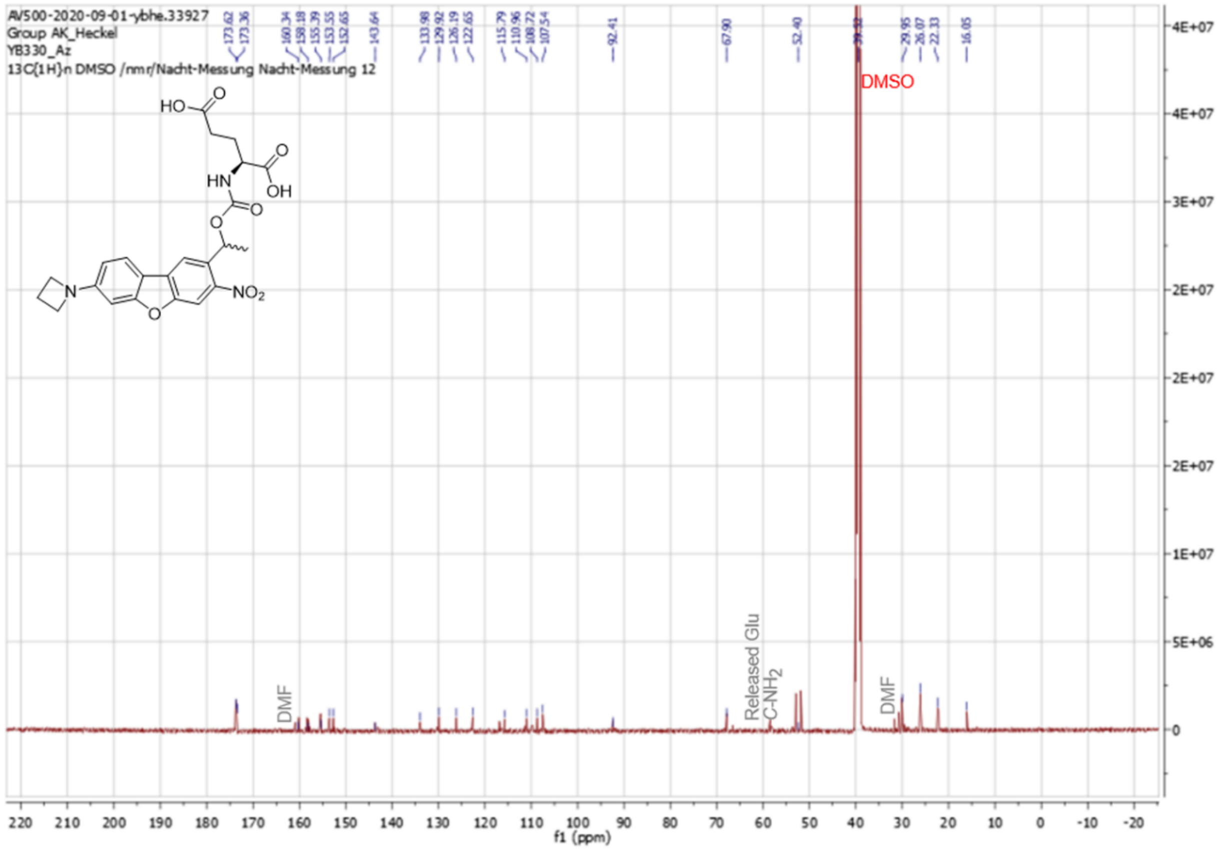

3-(di-*p*-tolylamino)phenol (2b)

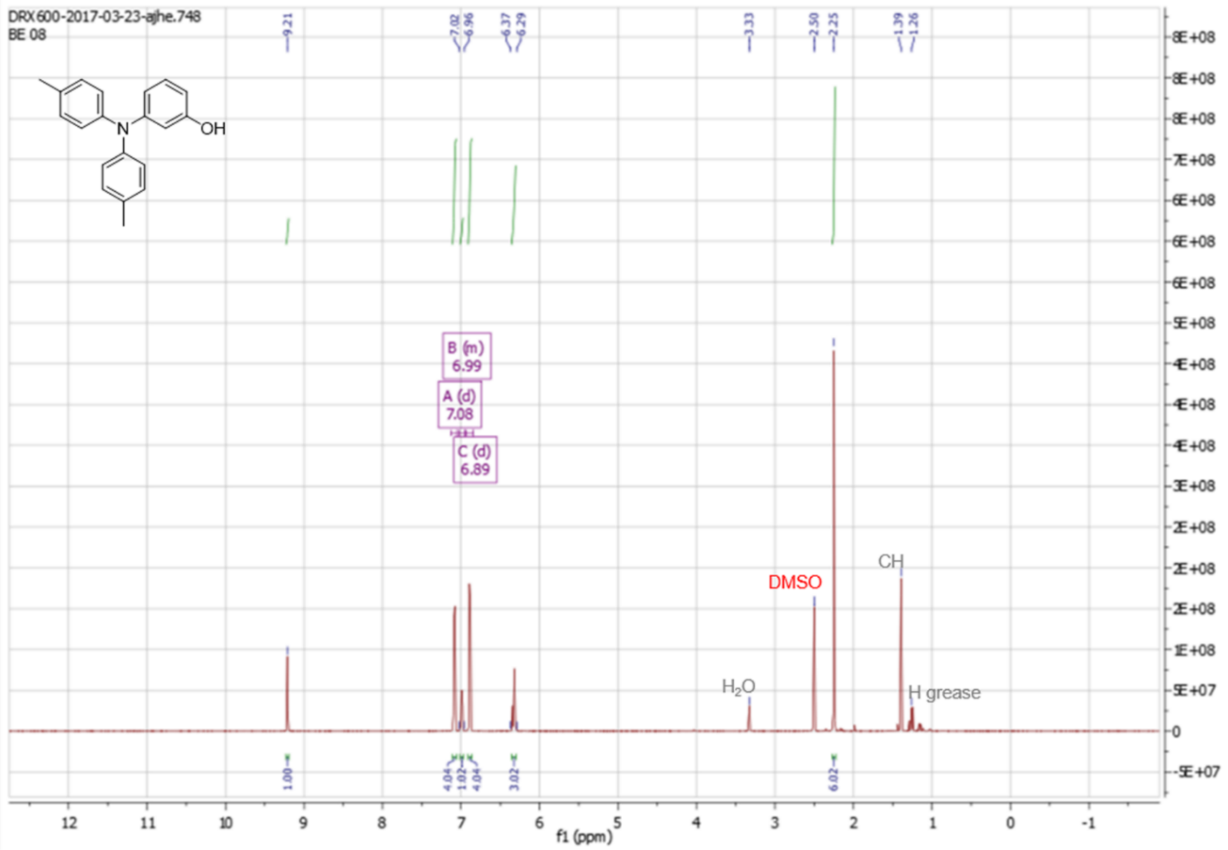

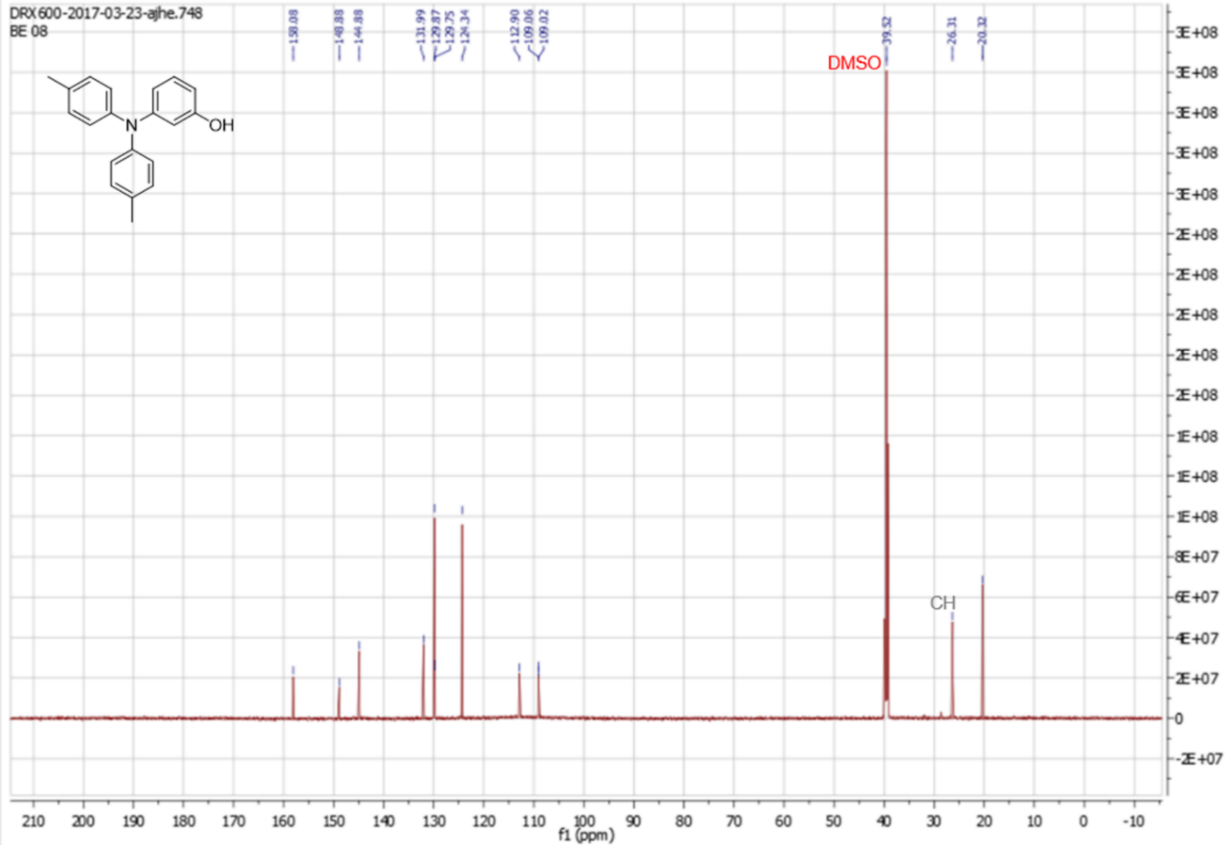

3-(bis(4-methoxyphenyl)amino)phenol (2c) <ins>Derivatives b & c , R = ditolyl- & dianisyl-amino- = DTA & DAA-phenyl</ins>

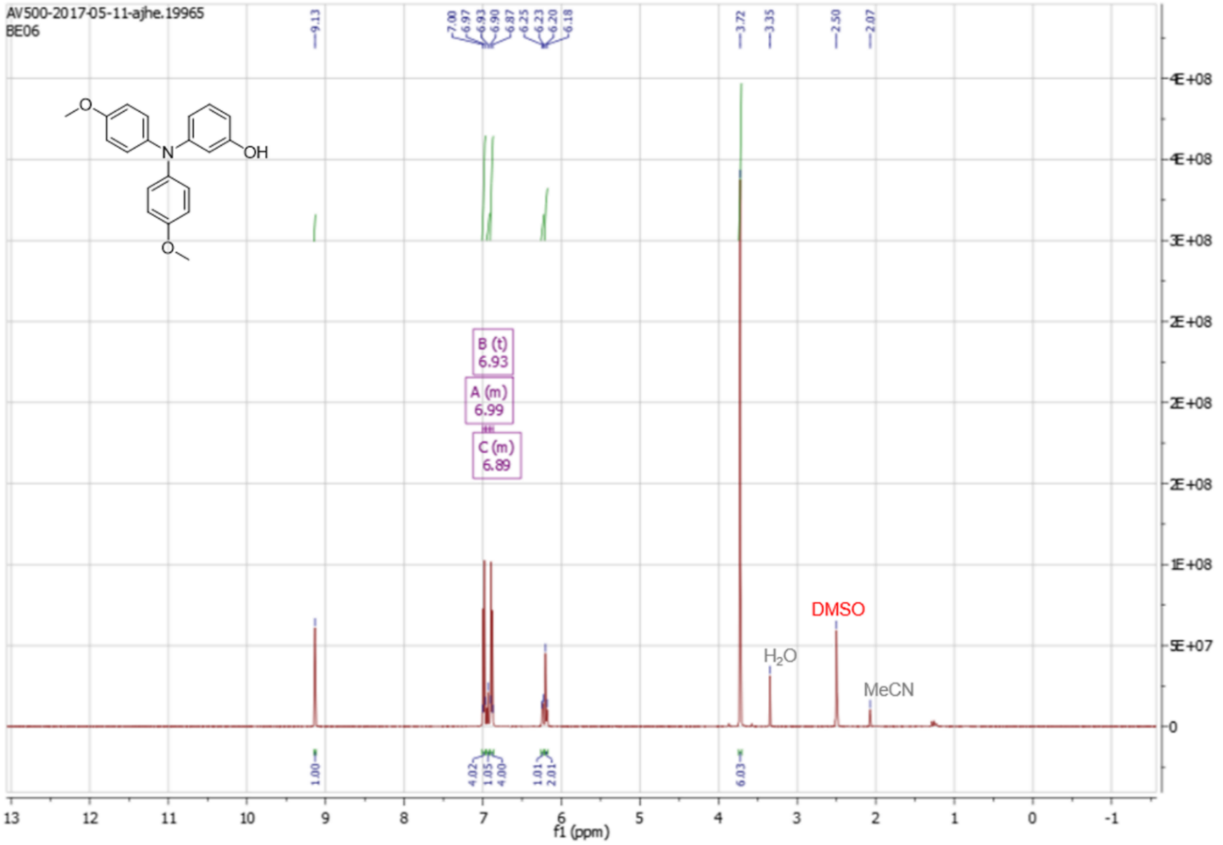

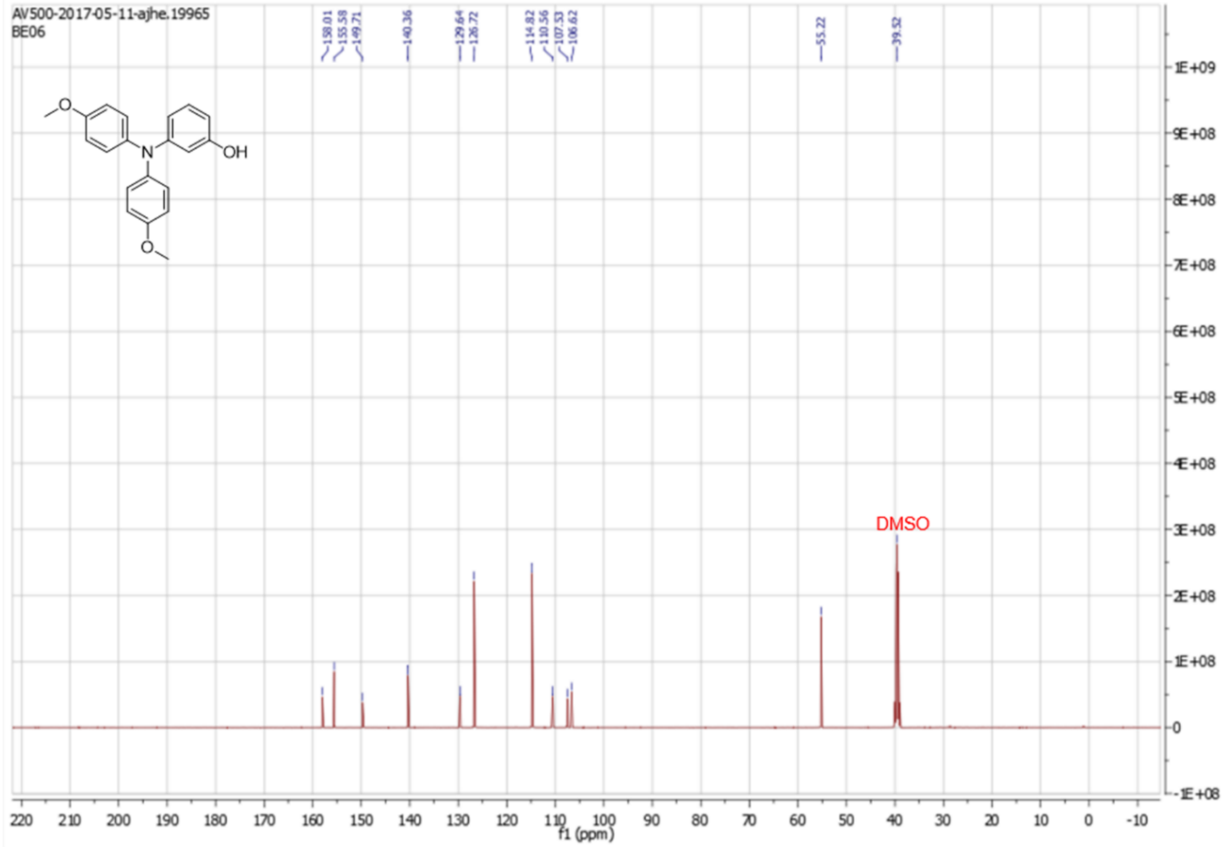

5-(di-*p*-tolylamino)-2-iodophenol (3b)

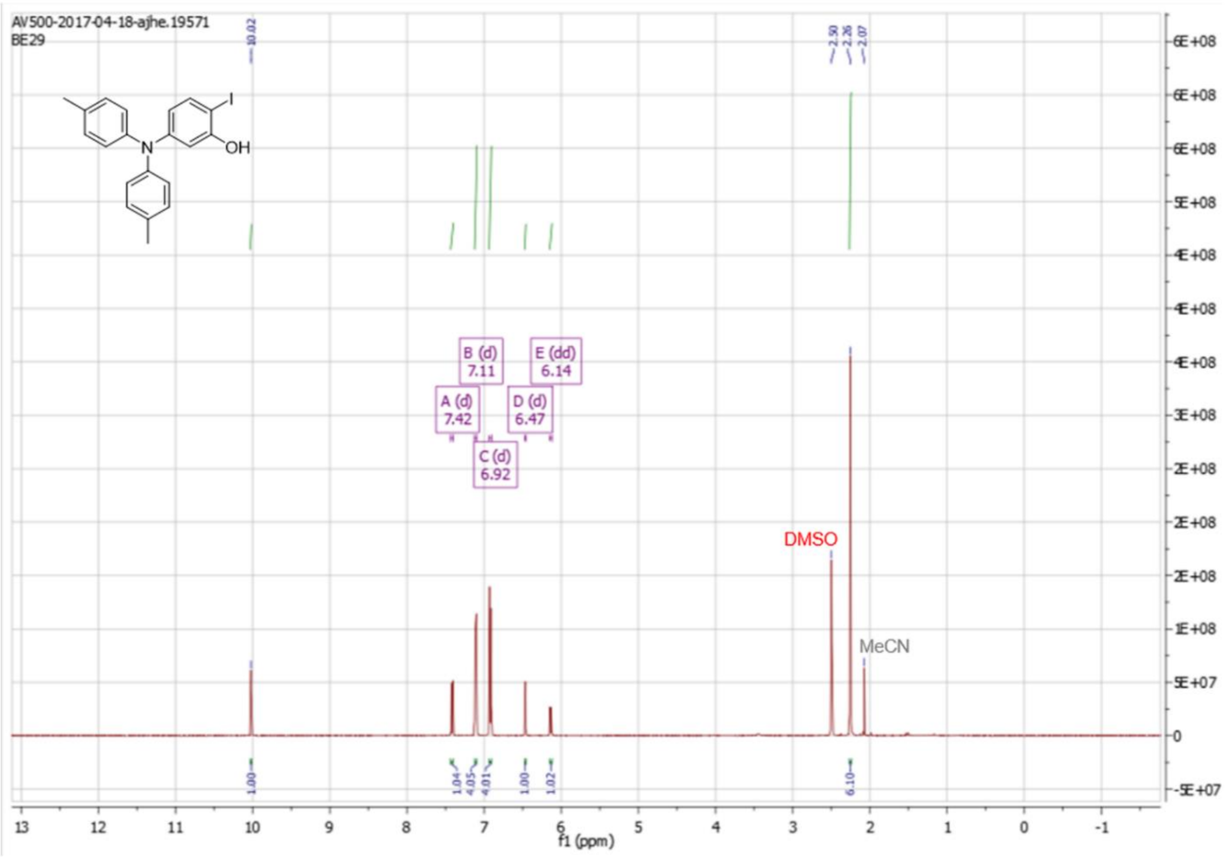

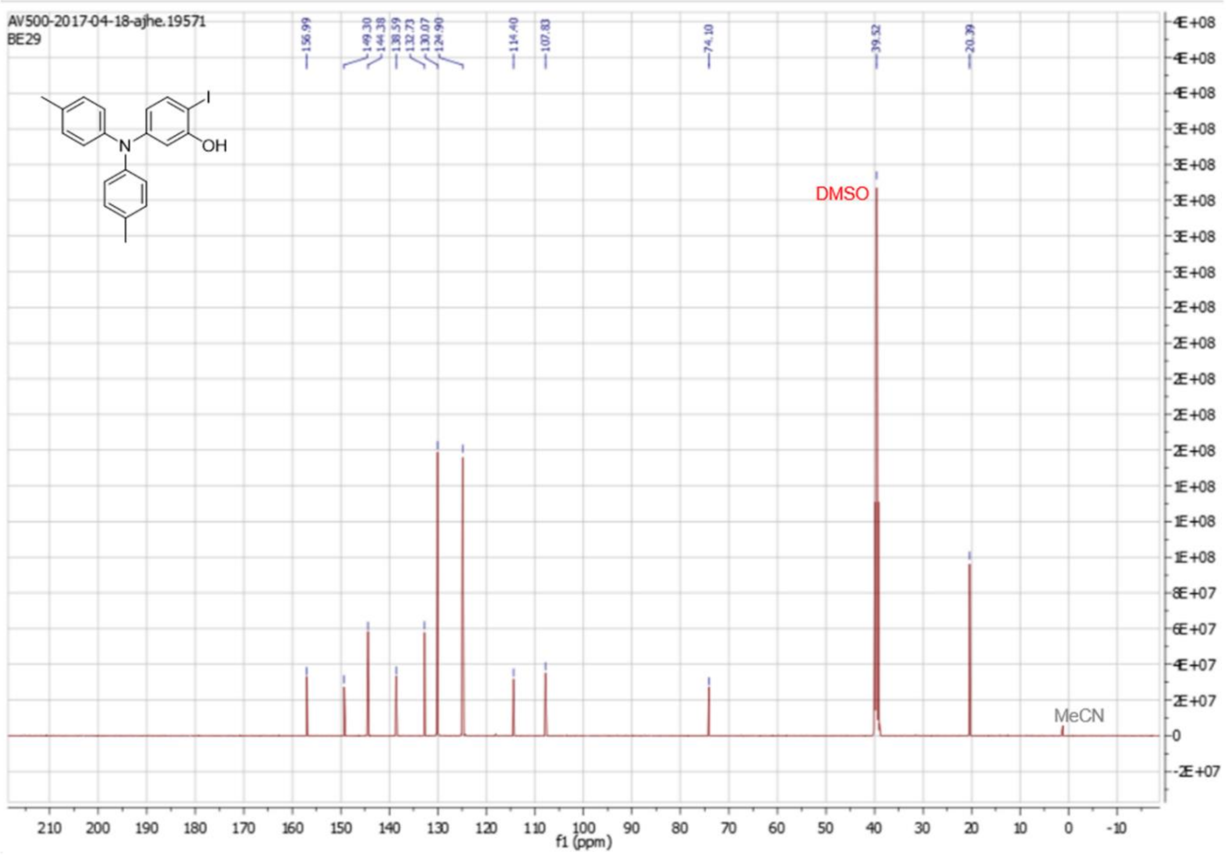

5-(bis(4-methoxyphenyl)amino)-2-iodophenol (3c) Derivatives b & c , R = ditolyl- & dianisyl-amino- = DTA & DAA-phenyl

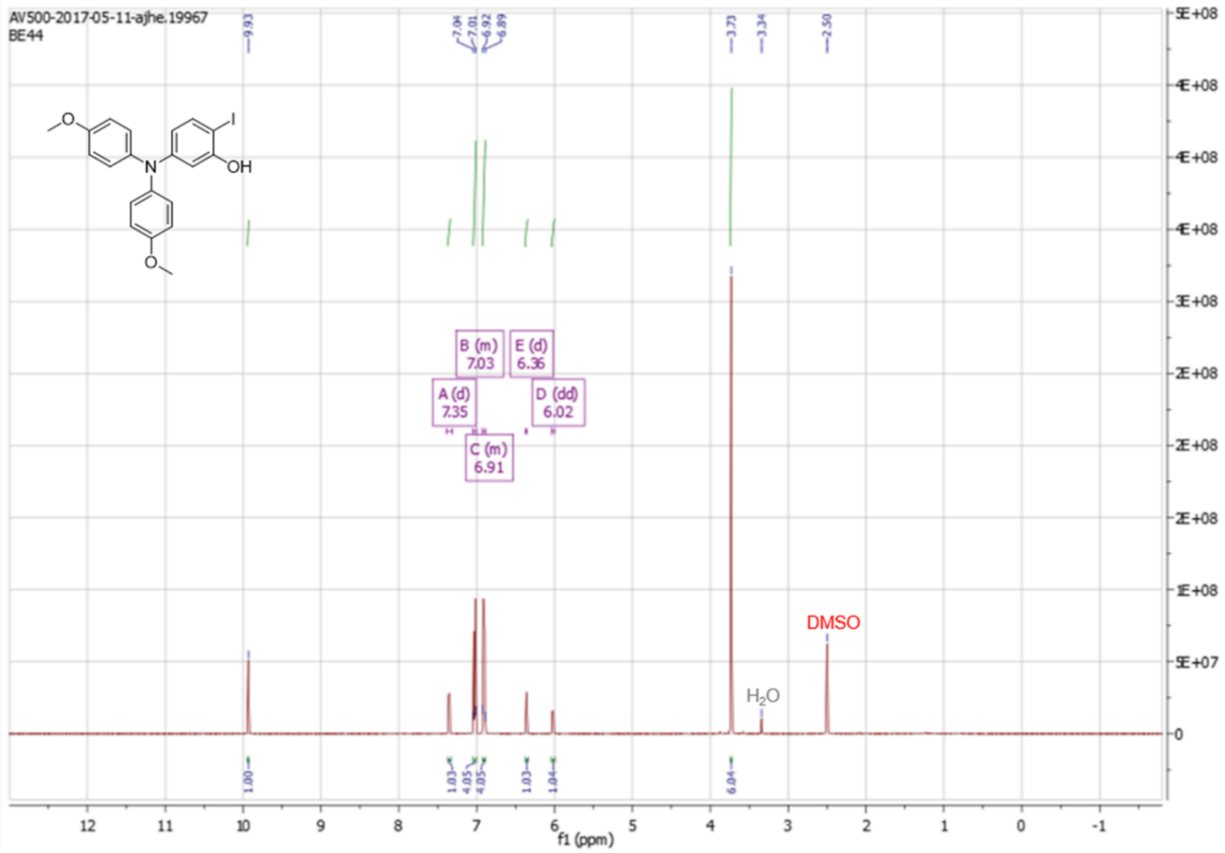

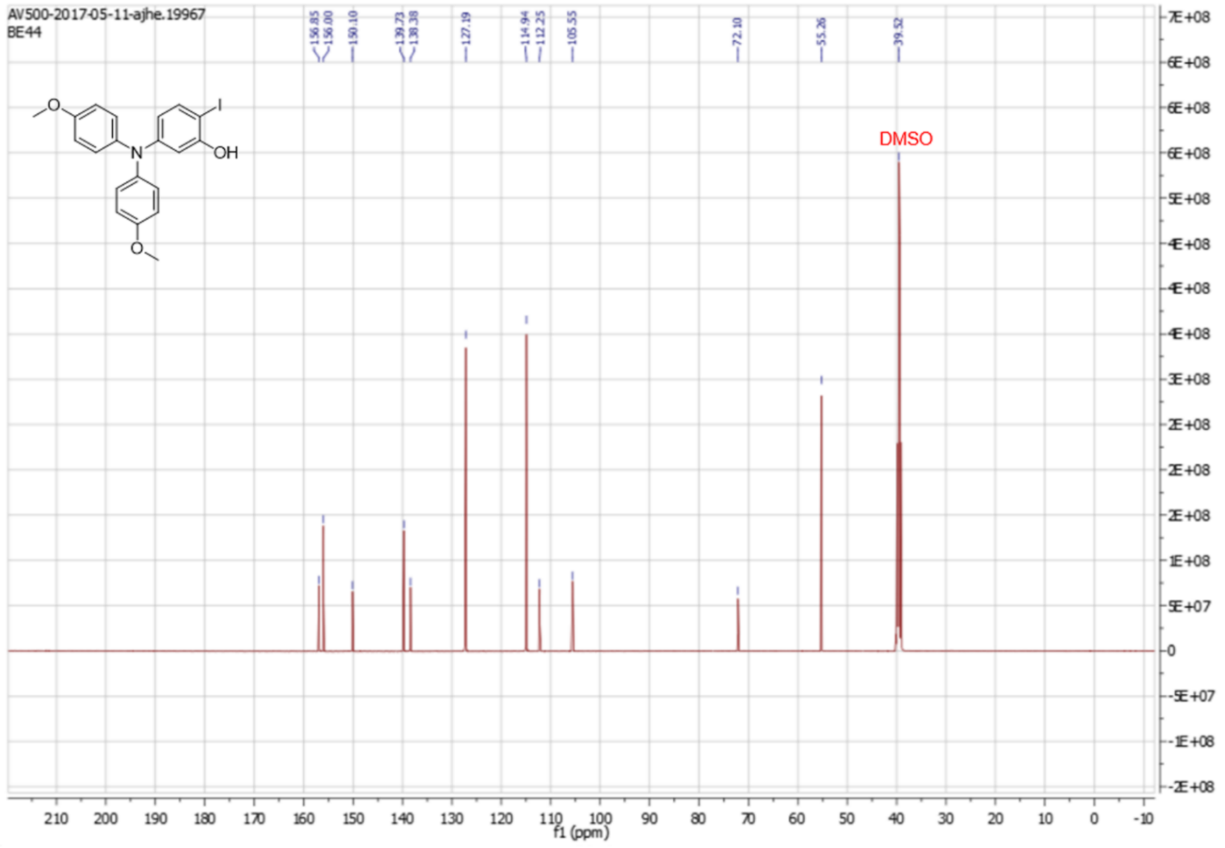

4-(5-(di-*p*-tolylamino)-2-iodophenoxy)-2-nitrobenzaldehyde (5b)

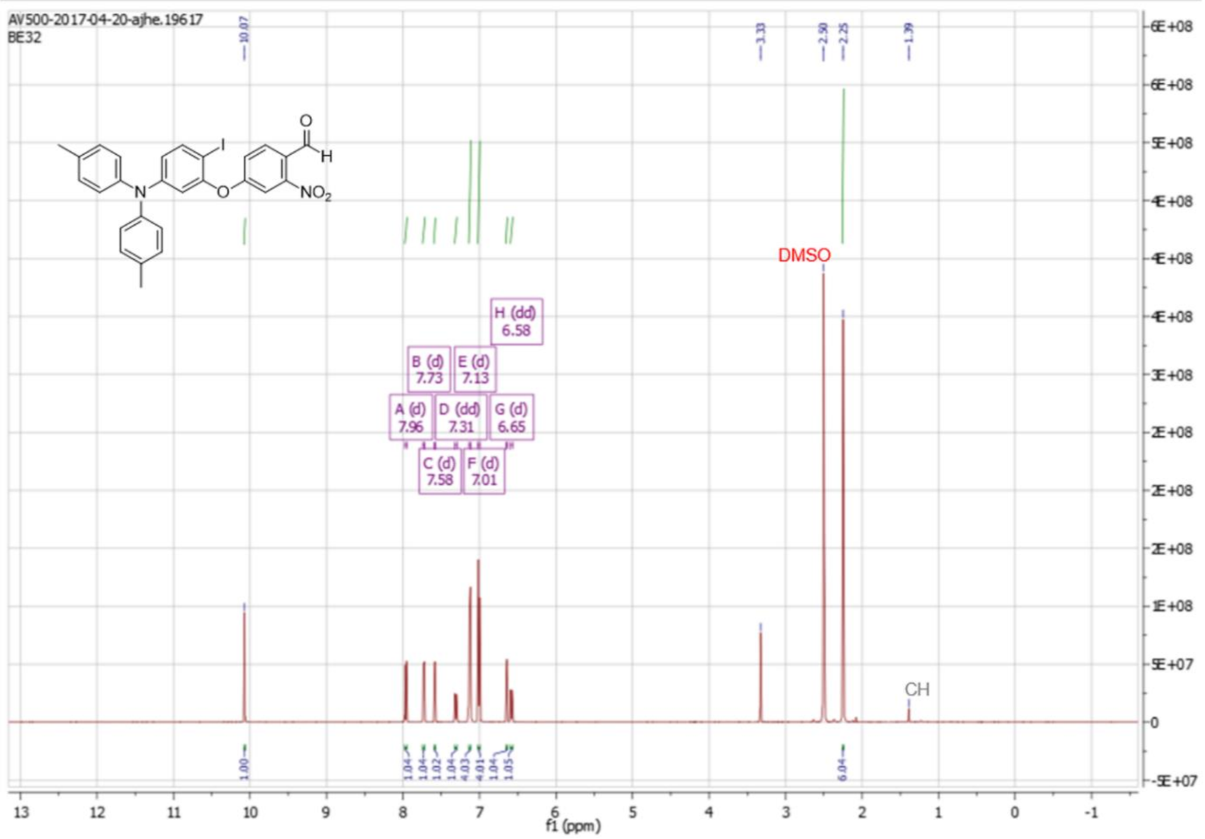

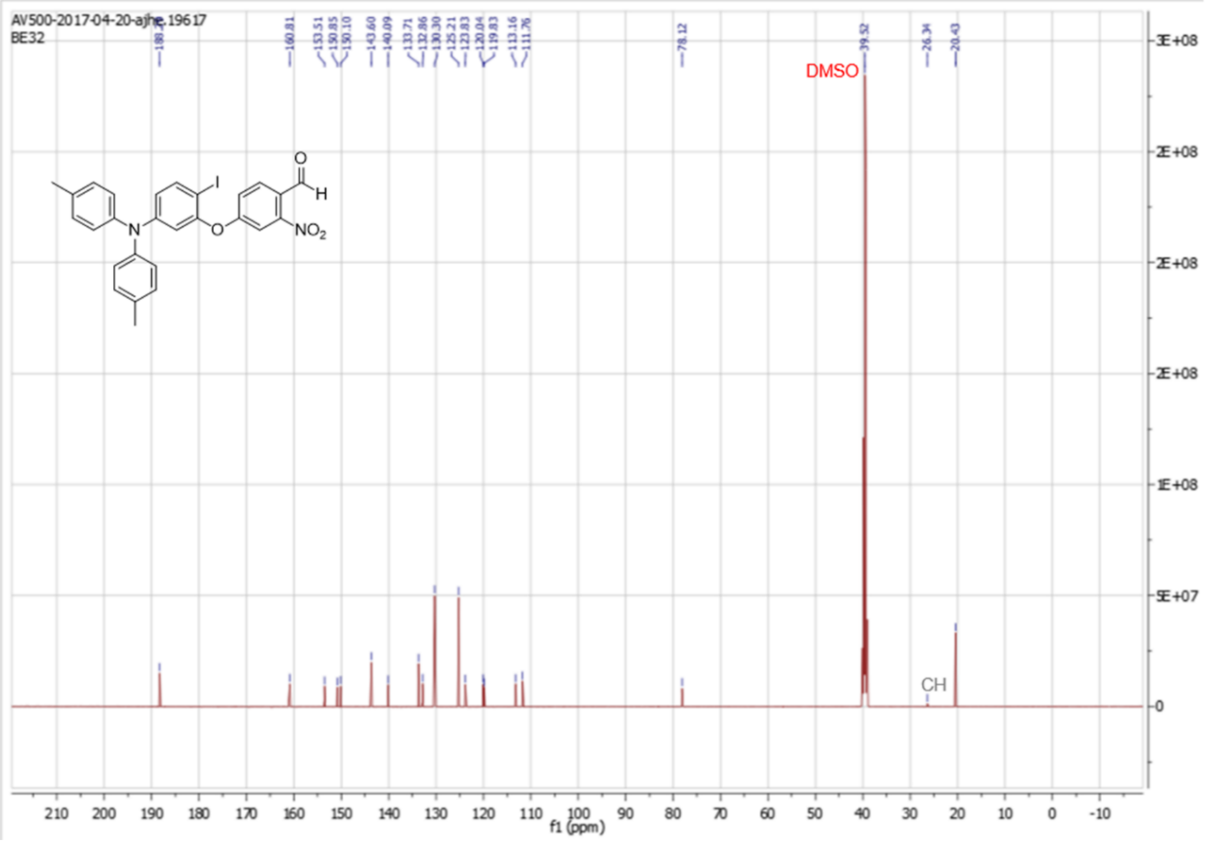

4-(5-(bis(4-methoxyphenyl)amino)-2-iodophenoxy)-2-nitrobenzaldehyde (5c)

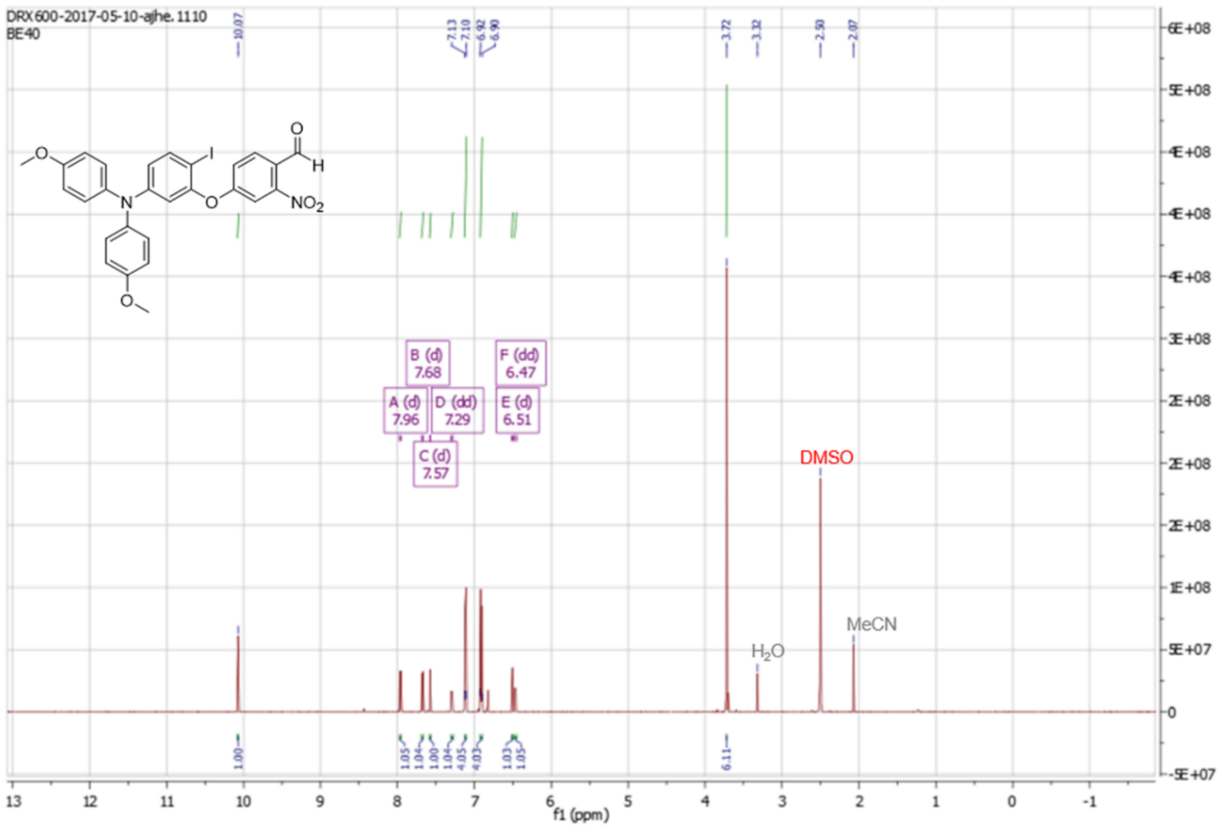

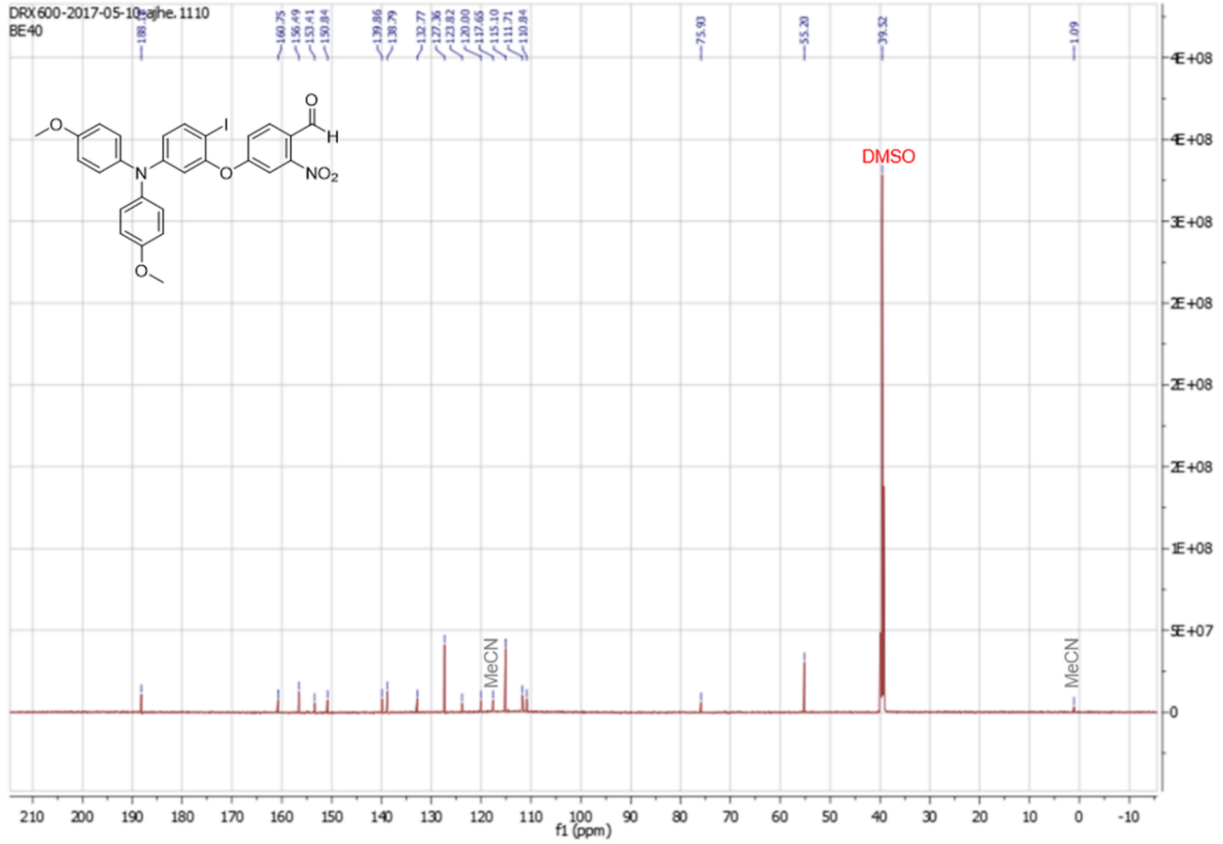

1

1-(4-(5-(di-*p*-tolylamino)-2-iodophenoxy)-2-nitrophenyl)ethan-1-ol (6b)

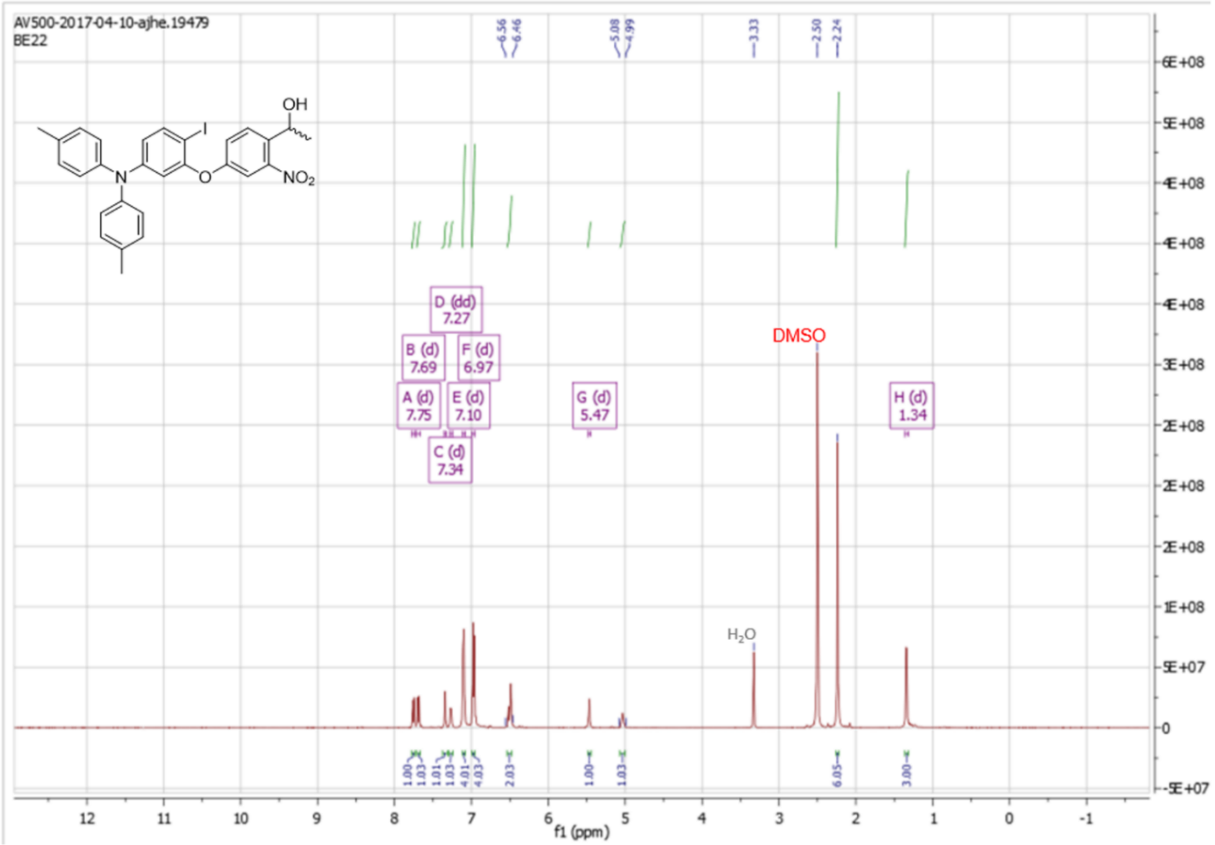

^{13}C-NMR:

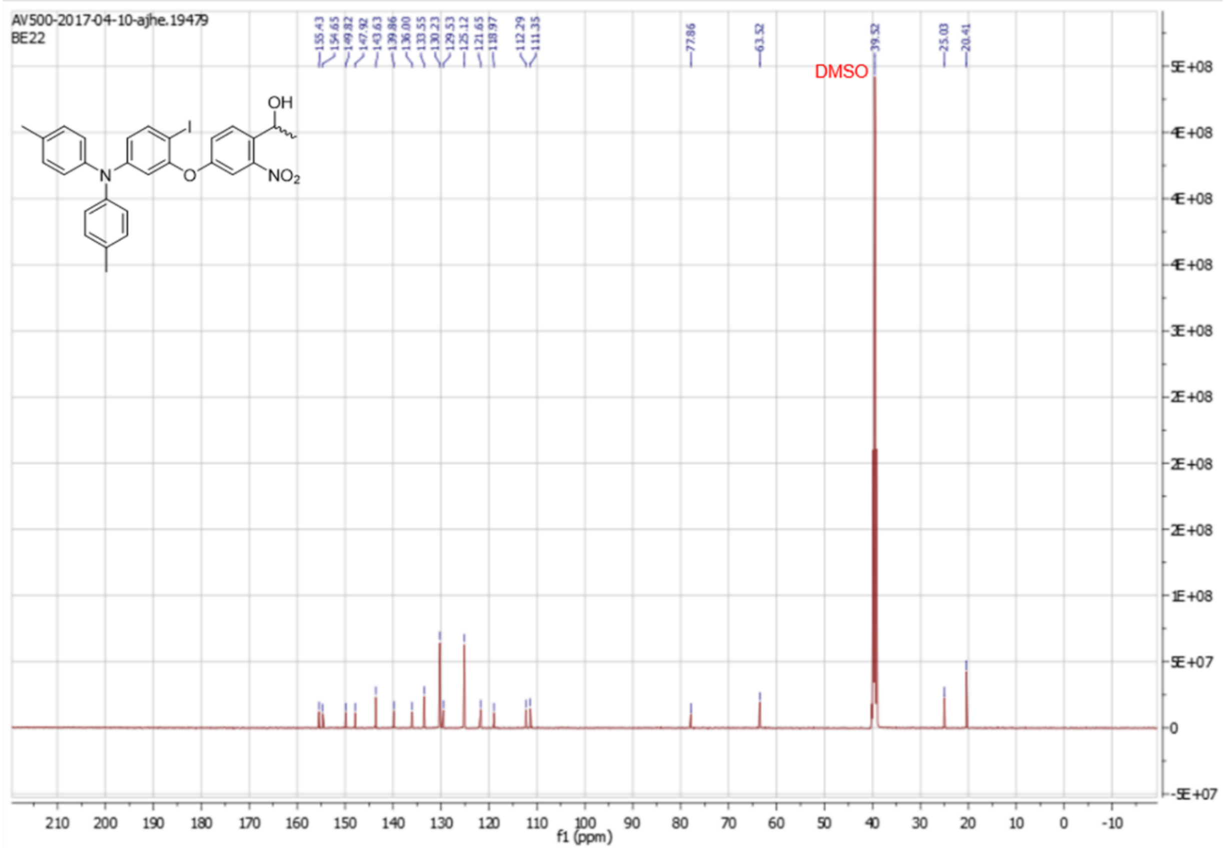

1-(4-(5-(bis(4-methoxyphenyl)amino)-2-iodophenoxy)-2-nitrophenyl)ethan-1-ol (6c)

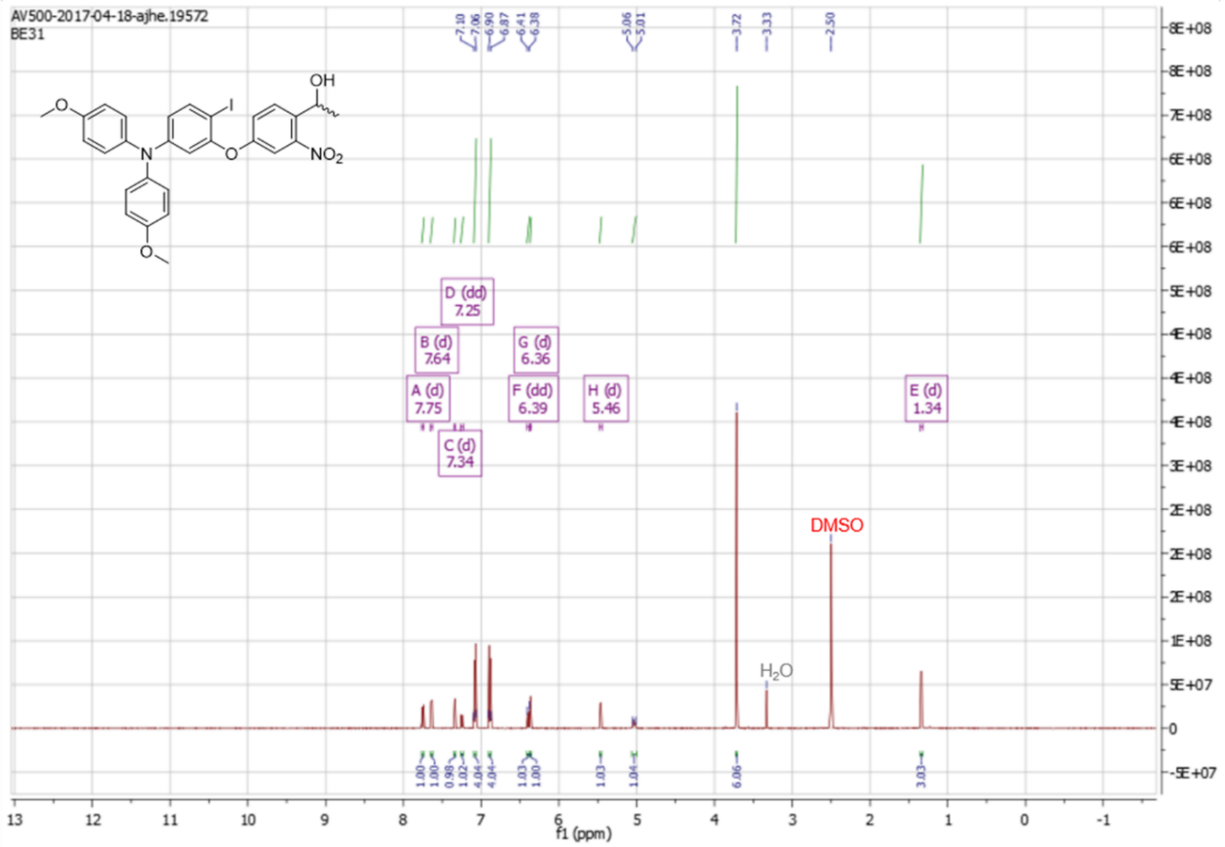

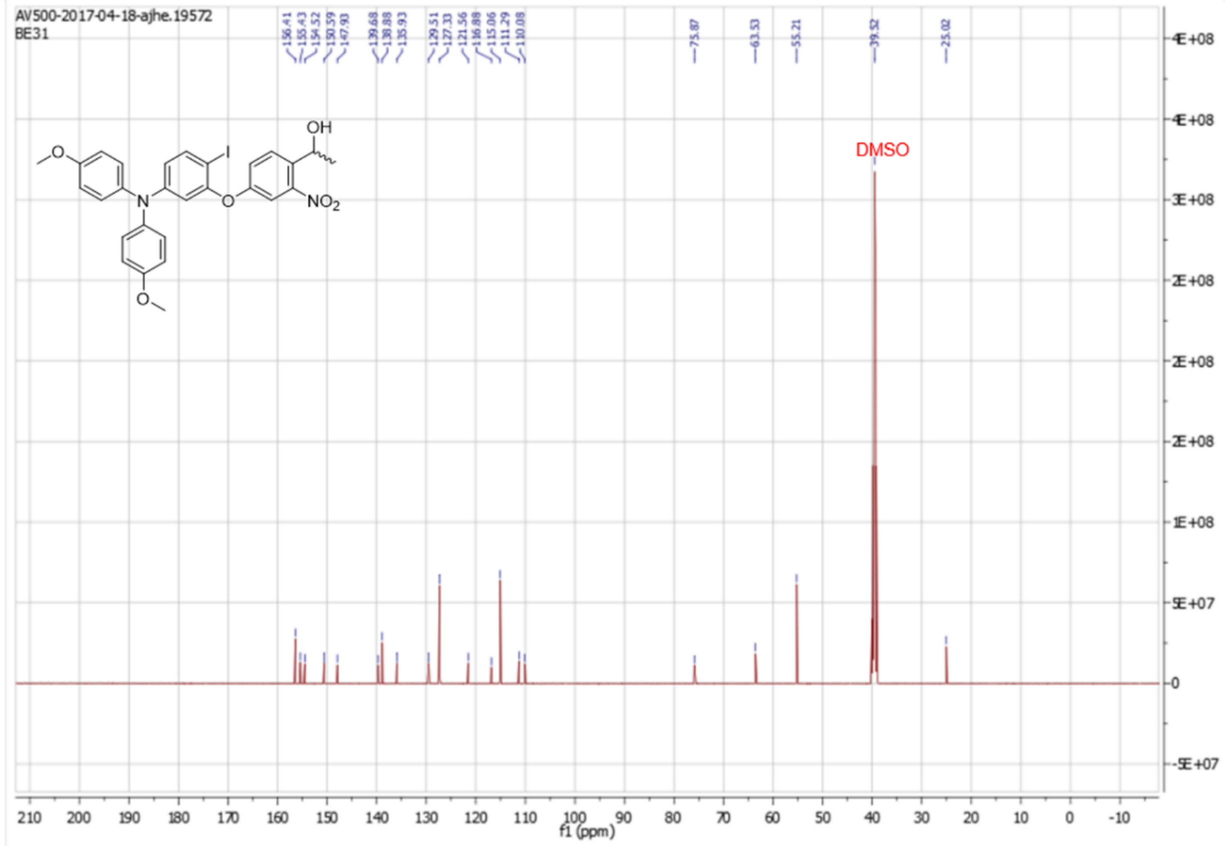

1

DTA-NDBF-OH

Derivatives b & c , R = ditolyl- & dianisyl-amino- = DTA & DAA-phenyl

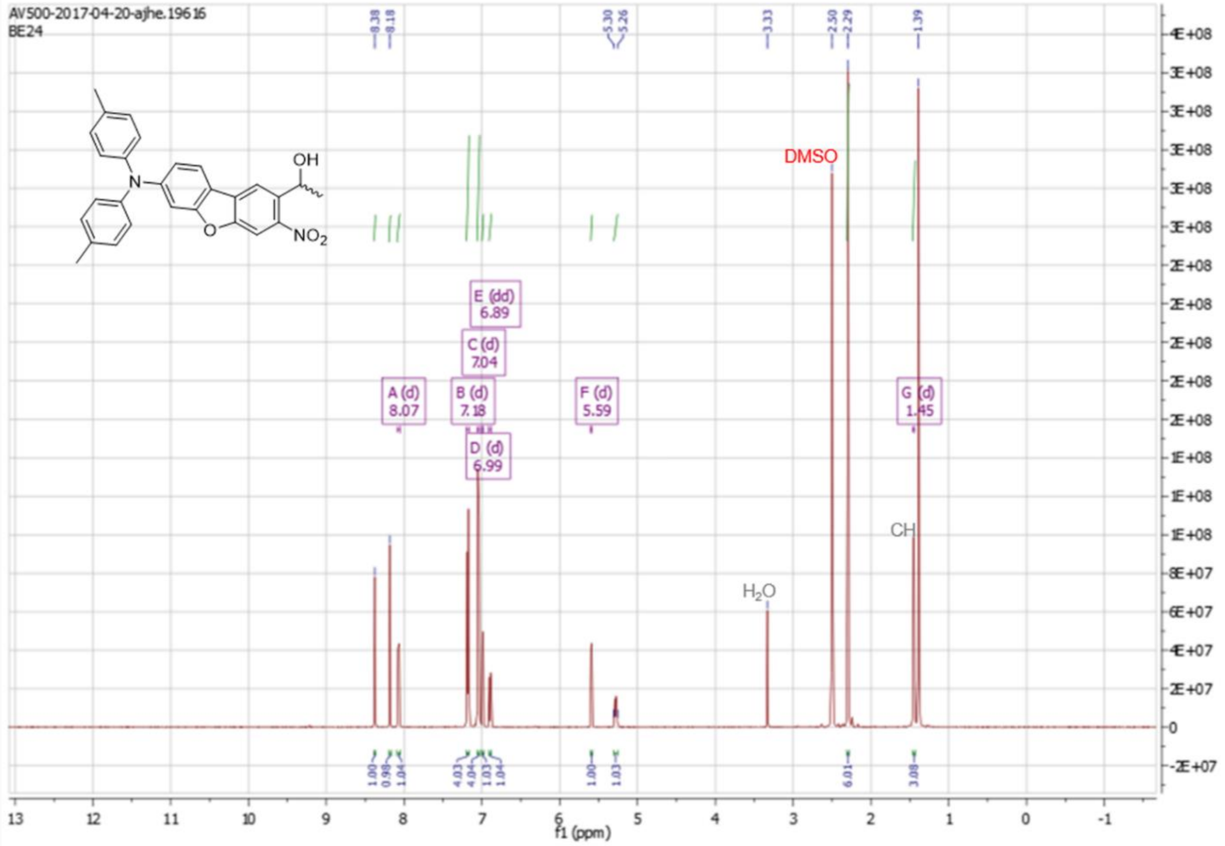

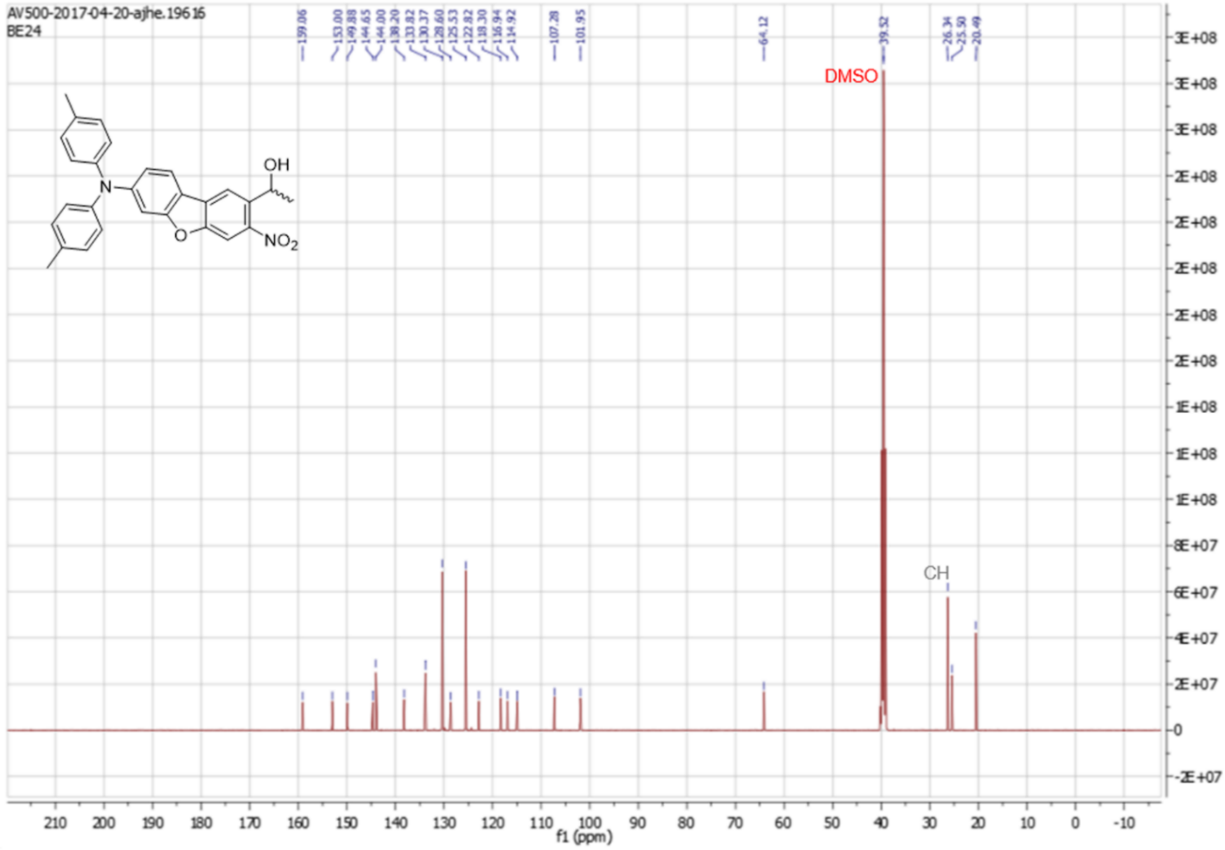

DAA-NDBF-OH Derivatives b & c , R = ditolyl- & dianisyl-amino- = DTA & DAA-phenyl

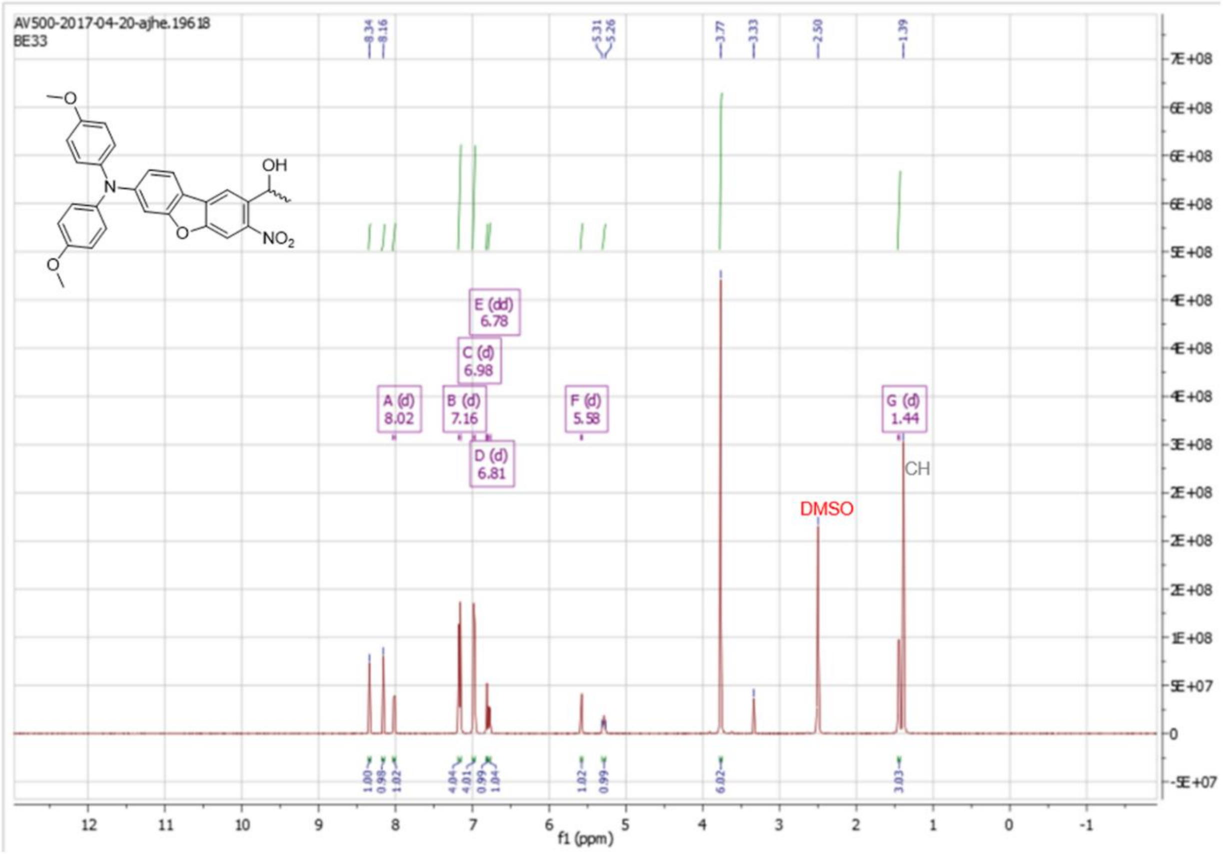

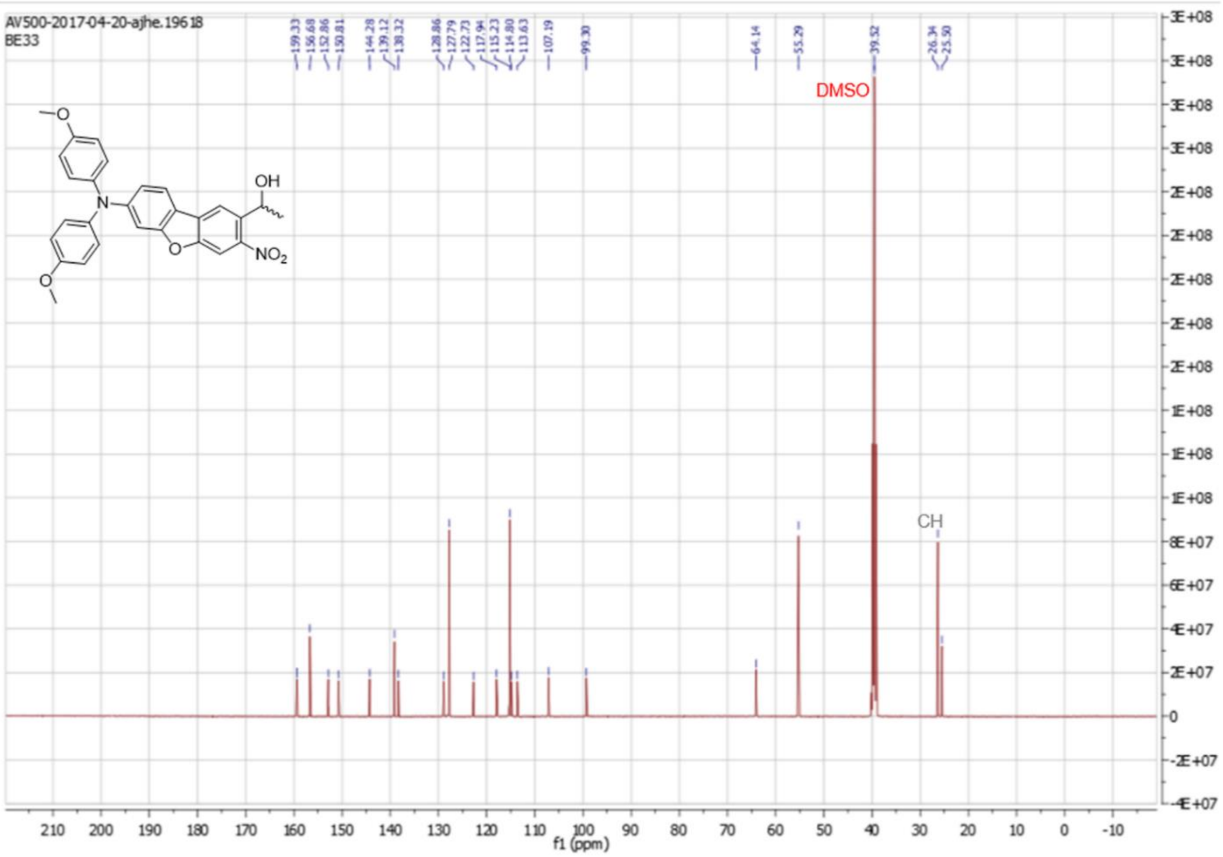

1-(7-(di-*p*-tolylamino)-3-nitrodibenzo[*b*,*d*]furan-2-yl)ethyl (4-nitrophenyl) carbonate (7b)

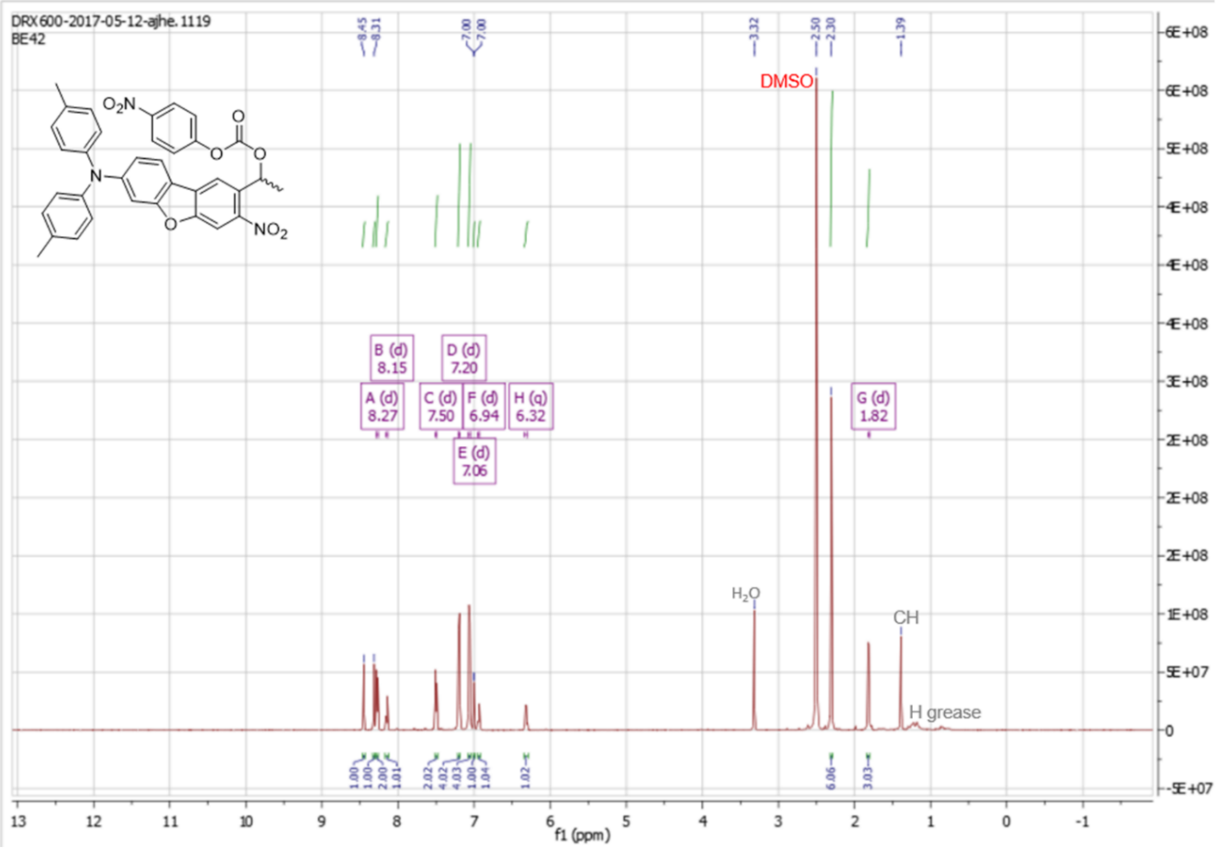

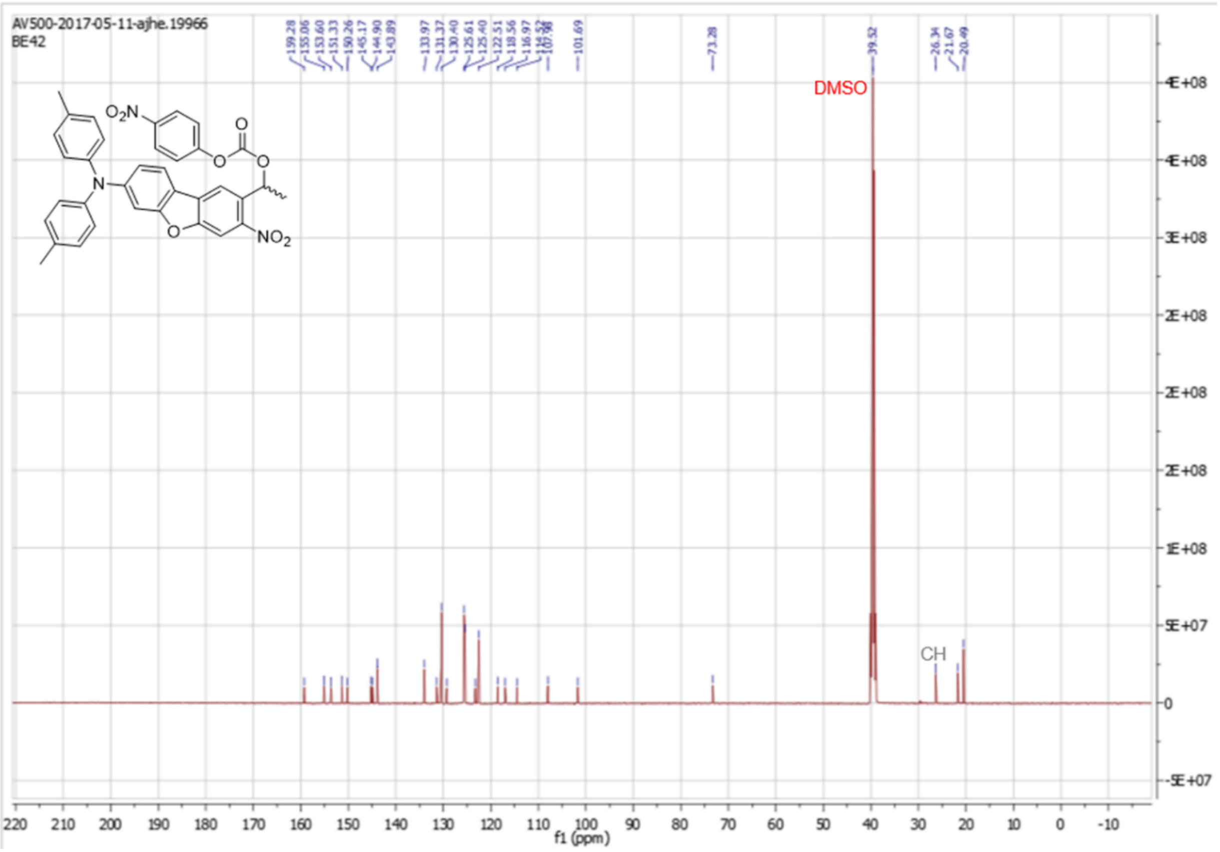

1-(7-(bis(4-methoxyphenyl)amino)-3-nitrodibenzo[*b*,*d*]furan-2-yl)ethyl (4-nitrophenyl) carbonate (7c)

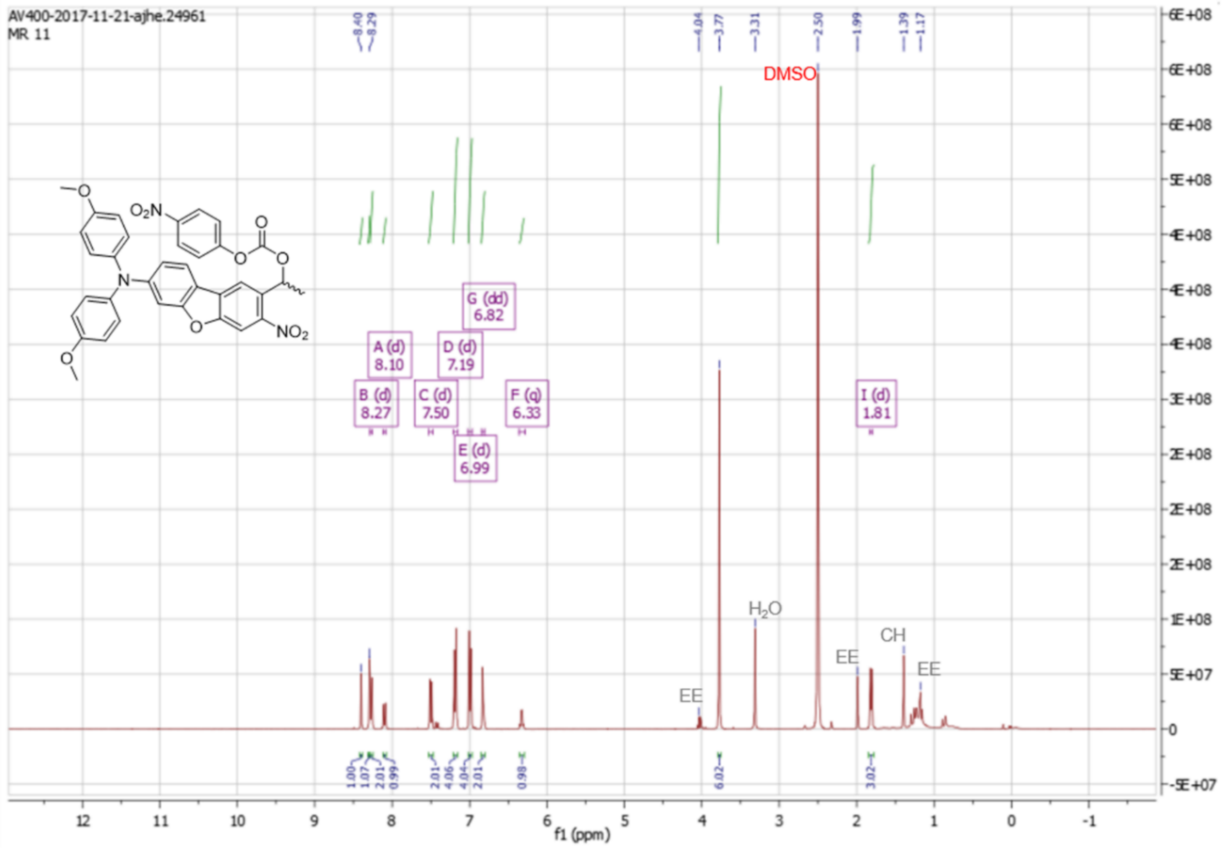

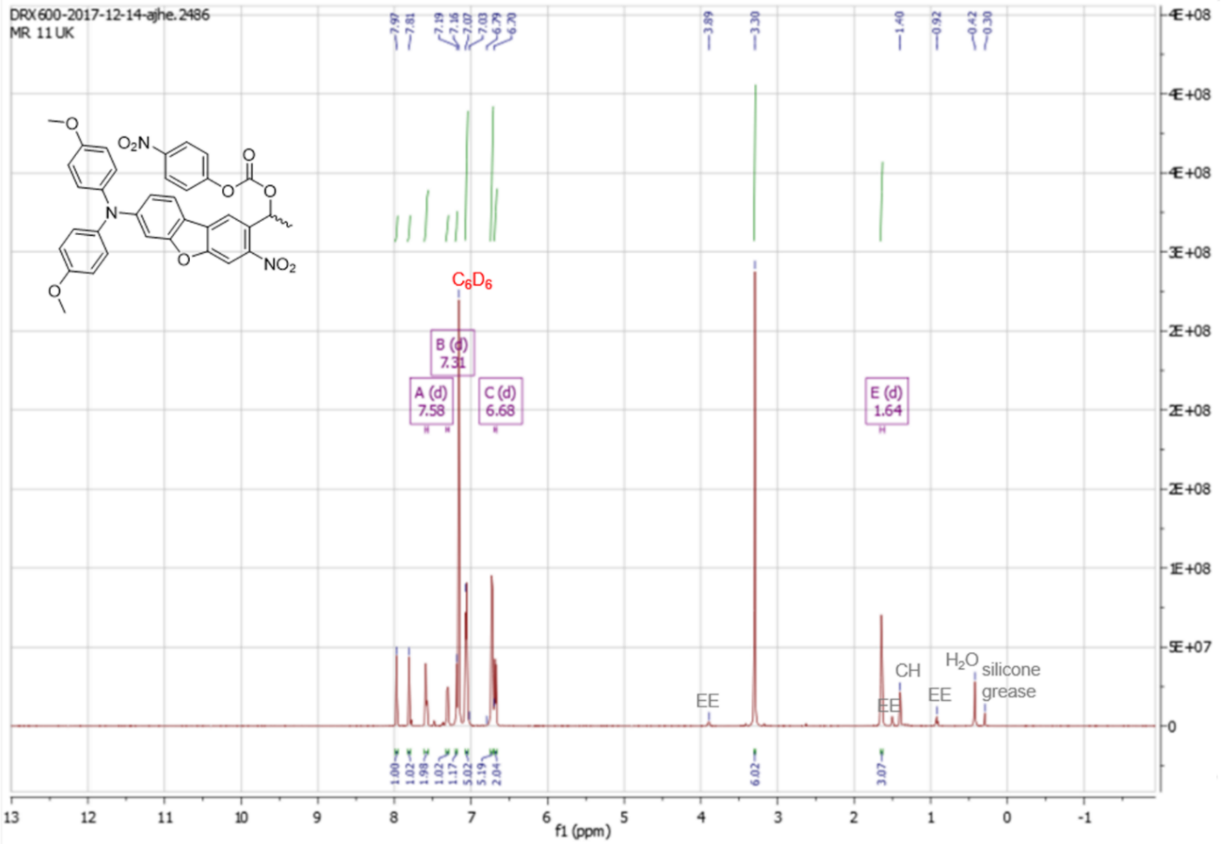

Derivatives b & c , R = ditolyl- & dianisyl-amino- = DTA & DAA-phenyl

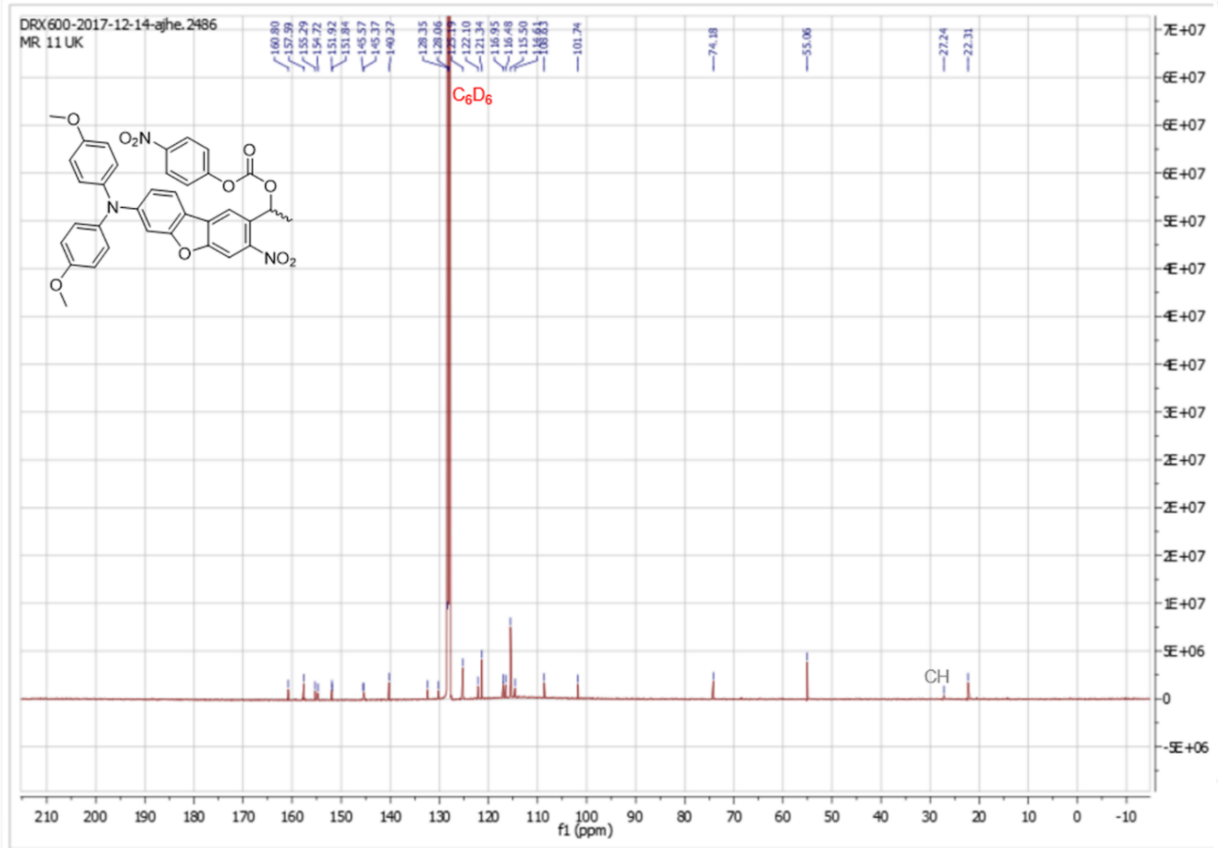

((1-(7-(di-*p*-tolylamino)-3-nitrodibenzo[*b*,*d*]furan-2-yl)ethoxy)carbonyl)-*L*-glutamic acid (DTA-NDBF-Glu)

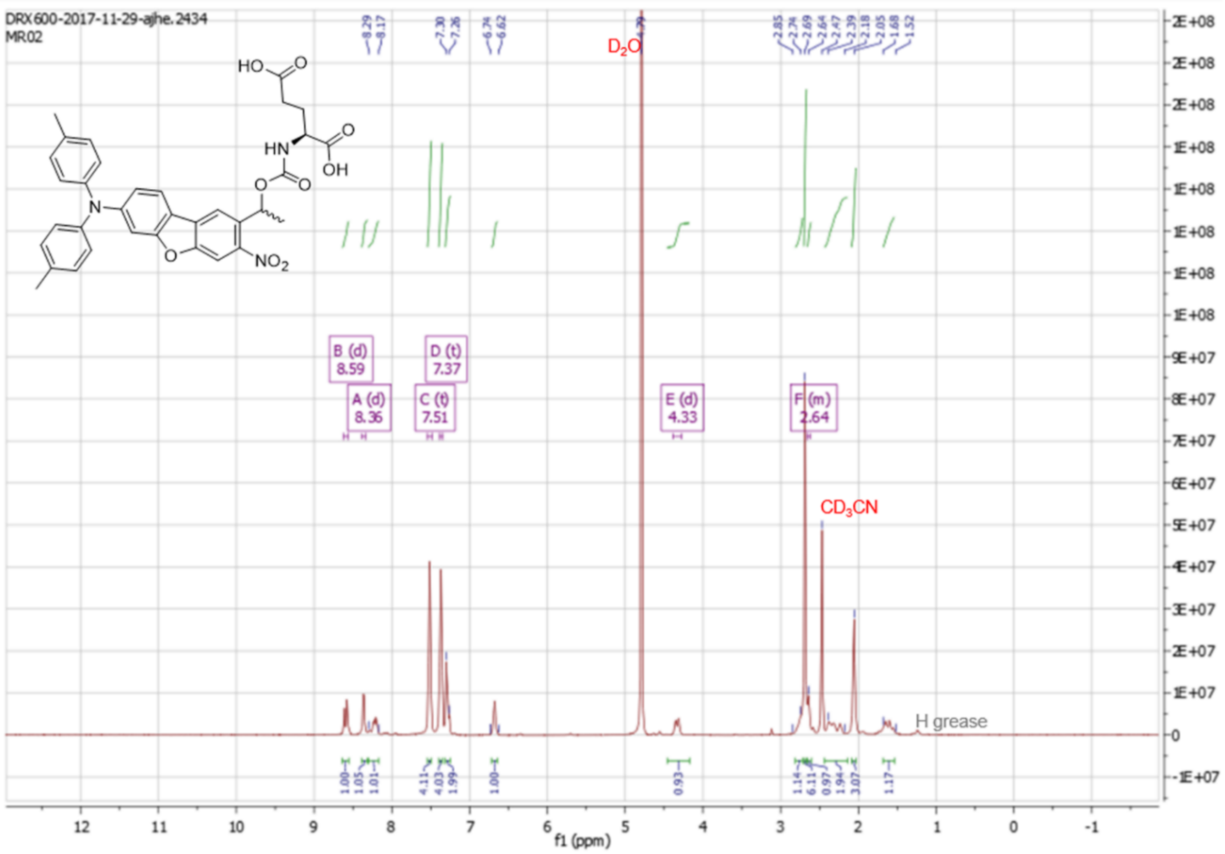

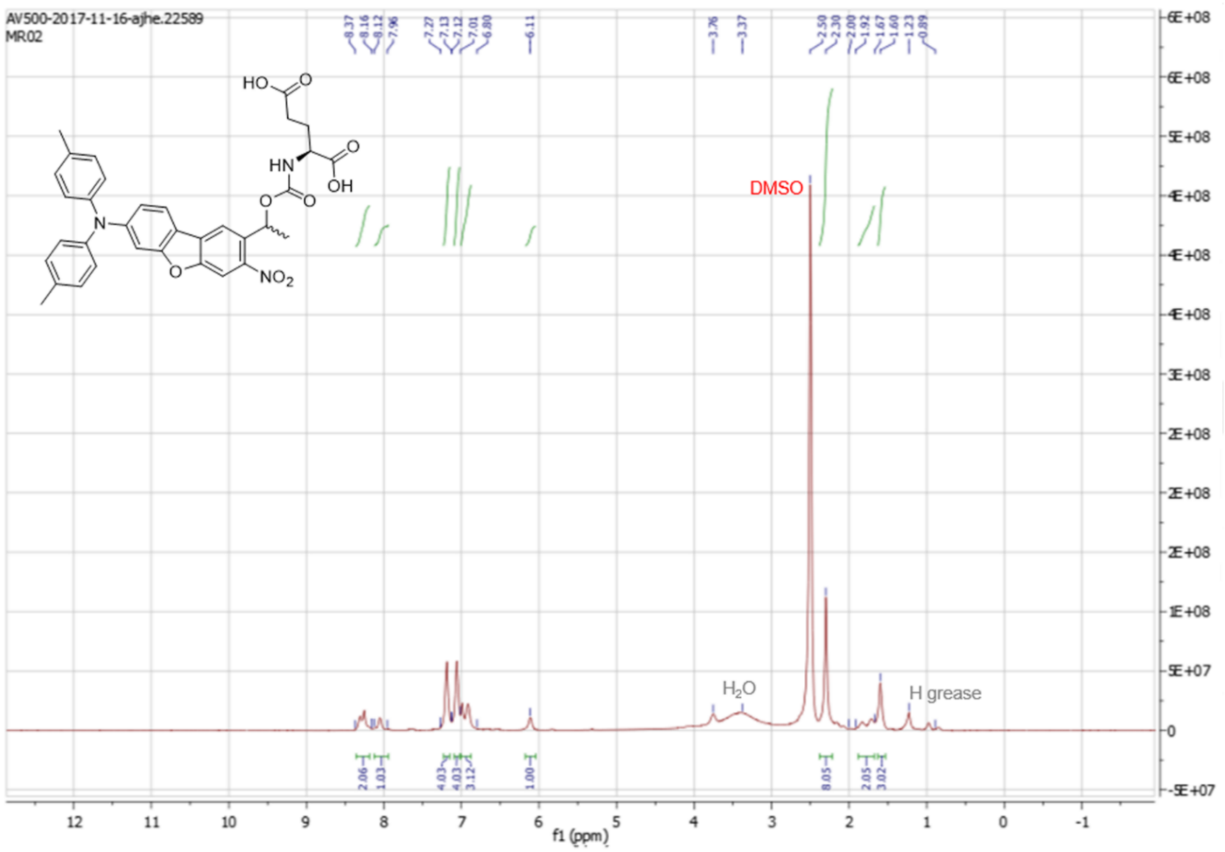

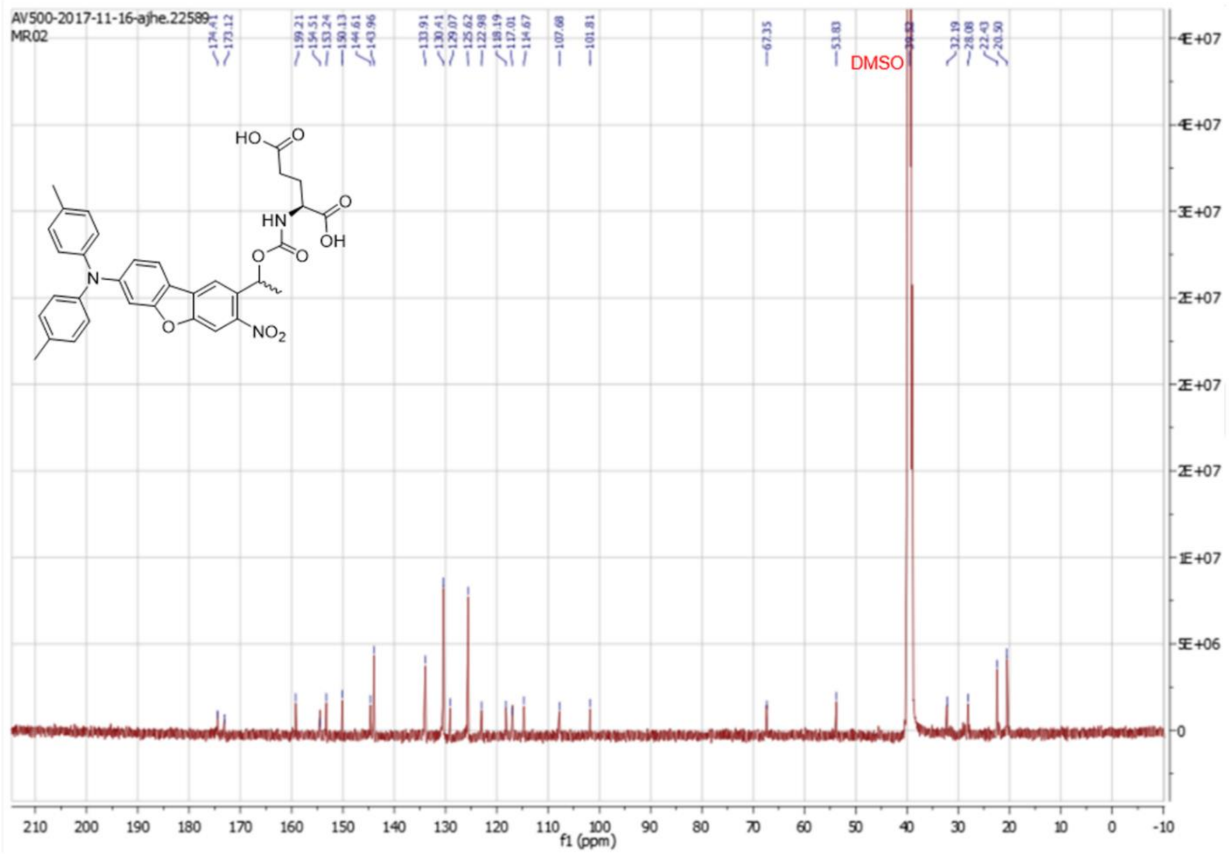

AV500-2017-11-16-ajhe.22589
MR02
DMSO
f1 (ppm)

((1-(7-(bis(4-methoxyphenyl)amino)-3-nitrodibenzo[*b*,*d*]furan-2-yl)ethoxy)carbonyl)-*L*-glutamic acid (DAA-NDBF-Glu)

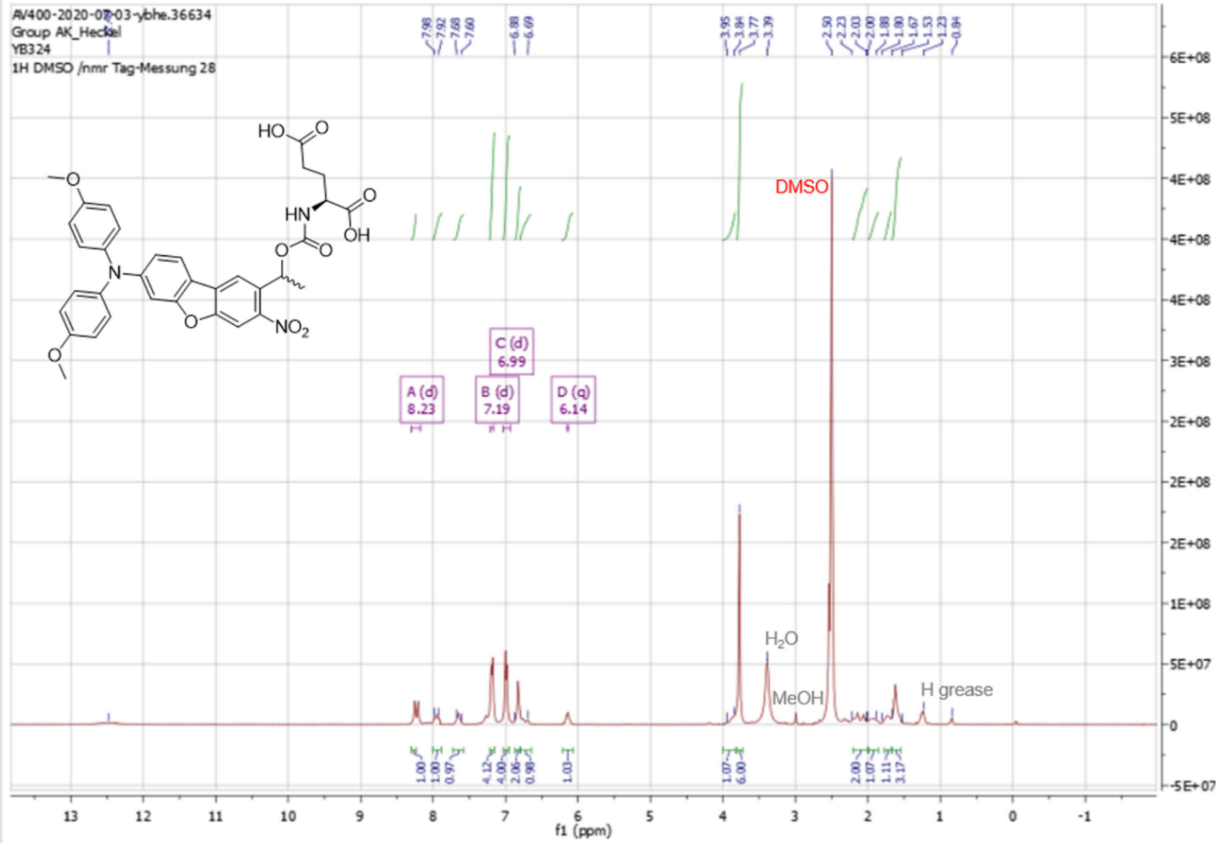

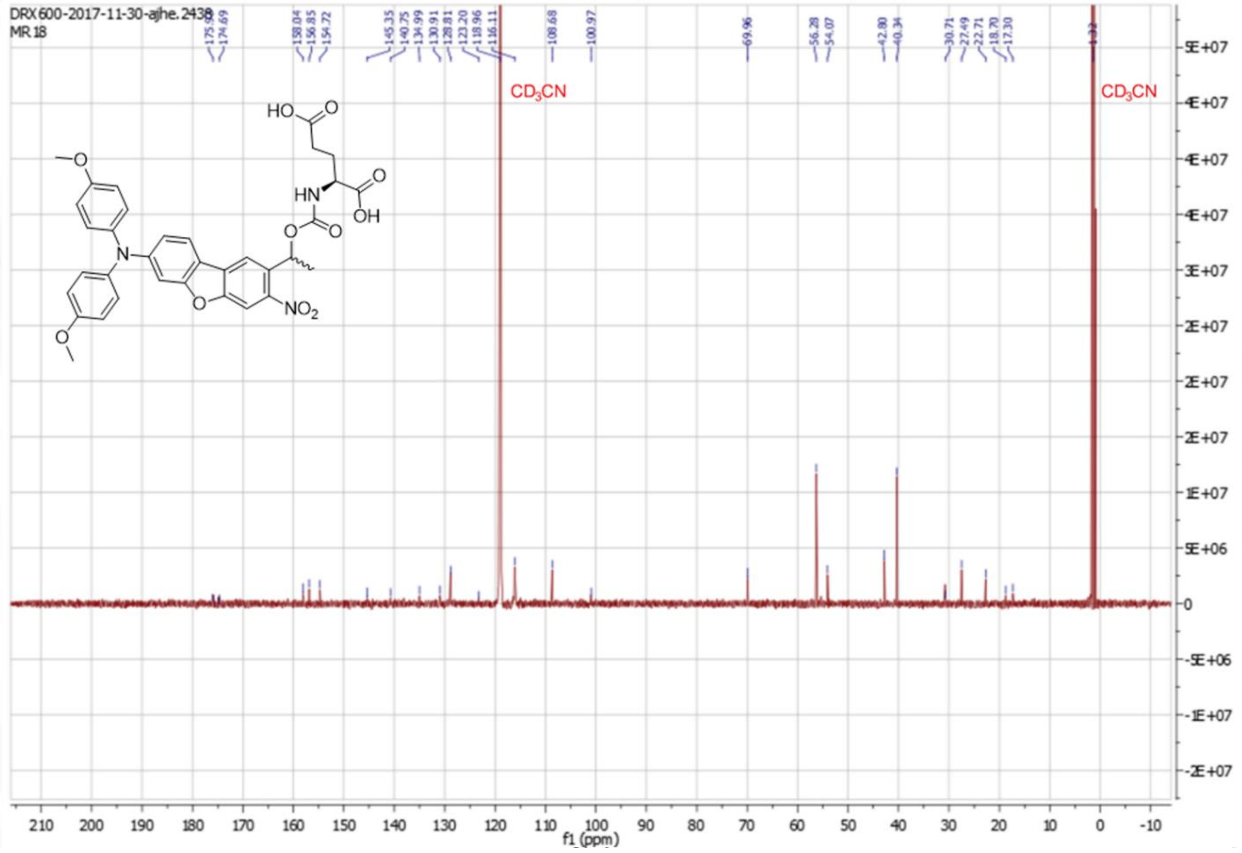

1-(7-(dimethylamino)-3-nitrodibenzo[*b*,*d*]furan-2-yl)ethyl (4-nitrophenyl) carbonate (7d)

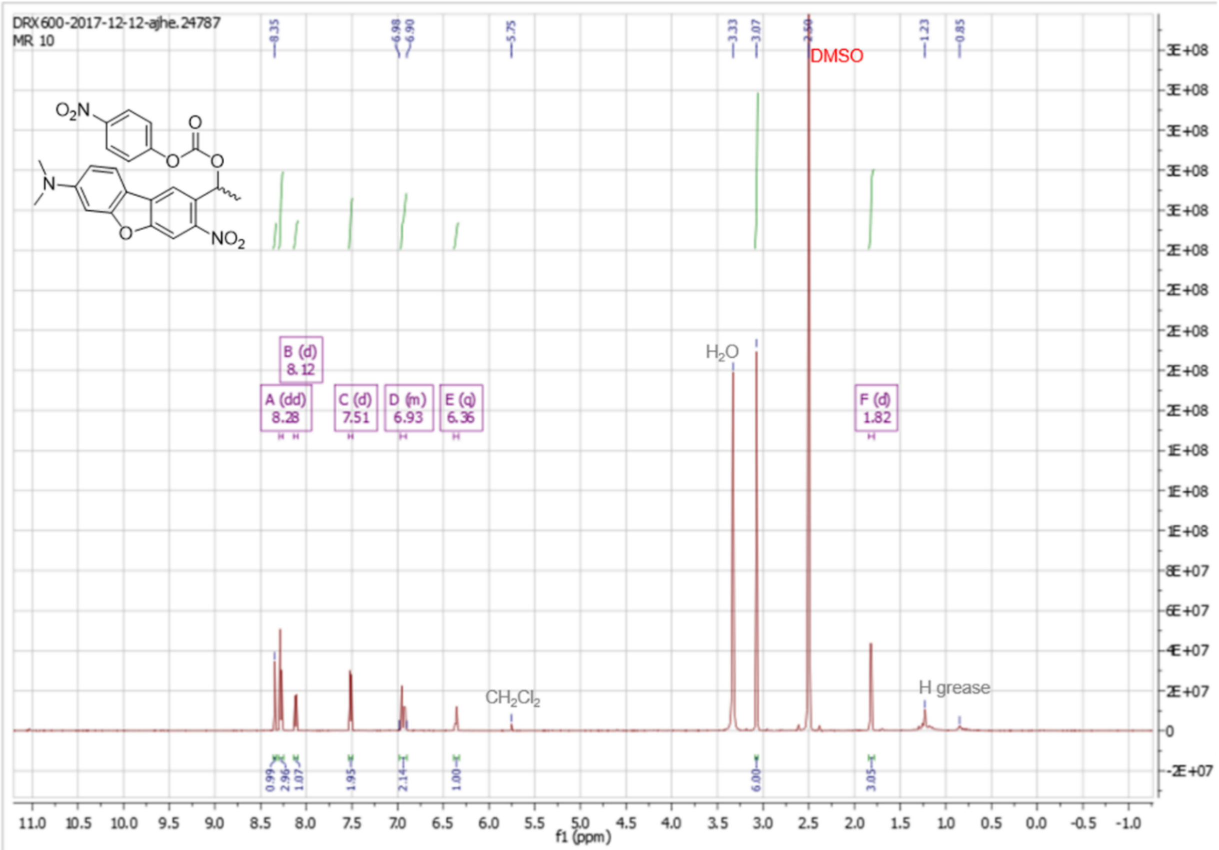

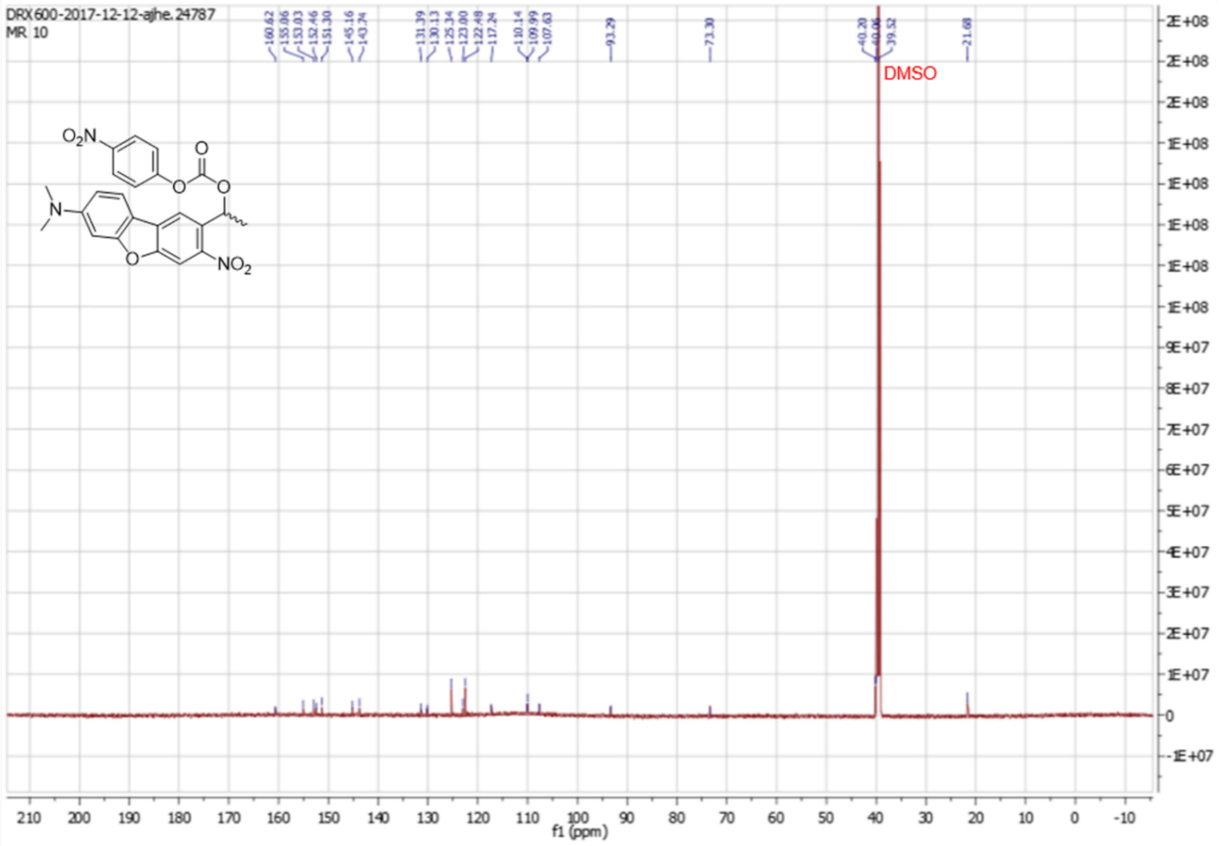

((1-(7-(dimethylamino)-3-nitrodibenzo[*b*,*d*]furan-2-yl)ethoxy)carbonyl)-*L*-glutamic acid (DMA-NDBF-Glu)

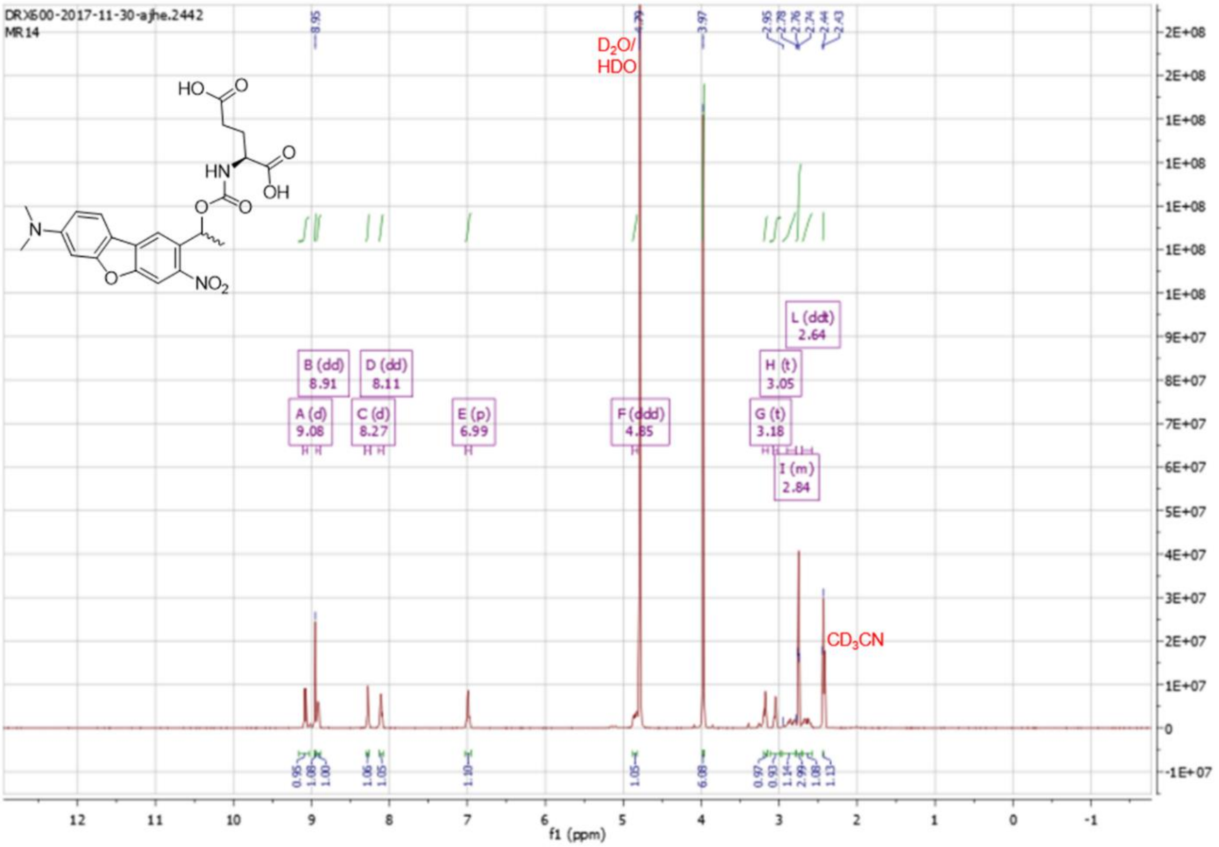

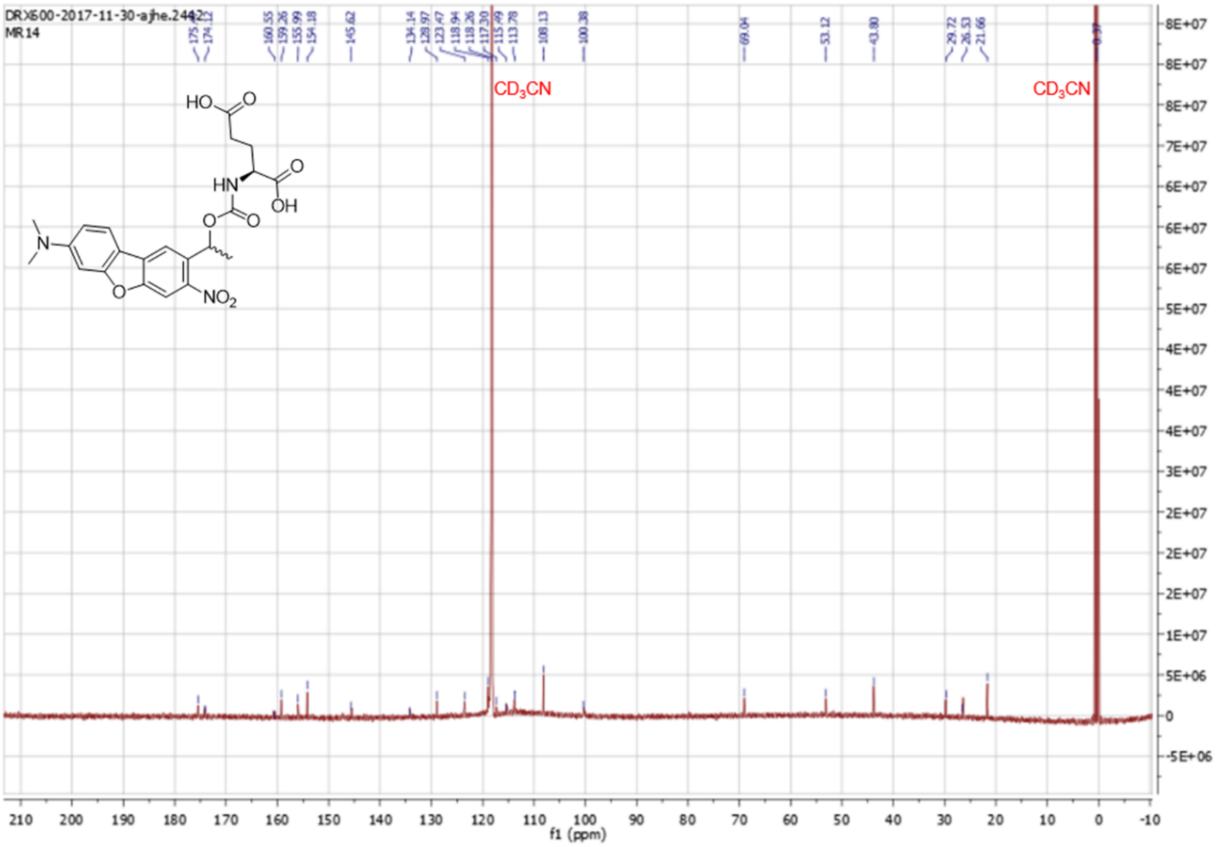

1-(3-nitrodibenzo[*b*,*d*]furan-2-yl)ethyl (4-nitrophenyl) carbonate (7e) Derivatives d, R = dimethylamino

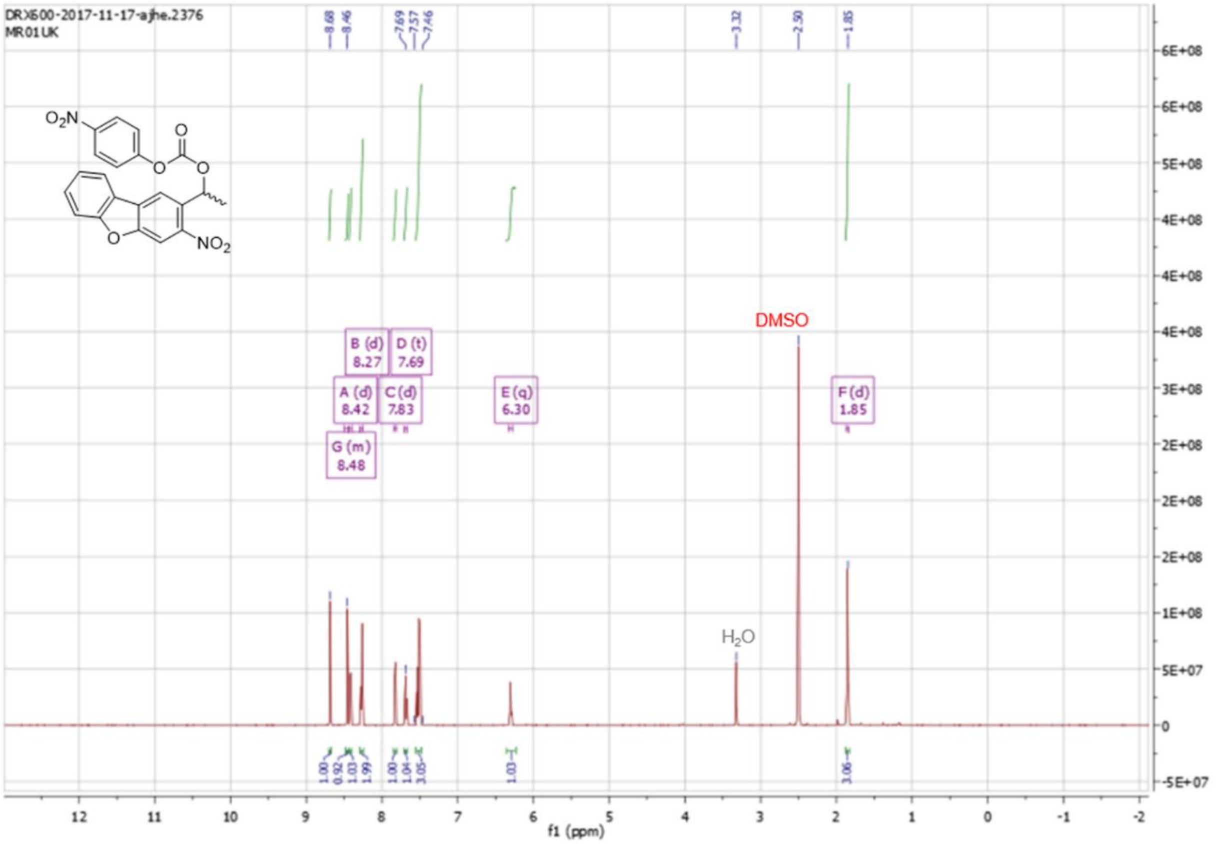

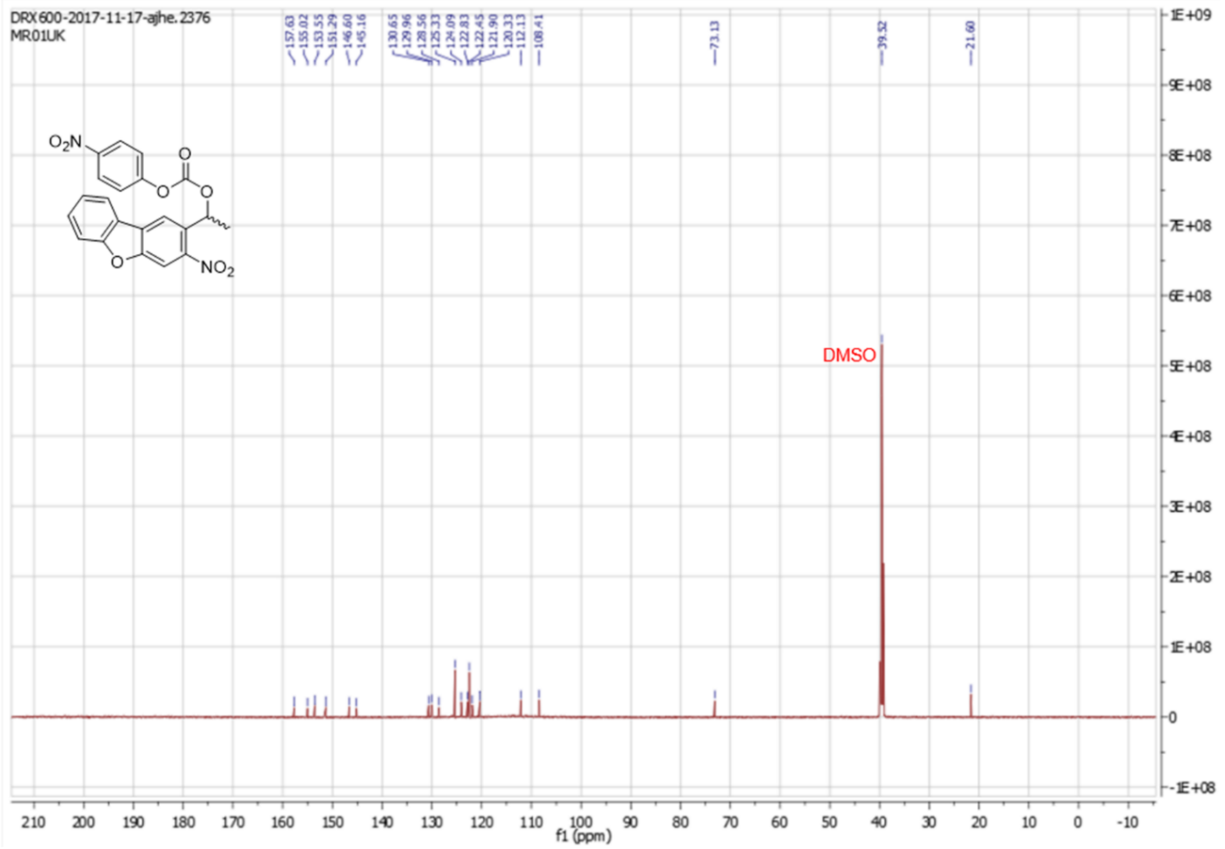

((1-(7-(dimethylamino)-3-nitrodibenzo[*b*,*d*]furan-2-yl)ethoxy)carbonyl)-*L*-glutamic acid (NDBF-Glu)

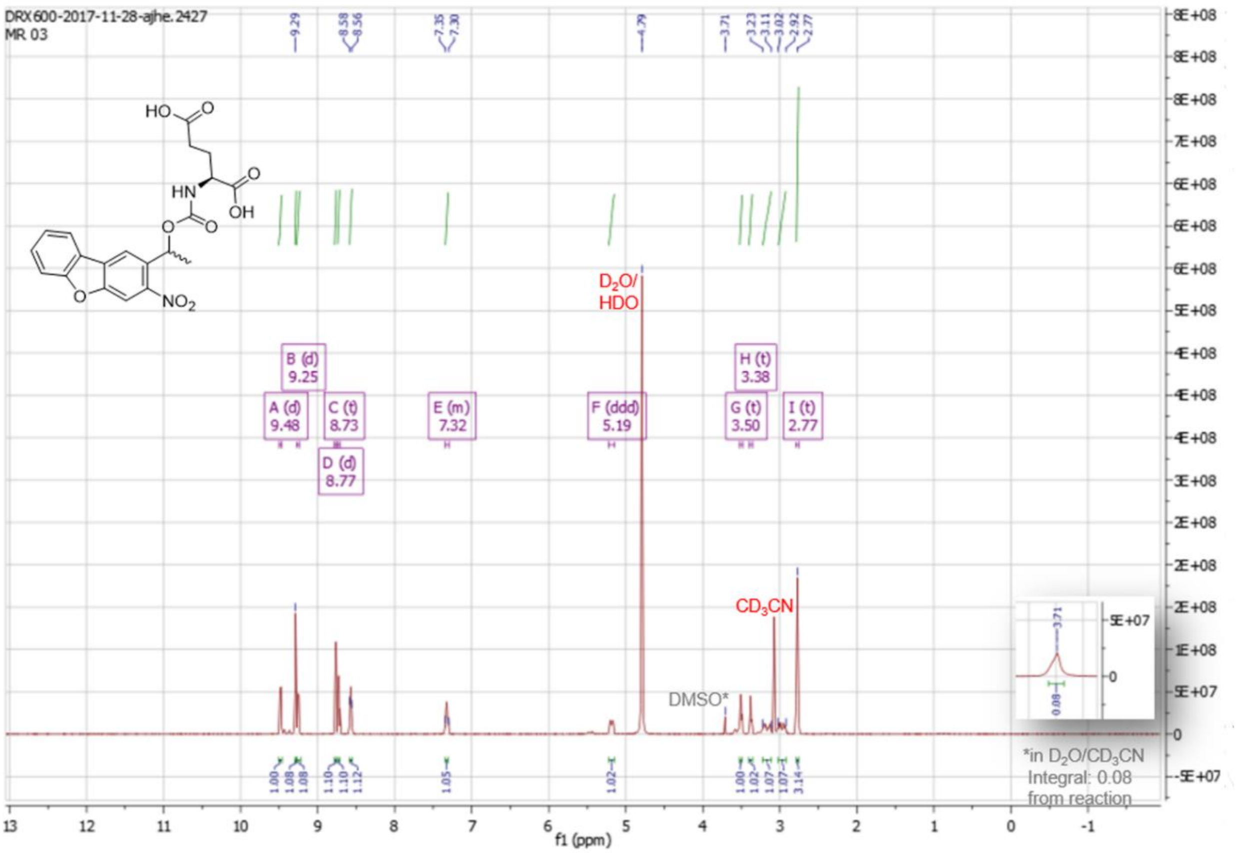

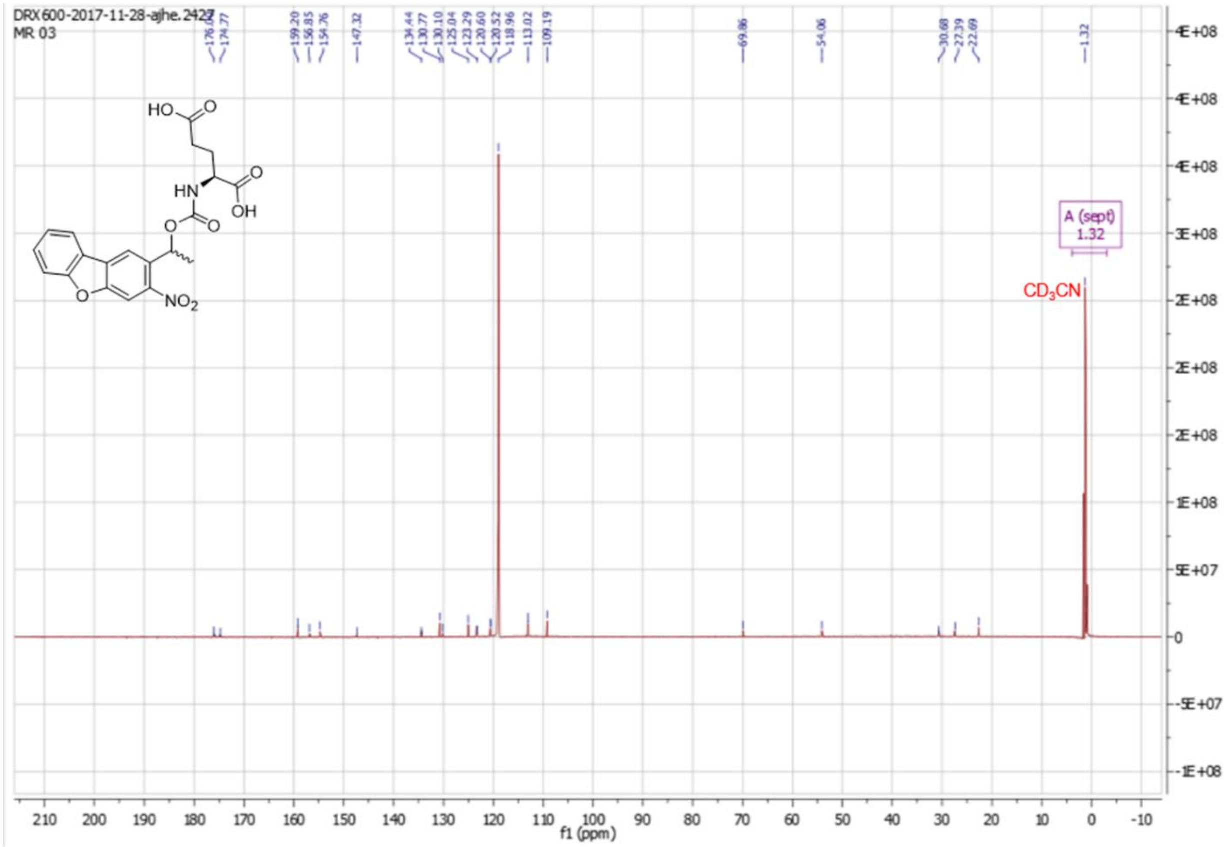

9. Mass spectra of the target compounds (x-NDBF-Glu)

MALDI-HRMS of Az-NDBF-Glu:

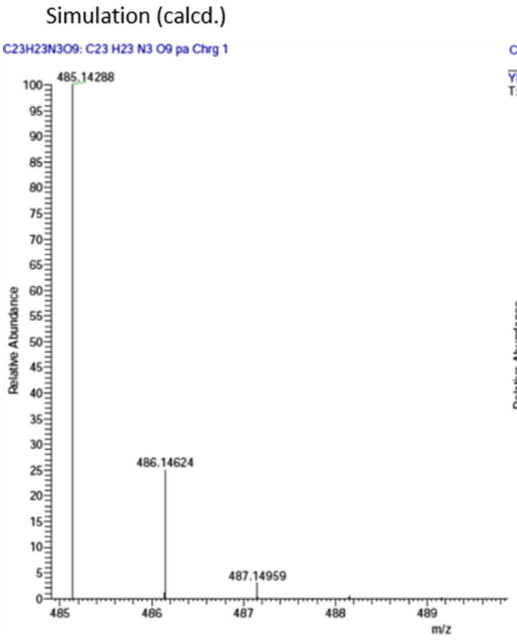

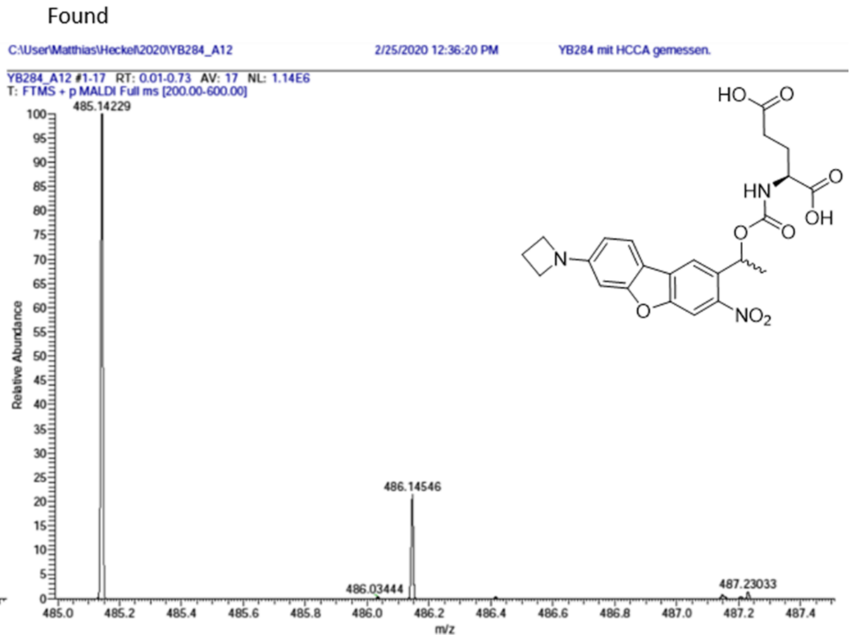

MALDI-HRMS of DTA-NDBF-Glu:

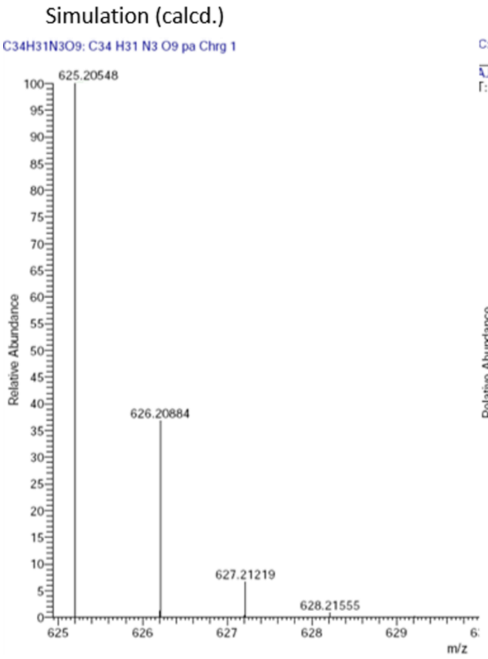

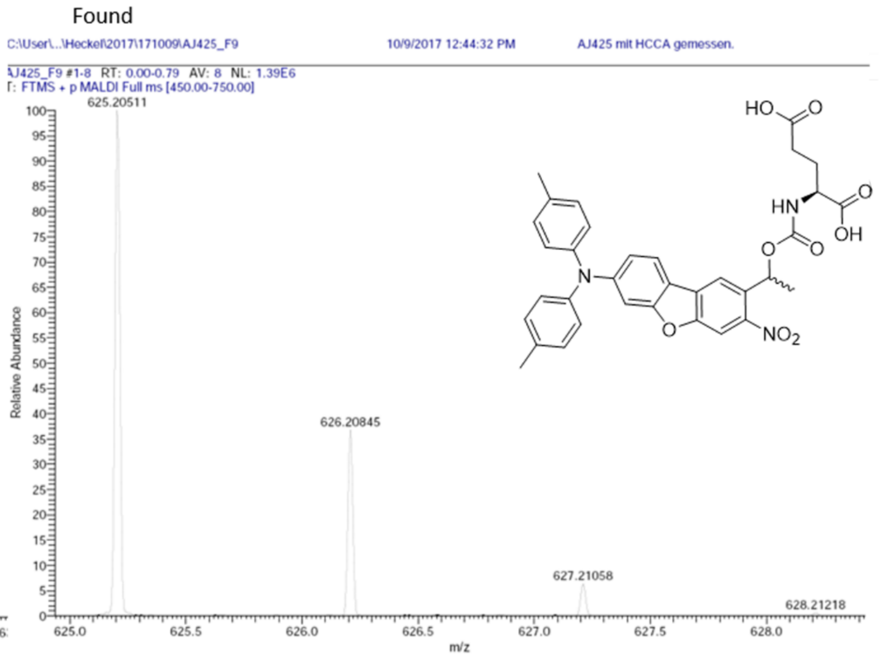

ALDI-HRMS of DAA-NDBF-Glu:

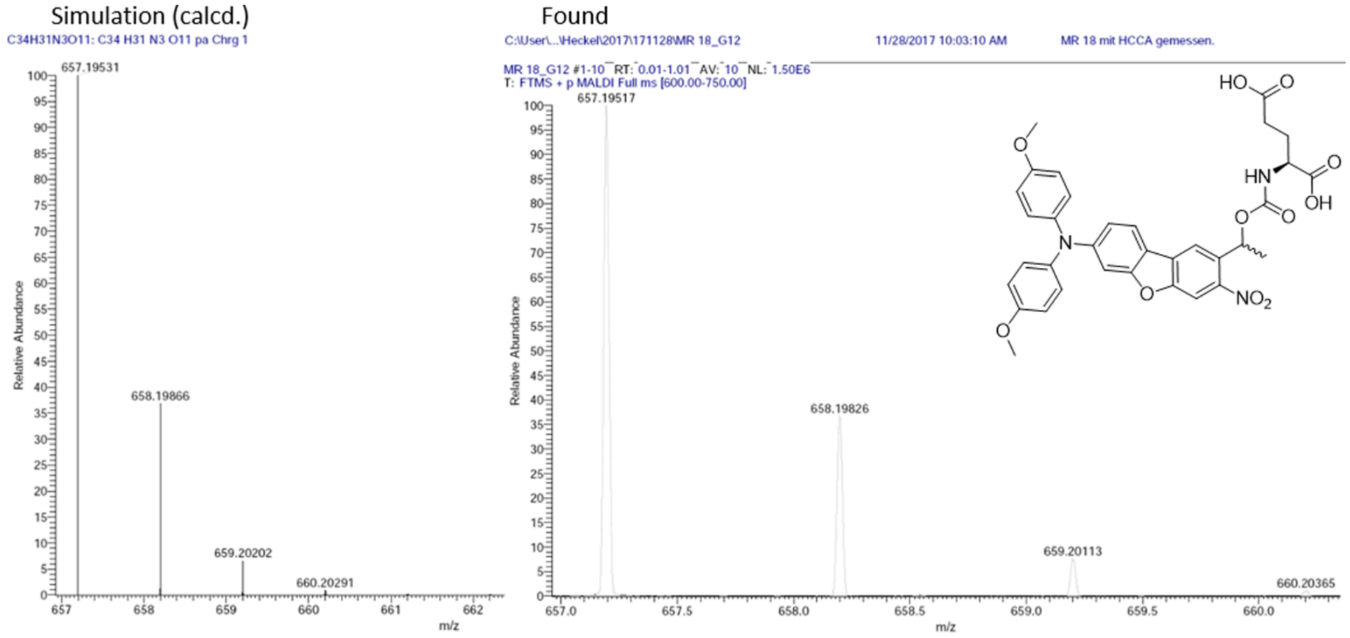

MALDI-HRMS of DMA-NDBF-Glu:

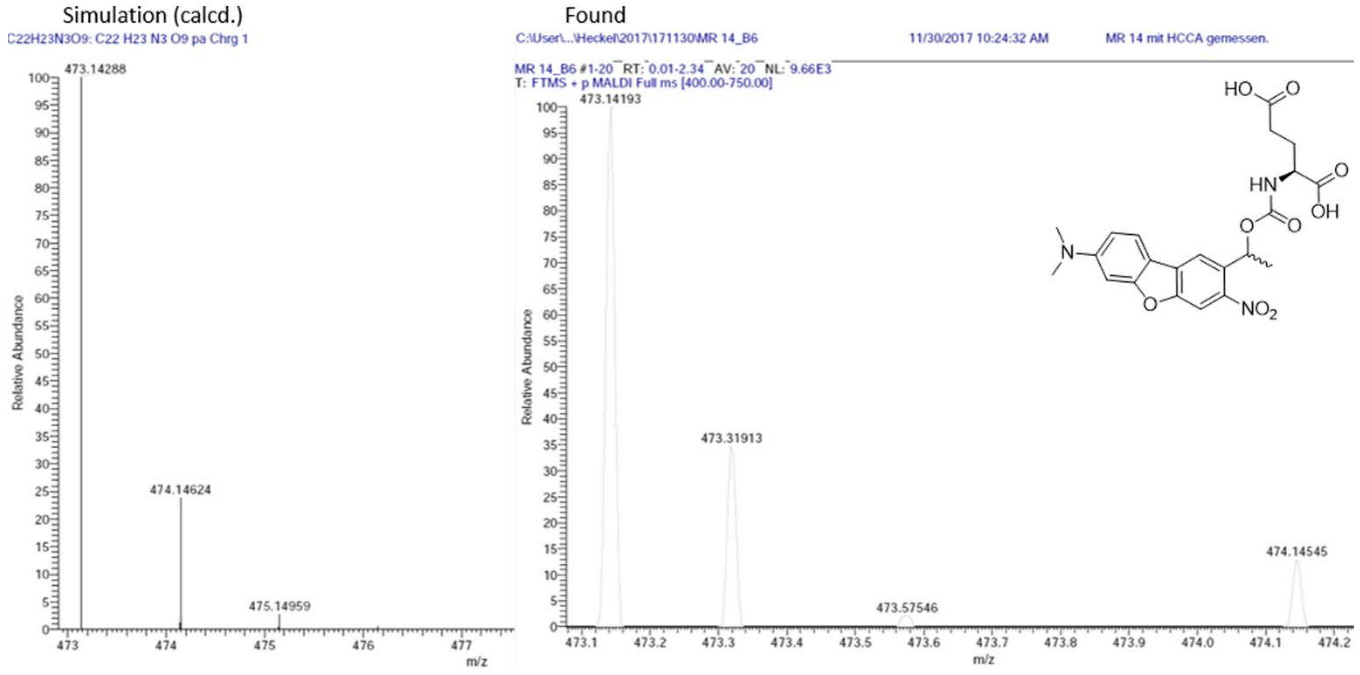

10. Overview of spectroscopic properties

Properties \ x-NDBF-OH	DMA-	Az-	DTA-	DAA-
$\lambda_{abs.max.}$[nm] in toluene	414	407	422	427
$\lambda_{abs.max.}$[nm] in MeCN	411	407	412	419
$\lambda_{abs.max.}$[nm] in isoPrOH	409	401	415	420
$\lambda_{abs.max.}$[nm] in MeOH	409	402	410	418
$\lambda_{em.max.}$[nm] in toluene	568	558	581	588
$\lambda_{em.max.}$[nm] in isoPrOH	596	572	572	581
$\lambda_{em.max.}$[nm] in MeOH	-	580	-	580
Stokes shift $\Delta\lambda/\Delta\tilde{\nu}$ in toluene [nm/cm^{-1}]	154/6549	151/6649	159/6485	161/6412
Stokes shift $\Delta\lambda/\Delta\tilde{\nu}$ in isoPrOH [nm/cm^{-1}]	187/7671	171/7455	157/6614	161/6598
Stokes shift $\Delta\lambda/\Delta\tilde{\nu}$ in MeOH [nm/cm^{-1}]	-	178/7634	-	162/6682
$\Phi_{fl.}$ [%] in toluene	1.10	0.95	n.d.	n.d.
$\Phi_{fl.}$ [%] in isoPrOH	0.18	0.38	n.d.	n.d.
$\Phi_{fl.}$ [%] in MeOH	0	>0.10	n.d.	n.d.

Fluorescence quantum yields Φfl of **DMA-NDBF-OH** and **Az-NDBF-OH** in toluene, isopropanol and methanol (weak signals) were obtained by using an integrating sphere

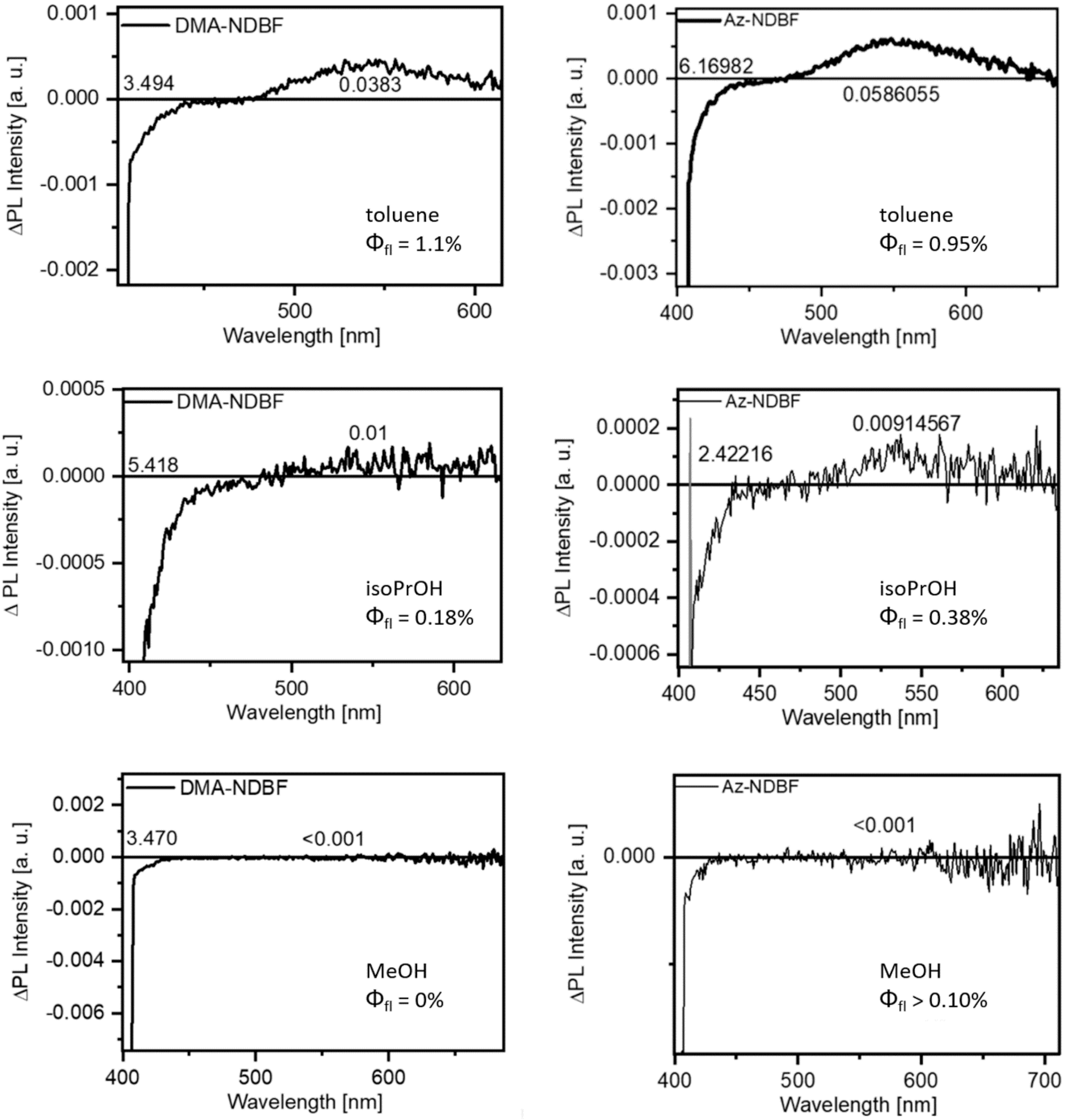

Supplement: Supplementary file 1 — Supplementary [file CHEM-27-2212-s001.pdf]
